# Evaluation of a non-animal toolbox informed by adverse outcome pathways for human inhalation safety

**DOI:** 10.3389/ftox.2025.1426132

**Published:** 2025-02-21

**Authors:** Renato Ivan de Ávila, Iris Müller, Hugh Barlow, Alistair Mark Middleton, Mathura Theiventhran, Danilo Basili, Anthony M. Bowden, Ouarda Saib, Patrik Engi, Tymoteusz Pietrenko, Joanne Wallace, Bernadett Boda, Samuel Constant, Holger Peter Behrsing, Vivek Patel, Maria Teresa Baltazar

**Affiliations:** ^1^ Safety, Environmental and Regulatory Science (SERS), Unilever, Colworth Science Park, Sharnbrook, Bedfordshire, United Kingdom; ^2^ Charles River Laboratories, Edinburgh, United Kingdom; ^3^ Epithelix Sarl, Plan-les-Outes, Switzerland; ^4^ Respiratory Toxicology Program, Institute for In Vitro Sciences, Inc., Gaithersburg, MD, United States

**Keywords:** inhalation risk assessment, lung exposure modelling, lung toxicity, new approach methodologies, nonanimal testing, point of departure, bioactivity exposure ratio

## Abstract

**Introduction:**

This work evaluated a non-animal toolbox to be used within a next-generation risk assessment (NGRA) framework to assess chemical-induced lung effects using human upper and lower respiratory tract models, namely MucilAir™-HF and EpiAlveolar™ systems, respectively.

**Methods:**

A 12-day substance repeated exposure scheme was established to explore potential lung effects through analysis of bioactivity readouts from the tissue integrity and functionality, cytokine/chemokine secretion, and transcriptomics.

**Results:**

Eleven benchmark chemicals were tested, including inhaled materials and drugs that may cause lung toxicity following systemic exposure, covering 14 human exposure scenarios classified as low- or high-risk based on historical safety decisions. For calculation of bioactivity exposure ratios (BERs), obtained chemical-induced bioactivity data were used to derive *in vitro* points of departures (PoDs) using a nonlinear state space model. PoDs were then combined with human exposure estimates, i.e., predicted lung deposition for benchmark inhaled materials using multiple path particle dosimetry (MPPD) exposure computational modeling or literature maximum plasma concentration (C_max_) for systemically available benchmark drugs.

**Discussion:**

In general, PoDs occurred at higher concentrations than the corresponding human exposure values for the majority of the low-risk chemical-exposure scenarios. For all the high-risk chemical-exposure scenarios, there was a clear overlap between the PoDs and lung deposited mass and C_max_ for the benchmark inhaled materials and therapeutic drugs, respectively. Our findings suggest that combining computational and *in vitro* new approach methodologies (NAMs) informed by adverse outcome pathways (AOPs) associated with pulmonary toxicity can provide relevant biological coverage for chemical lung safety assessment.

## 1 Introduction

Unlike nebulized pharmaceuticals which are inhaled for therapeutic reasons, consumer spray products (e.g., antiperspirants, hairsprays, cleaning sprays) do not need to be inhaled to perform their function but may lead to unintentional inhalation exposure during normal daily use. It is therefore important for the safety assessment of such consumer products to consider the potential for ingredients to cause adverse effects in the lung under the conditions of product use. Lung hazard data have historically been obtained by performing testing in animals. In the case of inhaled materials, animal data have been taken from studies that consist of exposing rodents to chemicals in whole-body or nose-only systems, such as rodent acute (OECD, 2009a; OECD, 2009b; OECD, 2018c), 28-day subacute (OECD, 2018a), and 90-day subchronic (OECD, 2018b; US EPA, 1998) inhalation toxicity studies from which no observed effect concentration (NOEC) or no observed adverse effect concentration (NOAEC) are derived to inform human health inhalation risk assessments (Ramanarayanan et al., 2022).

*In vivo* studies have been used in risk assessment for many decades. However, it is clear that significant uncertainties arise from using animal data in the prediction of human toxicity responses due to anatomical, physiological, and biochemical differences between rodent and human respiratory systems and differences in breathing patterns (Cao et al., 2021; Clippinger et al., 2018b). Additionally, ethics related to animal welfare, and changes in the legislation in several geographies have motivated the development of more human-relevant tools and approaches that do not rely on the generation of new animal data to test chemicals (Brescia et al., 2023; Fentem, 2023). For example, animal testing of cosmetic ingredients has been banned in the European Union (EU) since 2009 (European Union, 2009). Other legislation, including the REACH (Registration, Evaluation and Authorization of Chemicals) regulation in the EU, also clearly states that registrants should only use animal testing as a last resort to obtain chemical hazard and safety information (European Commission, 2006).

In this context, recent research anchored in human-relevant science has focused on developing human-relevant *in silico* and *in vitro* tools and approaches (New Approach Methodologies, NAMs) that can be employed, together with existing information, within the next-generation risk assessment (NGRA) of materials to assess the risk of lung toxicity (Bedford et al., 2022; Clippinger et al., 2018b; Corley et al., 2021; OECD, 2022; Ramanarayanan et al., 2022; Stucki et al., 2022). NGRA aims to conduct safety assessments that are “human-relevant, exposure-led, hypothesis-driven and designed to prevent harm” (Dent et al., 2018). Therefore, NGRA focus on determining whether a substance triggers *in vitro* bioactivity at human-relevant concentrations and, consequently, adverse health effects, *in lieu* of replicating apical endpoints that would traditionally be obtained using high-dose animal studies (Baltazar et al., 2020; Dent et al., 2021; Middleton et al., 2022; OECD, 2017; Thomas et al., 2019). NGRA uses information from NAMs and conservative decision-making to protect human health, aligning with the way regulatory decision-making has been conducted using traditional approaches (Browne et al., 2024).

**FIGURE 1:**
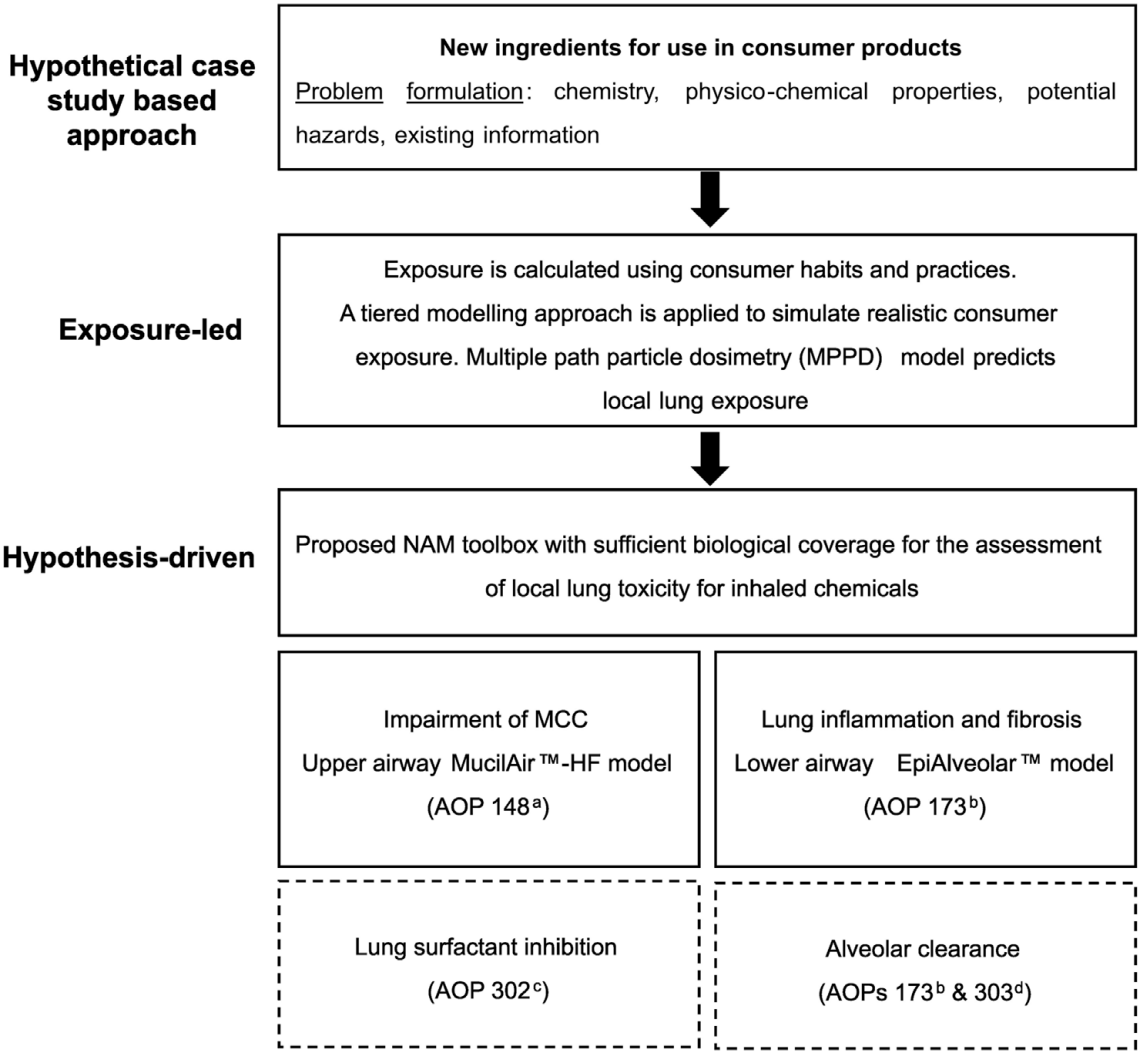
Human-relevant strategy for inhalation safety assessment recommended within next-generation risk assessment (NGRA). The strategy is structured around the context of use (i.e. product formats and duration of application), exposure to the respiratory tract (regional lung deposition), the types of chemicals of interest (e.g., polymers, preservatives) and the associated potential adverse effects. The two main product formats are pressurised propellant-driven aerosols (e.g., antiperspirants and hairsprays) and pump/trigger sprays (e.g., hair and cleaning products). These two product formats produce different particle/droplet size distribution patterns. The predicted regional lung deposition using the multiple path particle dosimetry (MPPD) will differ between these two formats, with pressurised propellant-driven aerosols leading to alveolar (particles < 7 µm), tracheobronchial and head (nasal and pharynx) exposures, whereas pump/trigger sprays mostly lead to distribution in tracheobronchial and head regions. The selection of new approach methodologies (NAMs) to assess inhalation toxicity was informed by this extensive knowledge of product use and human exposure. The establishment of a comprehensive NAM toolbox relied on adverse outcome pathways (AOPs) associated with inhalation toxicity. Several adverse outcomes stem from a common molecular initiating event (MIE), which is the interaction of chemicals with lung cells, and involve similar intermediate key events (KEs). Based on this, pulmonary inflammation/fibrosis, impairment of mucociliary clearance (MCC), lung surfactant inhibition, and alveolar clearance were prioritized as the key endpoints of concern. This study focuses on evaluating the NAM toolbox for only two of these endpoints - impairment of MCC and pulmonary inflammation/fibrosis using upper (MucilAir™-HF) and lower (EpiAveolar™) respiratory tract models. ^a^ https://aopwiki.org/aops/148 ^b^ https://aopwiki.org/aops/173/OECD Series on AOP No. 33 (Halappanavar et al., 2023) ^c^ https://aopwiki.org/aops/302 ^d^ https://aopwiki.org/aops/303.

Inspired by the NGRA concepts applied to other safety areas, such as systemic toxicity (Middleton et al., 2022), skin sensitisation (Gilmour et al., 2020; Gilmour et al., 2022), and development and reproductive toxicity (DART) (Rajagopal et al., 2022), we developed a strategy for inhalation safety assessment of consumer goods which considered the context of use (i.e. product formats and duration of application), the exposure of the ingredients to the respiratory tract (regional lung deposition/dosimetry), the types of ingredients of interest (e.g., polymers, preservatives) and the associated potential human adverse effects (e.g., lung inflammation and fibrosis) (Figure 1). The two main product formats are pressurised propellant-driven aerosols (e.g., antiperspirants and hairsprays) and pump/trigger sprays (e.g., hair and household cleaning products) which may be used daily through multiple applications. These two product formats produce different particle/droplet size distribution patterns. For example, pressurised propellant-driven aerosol hairsprays generally produce droplets with mass median aerodynamic diameters (MMAD) in the range of 2–7 μm, whereas hair pump sprays generate MMADs in the region of 5–15 µm. Given this difference, it is important to understand the realistic human exposure scenarios and then estimate the respiratory tissue dosimetry of materials potentially inhaled from these products. The predicted regional lung deposition using computational models, such as the multiple path particle dosimetry (MPPD) (https://www.ara.com/mppd/), can differ between these two formats, with pressurised propellant-driven aerosols leading to alveolar (particles < 7 µm), tracheobronchial and head (nasal and pharynx) exposures, whereas pump/trigger sprays mostly lead to distribution in tracheobronchial and head regions.

The current mechanistic understanding behind chemically-induced respiratory adverse effects through adverse outcome pathways (AOPs) can facilitate the establishment of appropriate NAM-based toolboxes with broad coverage of bioactivity readouts/biomarkers relevant to inhalation hazards which can provide *in vitro* point of departures (PoDs). In turn, *in vitro* PoDs and exposure estimates can be combined into a single metric to understand safe levels in humans, i.e., the bioactivity exposure ratio (BER), also known as margin of safety or margin of exposure (Health Canada, 2021; Middleton et al., 2022; Paul Friedman et al., 2019; Reardon et al., 2023; Wetmore et al., 2015).

In this pilot work, we aimed to investigate the feasibility of defining an NAM toolbox for lung toxicity assessment using two commercial 3D reconstructed human lung models to represent the upper and lower respiratory tract, namely MucilAir™-HF and EpiAlveolar™ systems, respectively. The different bioactivity readouts (from which PoDs are derived) are mixture of readouts directly mapped into the AOPs relevant for lung toxicity (specific) and non-specific bioactivity. For example, specific lung biomarkers such as cilia beating frequency or mucin secretion were selected, in comparison to general markers of cell integrity and transcriptomics. Both are intended to be used in a protective manner, i.e. they represent a measure of bioactivity that can be used in an exposure-led safety assessment (Cable et al., 2024). To investigate the feasibility of these assays to provide protective PoDs and BER estimates, a panel of benchmark chemicals, selected based on historical safety decisions and covering several human exposure scenarios (e.g., consumer goods products and occupational use scenarios), was tested.

## 2 Materials and methods

### 2.1 Human-relevant strategy for selecting NAMs for lung toxicity NGRA

The selection of NAMs for use in risk assessments for both acute and chronic lung toxicity was informed by an extensive knowledge of product use and human exposure, as previously mentioned (Figure 1). The aim was to identify assays that provide broad biological coverage of the key adverse outcomes across the upper (nose to larynx) and lower (trachea to alveoli) respiratory tract to support risk assessments that will protect the exposed human population. The establishment of a comprehensive NAM toolbox was anchored in several AOPs associated with lung toxicity (Clippinger et al., 2018a; Clippinger et al., 2018b; Halappanavar et al., 2020; Luettich et al., 2021; Luettich et al., 2017), details of which can be found on the virtual platform for the development and storage of AOPs (https://aopwiki.org/). Literature shows that several adverse outcomes can stem from a common molecular initiating event (MIE), defined as the initial interaction between a molecule and a biomolecule or biosystem that can be causally linked to an outcome via a pathway (Allen et al., 2014). This common MIE occurs at the level of the epithelial lung cells, and the AOPs also involve similar intermediate key events (KEs) (Halappanavar et al., 2020). Based on this, pulmonary inflammation/fibrosis, impairment of mucociliary clearance (MCC), lung surfactant inhibition, and alveolar clearance can be highlighted as the key endpoints of concern for inhaled materials (Figure 1). This paper focuses on our preliminary evaluation of the NAM toolbox for KEs linked to only two of these adverse effects - impairment of MCC and pulmonary fibrosis in upper and lower respiratory tract, respectively, considering that these regions are generally the most sensitive targets to chemicals in which humans may be repeatedly exposed via inhalation (Escher et al., 2010).

**FIGURE 2:**
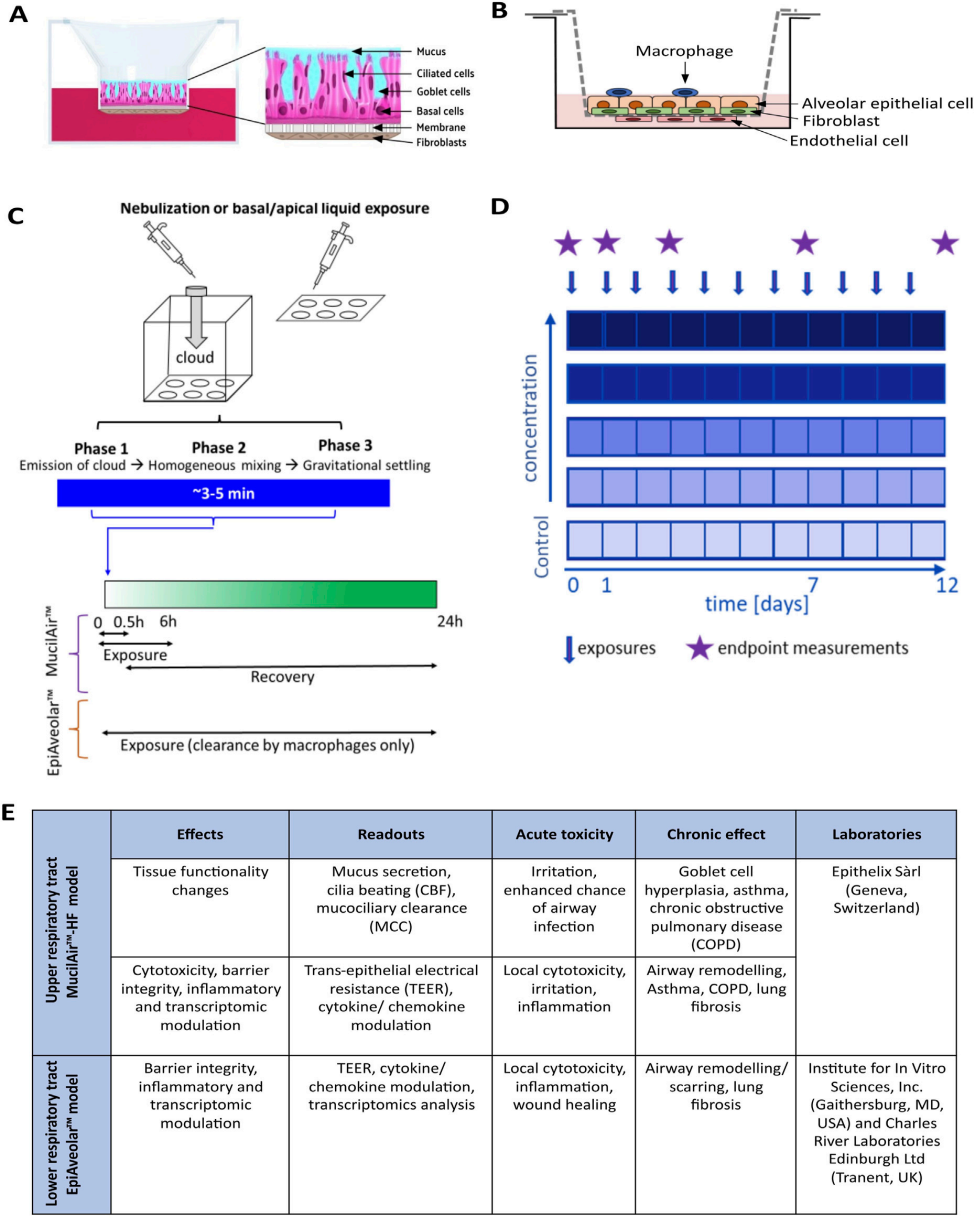
Schematic illustration of the upper MucilAir™-HF and lower EpiAveolar™ respiratory tract models, test material exposure, study design and selection of bioactivity readouts aligned to adverse outcome of pathways (AOPs)/endpoints of interest. **(A)** MucilAir™ model (Epithelix, Geneva, Switzerland) is a commercial air-liquid interface system composed of primary human cells that are differentiated into a ciliated pseudostratified respiratory epithelium with barrier function, including ciliated, basal, and goblet cells that produce mucus. **(B)** EpiAlveolar™ model (MatTek Corporation, Ashland, MA, United States) is a commercial air-liquid interface system composed of human primary alveolar epithelial cells, fibroblasts, endothelial cells, and macrophages and have shown to replicate biological responses to known pro-fibrotic compounds after sub-chronic exposures **(C)** Both models are stable for extended periods and allows for single or repeated exposure, either via liquid application or a nebulization system. The nebulization process occurs during 3–5 min through test material-containing cloud emission, homogeneous mixing, and droplets gravitational settling phases. **(D)** For toxicity testing, tissues were exposed to the test materials once a day repeatedly for 30 min/6 h (for MucilAir™) or 24 h (for EpiAlveolar™) over 12 consecutive days. **(E)** Several bioactivity readouts, that translate to substance-induced acute and chronic effects examinations, were investigated at different time points over a 12-day experimental period by three laboratories.

MCC plays a vital role in the innate immune defence against airborne pathogens and inhaled xenobiotics. The protective mucous layer, the airway surface liquid layer, and the cilia on the surface of ciliated cells are the key functional components responsible for this process. Any disturbance in the processes regulating these components can lead to MCC dysfunction. This dysfunction has been associated with the development of lung diseases such as chronic obstructive pulmonary disease (COPD) and asthma, which pose a significant risk of increased morbidity and mortality. AOP 148 (https://aopwiki.org/aops/148) outlines the mechanism by which exposure to inhaled toxicants can lead to mucus hypersecretion and subsequently affect pulmonary function (Luettich et al., 2017). Later, Luettich et al. (2021) expanded AOP 148 further to include oxidative stress as an MIE, decrease cilia beating frequency (CBF) and decrease MCC as KE6 and KE7, respectively. To obtain bioactivity associated with the MIEs or KEs outlined in both AOPs, the MucilAir™-HF model (Epithelix, Geneva, Switzerland) was selected. This system is composed of primary human cells that are differentiated into a ciliated pseudostratified respiratory epithelium with barrier function, including ciliated, basal, and goblet cells that produce mucus (Huang et al., 2011) (Figure 2A). The model has been shown to be stable for extended periods and allows for both single and repeated substance exposure, either via liquid application or nebulization system, and provides a realistic representation of the route of administration of inhaled materials, using, for instance, the Vitrocell^®^ Cloud 12 system chamber (Vitrocell Systems Gmbh, Waldkirch, Germany). Measurements of mucin secretion, CBF, MCC, and a panel of cytokines/chemokines were taken at three timepoints over a 12-day experimental period (Figures 2C–E).

Lung fibrosis is characterised by the progressive and irreversible destruction of lung architecture caused by a dysregulated tissue repair process (Wynn, 2011). AOP 173 (https://aopwiki.org/aops/173), also summarized in the OECD Series on AOP No. 33 (Halappanavar et al., 2023), describes the relationship between the initial interaction of a test material with components of the resident lung cellular membrane (considered the MIE), and the subsequent KEs triggered by the release of pro-inflammatory and pro-fibrotic mediators that signal the recruitment of immune cells into the lungs. If the chemical/stressor is not cleared effectively and/or, in particular, if there is repeated exposure, the persistent inflammation triggers to fibroblast proliferation and myofibroblast differentiation, leading to synthesis and deposition of extracellular matrix, such as collagen. In turn, excessive collagen deposition results in alveolar septa thickening, decrease in total lung volume and lung fibrosis (the adverse outcome). Pulmonary fibrosis is a complex condition that is difficult to model *in vitro* because it involves multiple cell types and typically develops over a prolonged period after repeated exposures. Nevertheless, the objective here was to evaluate a human tissue model that is intended to represent the alveolar region and that can deliver a range of readouts critical in the AOPs (Figures 2B–E). These readouts include the secretion of pro-inflammatory and pro-fibrotic mediators, loss of alveolar membrane integrity, and fibroblast/myofibroblast proliferation. When the experiments were being planned, the EpiAlveolar™ model (MatTek Corporation, Ashland, MA, United States) was deemed the most favourable commercial option. This model includes human primary alveolar epithelial cells, fibroblasts, endothelial cells, and macrophages (Figure 2B) and had shown to replicate biological responses to known pro-fibrotic compounds after sub-chronic exposures (Barosova et al., 2020). Experiments with EpiAlveolar™ model were conducted in two laboratories to compare how the results vary across different testing facilities.

Finally, a series of well-known benchmark chemicals and documented exposures that are known to be associated with or without adverse effects in humans were selected for the evaluation of the performance of each tissue model (Tables 1–3).

**TABLE 1:** Benchmark chemicals and reference materials used for the upper respiratory toxicity testing using MucilAir^TM^-HF systems.

Test materials	CAS no.	Abbreviation	Solvent	Exposure method^a^	Exposure time/day	Tested concentrations
Benchmark chemicals						
AKEMI^®^ anti-fleck super	-	Akemi	Saline	Aerosol	30 min^b^	1, 6, 12 μg/cm^2^
					6 h^c^	6 μg/cm^2^
ACUDYNE™ DHR copolymer	-	Acrylate copolymer	Saline	Aerosol	30 min^b^	0.1, 10, 100 μg/cm^2^
					6 h^c^	100 μg/cm^2^
Butyl ester of poly (methyl vinyl ether-alt-maleic acid monoethyl ester) copolymer	-	BE PVM/MA	Saline	Apical liquid	30 min^d^	0.1, 10, 100 μg/cm^2^
					6 h^e^	100 μg/cm^2^
Carboxymethylcellulose sodium salt	9004-32-4	CMC	Saline	Aerosol	30 min^b^	2.5, 5, 10, 100 μg/cm^2^
Coumarin	91-64-5	Coumarin	Saline	Aerosol	30 min^b^	0.5, 4.7, 9.4 μg/cm^2^
					6 h^c^	4.7 μg/cm^2^
Polyhexamethyleneguanidine phosphate	89697-78-9	PHMG	Saline	Aerosol	30 min^d^	0.8, 2.4, 4.8 µg/cm^2^
					6 h^c^	2.4 μg/cm^2^
Reference materials						
Nicotine	54-11-5	Nicotine	Saline	Aerosol	30 min^d^	0.04, 0.4, 4 μg/cm^2^
Lipopolysaccharide from *Pseudomonas aeruginosa* 10	-	LPS	Saline	Apical liquid	30 min^d^	0.2, 1.6, 16 μg/cm^2^
				Aerosol	6 h^c^	1.6 μg/cm^2^
Benzalkonium chloride	63449-41-2	BAC	Saline	Apical liquid	30 min^b^	0.1, 0.5, 5, 10 μg/cm^2^
Acrolein	107-02-8	Acrolein	Saline	Apical liquid	30 min^d^	500, 750, 1,000 µM
4-Chloro-3-methylphenol	59-50-7	Chlorocresol	Saline	Apical liquid	30 min^d^	1.3, 2.6, 26 μg/cm^2^
Isoproterenol hydrochloride	5984-95-2	Isoproterenol	Medium	Basal liquid	30 min^d^	1, 50, 100 µM
CFTR_inh_-172	307510-92-5	CFTR_inh_-172	DMSO	Basal liquid	30 min^d^	1, 10, 100 µM
Recombinant human TNF-α (carrier-free)	-	TNF-α	PBS + FCS	Basal liquid	30 min^d^	10, 50, 100 ng/mL
R,S-Sulforaphane	4478-93-7	Sulforaphane	Saline	Aerosol	30 min^d^	0.1, 1.4, 2.9 μg/cm^2^
					6 h^d^	1.4 μg/cm^2^

^a^ Volumes used for aerosol, apical liquid or basal liquid exposures were 0.47, 10 or 700 μL, respectively.

^b^ Male healthy donor, 41 years (batch number MD072001); age of tissue culture: 53 days.

^c^ Male healthy donor, 41 years (batch number MD072001); age of tissue culture: 81 days.

^d^ Female healthy donor, 56 years (batch number HF-MD078701); age of tissue culture: 42 days.

^e^ Male healthy donor, 41 years (batch number MD072001); age of tissue culture: 67 days.

**TABLE 2:** Benchmark chemicals and reference materials used for the lower respiratory toxicity testing using EpiAlveolar^TM^ systems.

Test materials	CAS no.	Abbreviation	Solvent	Exposure method^a^	Exposure time	Tested concentrations
Benchmark chemicals						
AKEMI^®^ Anti-Fleck Super	-	Akemi	Saline	Aerosol, daily for 12 days	24 h	0.3, 0.8, 1.6, 8 μg/cm^2^
Crystalline silica	7631-86-9	Crystalline silica	Saline	Aerosol, daily for 12 days	24 h	0.01, 1, 5, 50 μg/cm^2^
Amorphous silica	112945-52-5	Amorphous silica	Saline	Aerosol, daily for 12 days	24 h	0.01, 1, 5, 50 μg/cm^2^
Polyhexamethyleneguanidine phosphate	89697-78-9	PHMG	Saline	Aerosol, daily for 12 days	24 h	Lab 1: 0.005, 0.01, 0.05, 0.2 μg/cm^2^ Lab 2: 0.1, 0.5, 0.9, 9.4 µg/cm^2^
Amiodarone hydrochloride	19774-82-4	Amiodarone	DMSO	Basal liquid, daily for 12 days	24 h	0.01, 0.1, 1 and 10 µM
Doxorubicin hydrochloride	25316-40-9	Doxorubicin	Medium (Lab 1) or ultrapure water (Lab 2)	Basal liquid, daily for 6 days +6 days without exposure	24 h	0.18, 0.36, 0.72 µM
Reference materials						
LPS from *Pseudomonas aeruginosa* 10 (Lab 1) or *Escherichia coli* 055:B5 (Lab 2)	-	LPS	DMSO (Lab 1) or saline (Lab 2)	Apical liquid, daily for 12 days	24 h	0.01, 0.1, 1, 10 μg/mL
R,S-Sulforaphane	4478-93-7	Sulforaphane	Saline	Aerosol, daily for 12 days	24 h	0.03, 0.1, 0.6, 3 μg/cm^2^

^a^ Volumes used for aerosol, apical liquid or basal liquid exposures were 200–250 μL, 75 µL or 5 mL, respectively.

**TABLE 3:** Exposure scenarios, risk classification and associated rationale for the investigated benchmark chemicals.

Benchmark chemical	Risk classification	Risk classification reasoning	Product	Exposure scenario		Concentration or dose	Particle size (μm)	References
BE PVM/MA^a^	Low	Used safely in cosmetic products. Exposure level supported by existent toxicological data	Hair spray^a^	10% inclusion pump spray	10 min Once a day	0.017383 mg/m^3^	8.53	Carthew et al. (2006), Burnett et al. (2011)
Coumarin^a^	Low	Used safely in cosmetic products. Exposure level supported by existent toxicological data	Anti-perspirant^a^	0.08% Inclusion Spray rate 1 g/s Breathing zone volume 1 m^3^ 2s/axillae	10 min Twice a day	0.00183 mg/m^3^	5.22	Api et al. (2020)
Acrylate copolymer	Low	Used safely in cosmetic products. Exposure level supported by existing toxicological data	Hair Spray^a^	5% Inclusion Spray rate 0.59 g/s Breathing zone volume 1 m^3^ 10s application	10 min Once a day	0.022518 mg/m^3^	3.632	CIR (2019)
Amorphous silica	Low	Used safely in cosmetic products. Exposure level supported by existent toxicological data	Anti-perspirant^a^	Inclusion 0.06% Spray rate 1 g/s Breathing zone volume 1 m^3^ 8s spray time	10 min Twice a day	0.001461 mg/m^3^	6.317	CIR (2019)
	Low	The National Institute for Occupational Safety and Health recommended exposure limit (REL)	Occupational Scenario	Worker shift	8 h Shift	6 mg/m^3^	3	Barsan (2007)
CMC	Low	Used safely in nasal sprays products	Nasal spray	Single dose 0.1% inclusion level (1.25 μg/cm^2^)	N/A	N/A	N/A	Gizurarson (2012), Ugwoke et al. (2000)
BAC	Low	Used safely in nasal sprays and ophthalmic products	Nasal spray	0.01% inclusion level (0.5 μg/cm^2^)	N/A	N/A	N/A	Johnson (2017)
	Low	Used safely in homecare products. Exposure level supported by existent toxicological data	Cleaning spray	Inclusion level 0.75%	10 min Once a day	0.0081 mg/m^3^	7	Johnson (2017)
Crystaline silica	Low	OSHA: *Permissible exposure limit (PEL)*. The employer shall ensure that no employee is exposed to an airborne concentration of respirable Crystalline silica in excess of 0.05 mg/m^3^, calculated as an 8 h time-weighted average (TWA)	Occupational scenario (Low Risk)	Worker shift	8 h shift 5 days a week	0.05 mg/m^3^	3	US Occupational Safety and Health Administration (2013)
	High	Exposure likely to result in silicosis after cumulative exposure	Occupational scenario (High Risk)			5 mg/m^3^	3	'Mannetje et al. (2002)
PHMG	High	Evidence of serious adverse lung effects such as diffuse pulmonary fibrosis	Humidifier	From measurements taken of humidifier in low humidity environment	11 h Once a day	0.95 mg/m^3^	5.5	Park et al. (2014), Lee and Yu (2017)
Akemi	High	Acute lung toxicity characterised by coughing, tachypnoea, chest pain, fever, and shortness of breath	Tile coating product	Worker scenario	150 min Once per day	563 mg/m^3^	3	Duch et al. (2014)
Doxorubicin	High	Evidence of occurrence of interstitial lung disease in cancer patients	Therapeutic dose	Plasma conc 1.3 μM		Infusion of 60 mg/m^2^ for 40 min		Schwaiblmair et al. (2012), Nevadunsky et al. (2013), Hoshina and Takei (2021), Li et al. (2021), Barpe et al. (2010)
Amiodarone	High	Alveolar/interstitial pneumonitis with a subacute onset	Therapeutic dose	Plasma conc: 2 μM		Single dose of 400 mg via oral route		Wolkove and Baltzan (2009), Andreasen et al. (1981)

^a^ Scenarios for which SUET data was used to determine airborne concentration and particle size.

### 2.2 Test materials and exposure scenario selection

For the evaluation of the upper and lower respiratory models, two groups of test materials were selected: reference materials and benchmark chemicals (Tables 1, 2). Reference materials were selected to test whether the model was sensitive to chemicals known to cause a specific effect *in vitro* as described in the literature. Supplementary Table S1 describes in detail the supporting evidence for the 7 reference materials which can trigger lung toxicity via different mechanisms, including inflammation (e.g., LPS), oxidative stress (e.g., Acrolein), CBF (e.g., Chlorocresol), and mucus production/viscosity changes (e.g. CFTR_inh_-172). However, for the purposes of evaluating NAMs for use in safety assessment, it is also necessary to define exposure scenarios, that are associated either with no effects in humans or have been reported to cause adverse respiratory effects. This approach allows the evaluation of a set of tools not only in the context of hazard but incorporating exposure in the context of risk assessment (Middleton et al., 2022). The underpinning hypothesis of this benchmarking approach as an evaluation strategy is that the magnitude of the BER (i.e., the ratio between bioactivity expressed through *in vitro* PoD derivation and predicted human exposure) is correlated with level of risk in humans. In simple terms, for each benchmark chemical-exposure, a BER is calculated by dividing the lowest *in vitro* PoD across all bioactivity readouts by the predicted exposure (Middleton et al., 2022; Paul Friedman et al., 2019). In principle, a NAM toolbox is deemed successful if it is capable of distinguishing between low- and high-risk exposure scenarios as a function of the BER size.

The criteria used for the selection of benchmark chemicals included the following: 1) a human exposure can be defined (e.g., inclusion level of a chemical in a given product type, and how it is used); 2) existing toxicological information (animal, human, *in vitro*); and 3) evidence to support the high- or low-risk classification for each chemical-exposure scenario pair based on existing safety assessment and/or regulatory limits. For example, the Research Institute for Fragrance Material (RIFM) reviewed the safety of Coumarin and concluded it was safe to be used in antiperspirant aerosols up to 0.08% (Api et al., 2019; Api et al., 2020). Similarly, acrylate copolymers are frequently used as hair fixatives and supported by inhalation risk assessment at the inclusion level of 5% in hairspray aerosol (CIR, 2018). For high-risk exposure scenarios, we selected Crystalline silica, a well-known particle responsible for several cases of pulmonary fibrosis developed over many years at exposure levels higher than the permissible exposure limit (PEL) of 0.05 mg/m^3^ calculated as an 8-h time-weighted average (TWA) (Steenland and Brown, 1995). Another example of a high-risk scenario is the use of the antimicrobial polyhexamethylene guanidine phosphate (PHMG) which caused serious adverse effects in humans in Korea at the inclusion level of 1.3% in humidifiers, which were also observed in animal and *in vitro* experiments (Jung et al., 2014; Kim et al., 2016; Song et al., 2022). This paper focuses primarily on exposure to inhaled materials, however, two compounds (Amiodarone and Doxorubicin) with robust human data from oral or intravenous (i.v.) administration were tested to assess the EpiAlveolar™ model’s response to known pro-fibrotic drugs. The exposure scenarios, risk classification and associated rationale are presented in detail in Table 3. For some chemicals it was possible to find more than one scenario. In the case of Crystalline silica, different risk classifications were identified, one low risk and one high risk (see Table 3).

### 2.3 Local lung exposure estimation

#### 2.3.1 Exposure modelling

For most of the exposure scenarios related to inhaled materials (see Table 3), the objective was to obtain a worst-case human estimate of deposited aerosol concentrations in each region of the upper and lower respiratory tract (µg/cm^2^). This single value of exposure was then compared to the *in vitro* PoDs obtained from each readout of the correspondent experiment using MucilAir™-HF and EpiAlveolar™ for upper and lower respiratory tract, respectively.

Several steps were needed to predict these exposure values. The first step was to collate information about the use scenario (consumer, patient, or worker), product type, benchmark chemical inclusion level, duration of exposure, route of exposure, and particle size distribution (Table 3; Supplementary Material S1). For Amiodarone and Doxorubicin, which are administered via the oral and i.v. routes, respectively, plasma levels corresponding to a typical therapeutic dose scheme were obtained from the literature (Andreasen et al., 1981; Barpe et al., 2010). For these compounds, PoDs were expressed in µM given that this would be the most relevant metric for the risk assessment.

For the inhalation exposures, the second step was to calculate an airborne concentration which was either derived from the literature (e.g. PHMG and Akemi) or experimentally derived (Acrylate copolymer, Amorphous silica, Coumarin, BE PVM/MA, and BAC) using simulated use evaluation testing (SUET; see details below in item 2.3.3).

The third step was to use the MPPD model to calculate the deposition of each benchmark chemical in the lung based on the airborne concentration and particle size. For most exposures, particle size information was either experimentally derived (SUET data) or based on the literature (Table 3); however, for the two occupational exposure scenarios (Crystalline and Amorphous silica), a corresponding measured particle diameter was not found. In lieu, a worst-case assumption that the particle diameter in both cases was 3 μm was made, corresponding to the highest deposition fraction in the lower respiratory tract according to MPPD (version 3.04).

In the case of nasal spray based scenarios, MPPD was not used, and all exposure was assumed to be confined to the nasal cavity. Therefore, only the airborne concentration and nasal cavity areas were required to calculate a mass per unit area (Gizurarson, 2012; Johnson, 2017).

In this study, we compared a calculated local concentration in the lung for each exposure scenario to the PoD obtained in the *in vitro* lung models. For this purpose, MPPD was used to calculate the deposited mass in each lung generation. Using the mass deposited and the corresponding area of each generation, a local average concentration is determined as the ratio between the mass deposited in generation and the area of generation. This quantity is a function of the particle diameter as measured or estimated for each exposure scenario.

Once deposited, clearance mechanisms in the upper and lower respiratory tract regions begin to remove the material. These mechanisms for the clearance of material from the upper and lower respiratory tracts were modelled as described in item 2.3.2.

#### 2.3.2 *In silico* lung dosimetry modelling

MPPD (https://www.ara.com/mppd/) is a widely used tool which models human lungs as a series of interconnected pipes, i.e., it models the lungs as connected bifurcating paths, which each new bifurcation corresponding to a “lung generation”, with the trachea corresponding to generation 1 and terminating at generation 23 (terminal bronchioles) (Asgharian and Price, 2006; Asgharian and Price, 2007; Asgharian et al., 2006; Rostami, 2009; Patwa and Shah, 2015). Within each of these pipes, which vary in length and diameter according to lung generations, the deposition of a given particle/droplet is estimated based on gravitational effects, diffusion and impaction. For the purposes of this study, the lung geometry used was the symmetric Weh-Shum model with a fixed breathing rate of 12 breaths per minute, with a functional residual capacity of 3,300 mL. The upper respiratory tract volume was set at 50 mL. The tidal volume of the lung was assumed to be 625 mL with no pause between inhalation and exhalation and an inspiratory fraction of 0.5.

With these assumptions and the parameters determined for each of the exposure scenarios above (see Supplementary Material S1), the dose rate within the lung (mg/min) for each exposure scenario in each generation of the lung was calculated. In absence of any clearance, the local concentration would simply be the local dose-rate multiplied by the total exposure duration as specified in each scenario.

To make the exposure scenarios realistic, we modelled the clearance for both the upper and lower respiratory tract using the human respiratory clearance model as developed by the International Congress on Radiological Protection (ICRP, 1994), as described in Supplementary Material S2. The methodology here therefore combines the mechanistic MPPD model for predicting exposure, with the semi-empirical ICRP model for clearance. Hereafter, the ICRP model, when mentioned here, only refers to clearance modelling, as ICRP model for deposition was not used in this study. Such a hybridised approach is already taken in the commercially available MPPD software but does not return a local concentration as we desired here. The value obtained using the commercially available form of MPPD only produces a total retained mass in the upper and lower lung and so does not give the additional granularity we seek to compare *in vitro* to *in vivo* dose. However, we have used the default clearance parameters implemented in MPPD and so when compared for total mass retained our results correspond to the value obtained using the commercial software.

**FIGURE 3:**
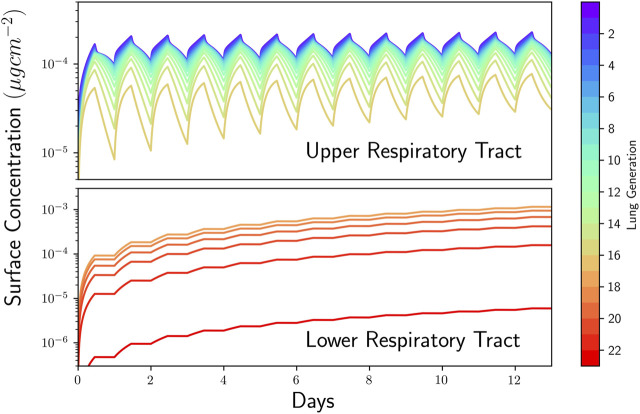
Lung dosimetry modelling using multiple path particle dosimetry (MPPD). Concentration in each generation of the lung as a function of time for Polyhexamethyleneguanidine phosphate (PHMG) humidifier exposure. Upper airway concentration (top) and lower airway concentrations (bottom) shown separately. Lung generations (i.e., tracheobronchial tree, a system partitioned into 23 generations of dichotomous branching, from trachea to the last order of terminal bronchioles, respectively, generations 0 and 23) are indicated by numbers as shown in the colour bar.

In this study, to determine the local dose within each generation of the lung, the ICRP clearance model was implemented to predict a retained mass in each lung generation over time. The average local concentration is determined by dividing the mass retained in the lung generation by the area of the corresponding generation of the lung for each given day. An example of the local concentration as a function of time is shown in Figure 3. The exposure used to compare to the *in vitro* dose response is taken to be the highest predicted exposure for each region of the lung on a measured day. By combining *in silico* deposition and clearance, this method offers a better approximation of the *in vivo* concentration for each scenario, while remaining conservative.

#### 2.3.3 Simulated use evaluation testing (SUET)

SUET is a measurement method for sprayed particles/droplets released during simulated consumer use scenarios of products, allowing the generation of realistic consumer exposure data for use in safety assessments (Carthew et al., 2002; Steiling et al., 2014). In brief, a mannequin, placed in a room of a standardised size, was positioned (e.g., arms up for axilla spraying) and equipped accordingly (e.g., wearing a full length, real hair wig for hair products), and was exposed in a realistic manner (i.e., equivalent to the expected use scenario based on the product type and user under investigation) to the sprayed product (e.g., a short burst spray at each axilla for antiperspirants). The number/mass of any airborne particles/droplets in the mannequin’s “breathing zone” during product use was determined by sampling the room air for a short duration (i.e., similar to the expected time the user remains in the vicinity of the spray cloud), via a short tube (representing the upper respiratory tract) attached to the “mouth” of the mannequin, using a TSI Model 3,321 Aerodynamic Particle Sizer time-of-flight spectrometer (TSI Incorporated, Shoreview, MN, United States) (FEA, 2009). These data were used to calculate the particle size (µm) and estimate the inhalable dose (mg/m^3^; fraction potentially depositing anywhere within the respiratory tract) during the sampling period, and then used as input for the MPPD modelling.

### 2.4 Materials

All reagents used are listed in Supplementary Material S2.

### 2.5 Upper respiratory toxicity assessment using MucilAir™-HF model

#### 2.5.1 MucilAir™-HF culture

The MucilAir™-HF system (EP11MD) and ready-to-use chemically-defined, serum-free culture medium (EP04AM) were obtained from Epithelix Sàrl (Geneva, Switzerland). Tissues were handled as recommended by manufacturer’s instruction. Epithelia (MucilAir™-HF) were used containing bronchial cells isolated from 2 healthy donors (male, 41 years, batch number MD072001; and female, 56 years, batch number HF-MD078701). After differentiation for 42 up to 81 days at the air-liquid interface (ALI) under standard culture conditions (37°C ± 1°C in a humidified atmosphere of 5% ± 1% CO_2_ in air), tissues were washed 3 days before first exposure and each insert was inspected for beating cilia and mucus production. This washing step (one or twice with PBS) were performed to remove accumulated mucus and cell debris to minimize the risk of interference with the toxicity tests. Over 12-day experimental period, washing steps as well as media changes was performed every 2-3 days. Trans-epithelial electrical resistance (TEER) was measured and only inserts which passed morphological inspection and showed TEER>200 Ω.cm^2^ were used.

#### 2.5.2 Test material exposure

To prepare the stock solutions to perform the test material exposures, buffered saline solution (0.9% NaCl, 10 mM HEPES, 1.25 mM CaCl_2_), DMSO or culture medium (Table 1). See Supplementary Material S2 for additional details.

For toxicity testing performed over a 12-day experimental period, tissues were daily exposed to 3 different concentrations of each test material for 30 min or 6 h (for detailed information on each material see Table 1). The test material exposure time was chosen based on the average time (30 min) for which the upper airway mucociliary beating transport mechanism propels inhaled particles out of the human airways (Koparal et al., 2021), and previous experimental design in which MucilAir systems were daily exposed to inhaled drugs for 6 h over 12-day period (Balogh Sivars et al., 2017). Tissues were exposed by test materials via nebulization, liquid apical application, or liquid basal application. These differences in the exposure type were due to solubility of the test material, route of exposure (inhaled or systemic), or comparison with previous publications. A nebulized exposure was performed using a Vitrocell^®^ Cloud 12 system chamber equipped with a quartz crystal microbalance (QCM) (VitroCell, Germany). A 10-µm size nebulizer was used for the exposures. Untreated/vehicles (i.e. tissues exposed with medium only or vehicles used to solubilize/nebulize each material) and positive control groups were used to check the performance of the systems by evaluating TEER, CBF, mucin secretion and Mucin 5AC detection. The following agents were used as positive controls: cytomix (known inflammatory-inducing agent, prepared using 500 ng/mL TNF-α, 0.2 mg/mL lipopolysaccharide, 1% FCS, tissue exposure via liquid basal), Triton X-100 (cytotoxic at 10%, via liquid basal exposure) or IL-13 (a goblet cell hyper-, metaplasia agent at 10 ng/mL, via liquid basal exposure). For CBF analysis, tissues kept at 4°C for 1 h were also used as positive control groups since low temperatures inhibit CBF activity (data not shown).

#### 2.5.3 TEER analysis

TEER measurements were performed daily after test item exposure to evaluate tissue barrier function. Tissues were washed, 200 µL of buffered saline solution was added to the apical compartment of MucilAir™-HF cultures, and resistance was measured using an EVOMX volt-ohmmeter with chopstick style probes (World Precision Instruments, Stevenage, UK). Resistance values (Ohm, Ω) were converted to TEER (Ω.cm^2^) using the following formula, where 100 Ω is the resistance of the membrane and 0.33 cm^2^ is the total surface of the epithelium: $TEER=\left(resistance\;value\left(\Omega \right)-100\left(\Omega \right)\right)\times 0.33$ .

#### 2.5.4 CBF analysis

CBF was analysed using a system consisting of a high-speed acquisition camera (Sony XCD V60, Tokyo, Japan) connected to an Olympus BX51 microscope with a ×5 objective (Tokyo, Japan) and a specific software package (Sony ZCL Viewer, Tokyo, Japan), as previously described (Huang et al., 2017). CBF was calculated using an Epithelix software (Cilia-X) through analysis of 256 images/tissue (recording of an area corresponding to 1/10 of the total surface), captured at high frequency rate (125 frames/s) at room temperature, and expressed in Hertz (Hz).

#### 2.5.5 MCC analysis

MCC was monitored using a high-speed acquisition camera (Sony XCD-U100CR, Tokyo, Japan) connected to a microscope with a with a ×5 objective (Olympus BX51), as previously described (Huang et al., 2017).

#### 2.5.6 Mucin secretion measurement

Mucin production was quantified using an enzyme-linked lectin assay, following a protocol previously published (Rossner et al., 2019).

#### 2.5.7 Mucin-5AC detection analysis

The presence of goblet cells was assessed through immunohistochemistry analysis for mucin-5AC protein (Muc5AC) detection. IL-13 and cytomix were used as positive controls (see item 2.5.2 for additional details). On last day of experiment (day 12), cultures (n = 3 tissues/group) were rinsed in PBS and fixed by immersion in 4% formaldehyde for 20 min. Fixed tissues were embedded into paraffin, sectioned, and processed for staining on paraffin sections. The immunostaining of the sections was performed with the Benchmark automated platform (Ventana-Roche, Hoffmann-La Roche Ltd, Basel, Switzerland) and the Autostainer Link 48 (Agilent, Santa Clara, CA. United States) with the detection kit Ultraview DAB (DAB chromogeny, Hoffmann-La Roche Ltd). Sections were pre-treated using heat mediated antigen retrieval with sodium citrate buffer, pH 6, for 20 min. The section was then incubated with recombinant anti-mucin 5AC antibody (ab3649, Abcam) for 1 h at room temperature, followed by biotinylated secondary antibody (Dako) and HRP detection (HRP conjugated ABC system, Vector Laboratories), according to manufacturer’s instruction. The section was then counterstained with hematoxylin (Sigma-Aldrich) and mounted with DPX, a synthetic non-aqueous mounting medium for microscopy. Digital images of the slides were then acquired, and quantitative image analysis performed using Image Pro Plus 6.2 (Media Cybernetics) to quantify the goblet cells on a section. The whole images of stained sections were scanned, and an average 15 images/section were analysed. The results are the ratio between the mucin 5AC stained area and the total surface area of the epithelium on the section.

#### 2.5.8 Cytokine and chemokine measurements

Basolateral medium samples of the tissue cultures were collected and stored at −80°C until analysis. The following cytokines/chemokines were quantified using customized Human Luminex^®^ Discovery Assay kits (R&D Systems): chemokine (C-C motif) ligand (CCL) 2, CCL7, CCL26, C-X-C motif chemokine ligand (CXCL) 10, CXCL11, intercellular adhesion molecule-1 (ICAM-1), IL-1α, IL-6, IL-8, matrix metalloproteinase (MMP)-1, MMP-2, MMP-3, MMP-7, MMP-9, osteopontin, interferon (IFN)-γ, IL-1 receptor antagonist (IL-1Ra), urokinase-type plasminogen activator receptor (uPAR), tumour necrosis factor (TNF)-α, urokinase (uPA), serpin E1, tissue inhibitor of metalloproteinase 1 (TIMP-1), and TGF-β1. Samples were measured in technical triplicates according to the manufacturer’s recommendation using a Luminex Bio-Plex^®^ 200 RUO System (R&D Systems).

### 2.6 Lower respiratory tract toxicity assessment using EpiAlveolar™ model

The histology/immunohistochemistry, TEER, cytokine/chemokine measurements involving EpiAlveolar™ model were performed by testing facilities located in different geographies: the Institute for *In Vitro* Sciences, Inc. (Gaithersburg, MD, United States) (Laboratory 1) and Charles River Laboratories Edinburgh Ltd (Tranent, UK) (Laboratory 2). In cytokine/chemokine measurements, apical culture media samples of the tissues collected by Laboratory 2 were sent to our laboratory facility (Unilever SERS, Sharnbrook, UK), where the analyses were performed. Oxidative stress and Mitotracker staining assays were performed by Laboratory 1 only, whereas tissues material exposure for high-throughput transcriptomics analysis was performed by Laboratory 2. In this case, Laboratory 2 sent RNA samples to another laboratory facility (Cambridge Genomics Services, Cambridge, UK) to proceed with additional steps of RNA sequencing; the differential expression and pathway analyses were then performed by Unilever SERS. The laboratory work was performed independently by the laboratories, i.e., each facility used its own *in house* implemented protocols to perform test material exposure and investigate material-induced bioactivity. Additional details of all procedures are described in the next sections.

#### 2.6.1 EpiAlveolar™ culture

EpiAlveolar™ system was obtained from MatTek Corporation (Ashland, MA, United States, cat. no. ALV-100-FT-MAC, ALV-100-FT-MAC-PE12). According to the supplier, the tissue systems were derived from primary human alveolar epithelial cells and primary pulmonary fibroblasts, both from a same healthy donor (male, 50 years), and pulmonary endothelial cells from another healthy donor (male, 6 years), and THP-1 cell line derived macrophages. These tissues were differentiated by the manufacturer prior to shipping to the testing facilities. Upon receipt, systems were maintained, for 2–7 days prior to use, at the ALI in modified 6-well hanging top plates with 5 mL of EpiAlveolar™ culture medium (MatTek) in the basolateral compartment and 75 µL of media on the apical surface and incubated at standard culture conditions (37°C ± 1°C in a humidified atmosphere of 5% CO_2_ in air). TEER was measured and only systems with confirmed quality of the tight junction barrier (>300 Ω.cm^2^) and with approved morphological inspection were used in the experiments.

#### 2.6.2 Test material exposure

To prepare the stock solutions/suspensions to perform the test material exposures, saline, medium, ultrapure water or DMSO were used to dilute the test materials (Table 2). See Supplementary Material S2 for additional details.

Tissues were exposed to eight test items, including known fibrotic, inflammation inducing agents (e.g., PHMG and Doxorubicin). Vehicle control groups were also tested in parallel. Except for Doxorubicin (which underwent a 6-day chemical exposure + 6-day without exposure), exposures were conducted daily, on 12 consecutive days with different concentrations, through aerosol, apical or basolateral liquid exposure methods (Table 2). In the aerosol exposure method, the procedure was performed using a Vitrocell^®^ Cloud 12 system chamber equipped with a QCM. A 10-µm size nebulizer was used for the exposures. Each of the insert holders of the instrument base module was filled with Hanks’ Balanced Salt solution (HBSS) or PBS prior to placing the tissue inserts. Then, each test item solution/suspension was placed into the nebulizer reservoir for aerosol material exposure. The nebulizer was activated until the material solution/suspension was consumed and discharged into the main exposure chamber and allowed to fully gravity deposit out of the Cloud 12 (determined using a QCM). After exposure, the tissues were placed back into their multi-well culture plates containing the same medium, a further aliquot of media was added apically (20 µL for Akemi, 75 µL for all others except LPS) and returned to incubator until next exposure. Media used during the 6- or 12-day experimental period was prepared without Supplement X by MatTek. In medium exposure, the apical or basolateral liquid of each tissue was removed and replaced with each test item medium solution/suspension and returned to the incubator until the next exposure. After the beginning of the exposure cycles, tissues were only re-fed with new culture medium every 3-4 days.

#### 2.6.3 Histology and immunohistochemistry analyses

To evaluate the quality of EpiAlveolar™ tissues over the 12-day experimental period, histological and immunohistochemistry assessments for detection of pan-cytokeratin, vimentin, aquaporin 5, pro-surfactant C, CD68, caspase-3, and/or αSMA were performed by Laboratories 1 and 2. Details can be found in Supplementary Material S2.

#### 2.6.4 TEER analysis

##### 2.6.4.1 Laboratory 1

Tissue inserts were removed and placed into a 12-well plate containing 0.75 mL of HBSS well and 0.25 mL of HBSS were added into each apical compartment of the culture inserts. Resistance was then measured using an EVOM volt-ohm-meter with chopstick style probes (World Precision Instruments, Stevenage, UK). Resistance values (Ohm, Ω) were converted to TEER (Ω.cm^2^) using the following formula, where 1.12 cm^2^ is the total surface area of the tissue inserts: $TEER=\left(resistance\;value-tissue\;free\;membranes\;resistance\;value\right)\times 1.12$ . The change in barrier function (ΔTEER) was then calculated using the time point specific reading (day 1, 4, 8 or 12) subtracted from the initial reference reading (day 0).

##### 2.6.4.2 Laboratory 2

TEER was measured using the Millicell Electrical Resistance System-2 meter with an Endohm™ 12 Tissue Resistance Measurement Chamber electrode (Merck, Darmstadt, Germany). Tissue inserts were removed and placed into a 12-well plate containing TEER buffer (1 mL/well, MatTek) and the top surface of each tissue was gently rinsed with 0.5 mL of the same buffer. The units were then emptied and added sequentially to the measurement chamber containing 4 mL of TEER buffer. After adding 0.75 mL of TEER buffer in each tissue apical compartment, electrodes were submerged to measure the resistance. TEER values (Ω.cm^2^) were calculated as described above by Laboratory 1.

#### 2.6.5 Cytokine and chemokine measurements

In Laboratory 1, basal media samples collected from the tissues were collected and stored at ≤ −60°C until analysis. For cytokine/chemokine measurements, 17-Plex, duplex and single-plex analyte detection panels were run on the samples using Human Luminex™ Multiplex Immunoassay kits (R&D Systems). The analytes quantified were TNF-α, IL-6, MMP-9, MMP-3, MMP-1, MMP-2, CXCL10, CCL2, IFN-γ, IL-1ra, CCL7, IL-1a, CCL26, CXCL9, VCAM-1, ICAM-1, and CXCL11 in a 17-plex assay; whereas IL-8 and Serpin E1 in a duplex assay and TGF-β1 in a single-plex assay.

In Laboratory 2, apical culture media samples of the tissues were collected, stored at −80°C until shipment, in dry ice with temperature monitor control, to Unilever SERS, where the analyses were performed. Upon receipt, samples were again stored in the −80°C until the analysis using Human Luminex^®^ Discovery Assay kits (R&D Systems). The same readouts investigated for MucilAir™-HF tissue samples were quantified in these samples.

#### 2.6.6 Oxidative stress assay

Reduced glutathione (GSH) and oxidized glutathione (GSSG) levels in tissue lysate samples were determined using a GSH/GSSG-Glo™ assay kit (Promega) by Laboratory 1, as previously described (Vivek et al., 2023).

#### 2.6.7 Mitotracker staining assay

Mitochondrial toxicity assessment was performed by the Laboratory 1 using MitoTracker^®^ Red FM reagent, a cell-permeant dye able to stain active mitochondria in live cells. Tissues were loaded on the apical surface with MitoTracker^®^ Red FM dye solution (500 Nm in HBSS) for 30 min in empty 12-well plates. Afterwards, the dye solution was removed from each tissue followed by rinsing the tissue apical surfaces with 200 µL HBSS. Fluorescence reads at an excitation and emission wavelength of 581/644 nm, respectively, were performed using FlexStation^®^ 3 microplate reader. Empty inserts were utilized as a negative control to subtract the background noise or fluorescence of the dye.

#### 2.6.8 High-throughput transcriptomics analysis

##### 2.6.8.1 RNA extraction, assessment of quality, library construction and sequencing

Following material exposure for 12 days, tissues were cut from the plastic unit, transferred into 2 mL Precellys tubes (Bertin Technologies, Montigny-le-Bretonneux, France) containing 700 µL of QIAzol lysis reagent and stored at −80°C until RNA extraction. For this, samples were homogenized followed by centrifugation at 12000 g at 4°C for 5 min. The supernatants were used to extract RNA through a combined semi-automated method using a Quiagen Rneasy 96 QIAcube HT kit. All samples were DNase treated using a Thermo Fisher DNA-free kit, following manufacturer’s instructions. All these steps were performed by Laboratory 2 which, then, stored RNA samples (n = 5/group) in 96-well plates until shipment on dry ice with a temperature monitor to the RNA sequencing laboratory facility (Cambridge Genomics Services). Samples then underwent quality control (QC) using Agilent RNA ScreenTape assay, following manufacturer`s instructions, to generate RNA integrity number (RIN) score and traces for the samples. RNA library construction was performed using Illumina TruSeq™ Stranded mRNA kit, following manufacturer’s protocol. RNA-seq data mapping was processed using Spliced Transcripts Alignment to a Reference (STAR) method, as previously described (Dobin et al., 2013).

##### 2.6.8.2 Differential expression and pathway-level data extraction

The raw data for the EpiAlveolar™ experiments underwent QC. Probes whose median counts across treatment and timepoint was less than 5 were removed. All the samples had more than 5 M reads, thus no sample was removed from the analysis. No outlier samples were detected as all the replicates had a high degree of correlation (>0.9). A total of 64 samples were removed due to low quality, this was a particular issue for PHMG when the following concentrations were removed: 2nd highest concentration (0.5 μg/cm^2^) on day 12, and the two highest concentrations (0.9 and 9.4 μg/cm^2^) across all timepoints (Supplementary Material S3). Datasets were then normalized and transformed using the *rlog ()* function in DESeq2 (Love et al., 2014), to minimize differences between samples for rows with small counts and to normalize with respect to library size.

With the aim of identifying patterns of co-regulated genes in a way that is neither fully data-driven nor fully constrained by biological knowledge, Pathway-level information extractor (PLIER) (Mao et al., 2019) was used. PLIER approximates the expression pattern of every gene as a linear combination of eigengene-like latent variables (LVs) and aims to optimize alignment of LVs to relevant biological knowledge. The compendium of prior knowledge chosen for the analysis includes the full Reactome database (v. 7.5.1) (Gillespie et al., 2021) and the Hallmark gene sets (v. 7.5.1) (Liberzon et al., 2015), as provided by The Molecular Signatures Database (MsigDB). Genes sets included in the MsigDB are annotated with official gene symbols hence, the Ensembl ID used to annotate the probes needed to be converted. This step was performed using the bitr () function from the clusterProfile R package (Guangchuang Yu et al., 2012) and led to the loss of 7.8% genes from the EpiAlveolar™ dataset. Since different Ensembl IDs could match the same official gene symbol ID, this redundancy had to be removed for PLIER to work optimally. This was achieved by keeping the gene with the highest median expression. Reliable associations between LVs and gene sets as identified by PLIER were filtered for AUC > = 0.7 and fdr < 0.05. As the EpiAlveolar™ model underwent material exposure at different concentrations, the LVs expression dataset was then used as to analyse time concentration-dependent effects and to estimate PoDs for each LV for each test material at different timepoints. Data has been submitted to the European Bioinformatics Institute (EBI) data repository, Array Express (https://www.ebi.ac.uk/biostudies/arrayexpress) under the accession number E-MTAB-14272.

### 2.7 Data analysis

#### 2.7.1 Concentration and time-dependent response analysis and *in vitro* PoD determination

Concentration and time-dependent changes in the bioactivity readout data were analyzed using a nonlinear state space model, based on the work of Svensson and Schön (2017). The method was adapted to allow for experimental measurements where the same readout was observed for multiple different test material treatments and concentrations, observed at different timepoints (Middleton et al., in preparation). All bioactivity readouts were included in the analysis (where available), i.e., measurements for tissue integrity loss (TEER) and functionality (MCC, CBF, and mucin secretion), cytokine/chemokine secretion, and the transcriptional LV values obtained using PLIER. The datasets were grouped so that a single state space model was used to capture all the responses (across time, concentration, and test material) for a single readout obtained within a particular study (laboratory/tissue model). Data were transformed to the log base 10 scale and normalized using z-scores, so that for a given readout $Y_{t}^{ij}$ from a laboratory/tissue model, the corresponding normalized value was given by ${\hat{y}}_{t}^{ij}={\left(\log10(Y\right.}_{t}^{ij})-\mu )/\sigma$ . Here, the indexes i,j,t correspond to the different test materials, concentrations and timepoints, μ and σ are the sample mean and standard deviation of the control readout values obtained for the first timepoint. All measurement were then scaled so that the maximum median value across different replicates was one for all timepoints, readouts and test materials within a given laboratory/tissue model.

The model consists of state variables $x_{t}^{i,j}$ , response variables $y_{t}^{ij}$ and the concentrations of material $c^{i,j}$ . The response variables $y_{t}^{i,j}$ represent the normalized (see below) readout values at timepoint t for material exposure i,j . The mean of the response variable is given by $x_{t}^{i,j}$ , so that $y_{t}^{i,j}∼x_{t}^{i,j}+e_{t}$ , where $e_{t}$ represents random fluctuations in the readout values, due to observational noise, which are taken to be normally distributed. Dynamical changes in state values are given by $x_{t}^{i,j}=f\left(x_{t-1}^{i,j},c_{t}^{i,j}\right)+v_{t}$ . Here, $f\left(x_{t-1}^{i,j},c^{i,j}\right)$ is a non-linear function that represents interactions between different state variables and the perturbation caused by the test material at a given exposure at concentration $c^{i,j},$ and $v_{t}$ represents random fluctuations in state dynamics (which are also normally distributed). Following Svensson and Schön (2017), the nonlinear terms are based on an approximation to Gaussian processes, which are obtained using a basis function expansion-based approach. Inference of the model parameters was performed in the framework of Bayesian inference, wherein random draws of the posterior distribution were obtained using Sequential Monte Carlo (SMC), see Svensson and Schön (2017) and Chopin and Papaspiliopoulos (2020). For each state space model representing a particular laboratory/readout dataset, SMC simulation was run for three chains at 5,000 iterations each, and first 1,000 iterations were discarded from the final posterior samples. The Rhat convergence diagnostic (Rhat < 1.1) was used to ensure the simulations had converged.

**FIGURE 4:**
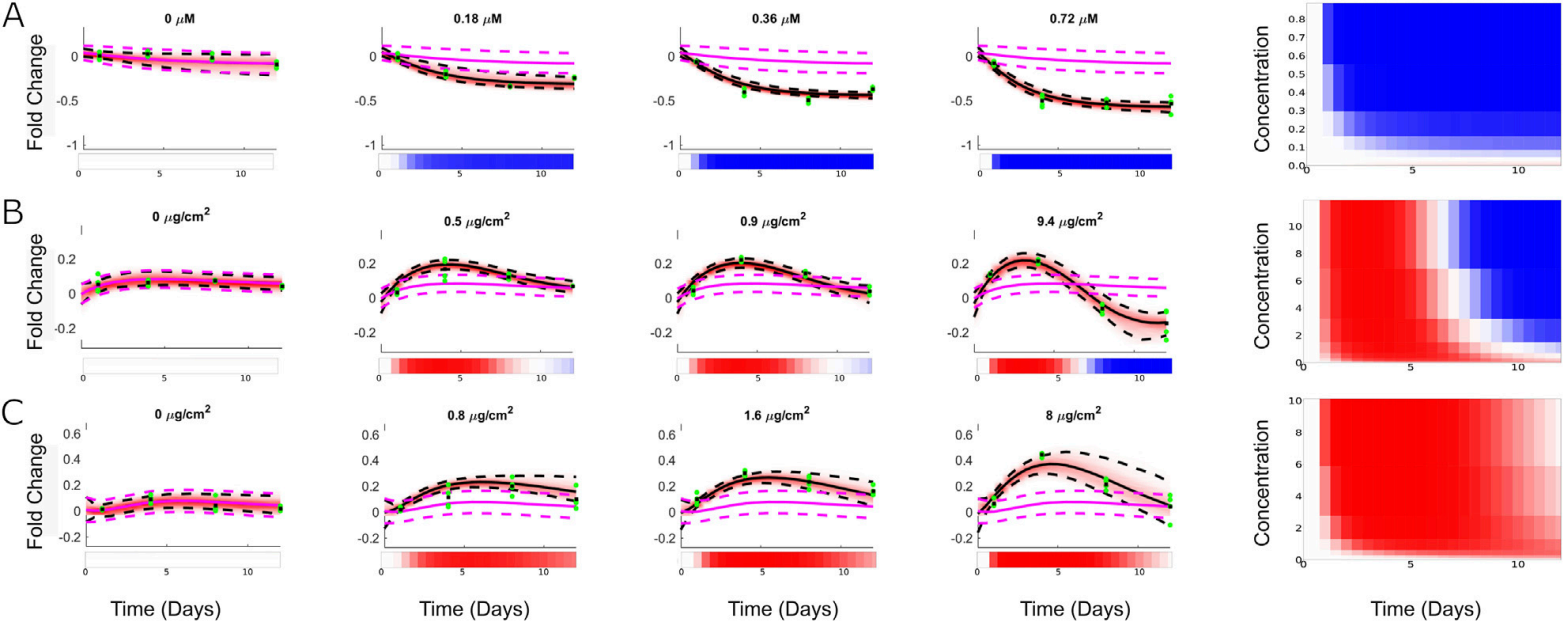
Representative concentration response data and model fits using the state space model. Left) Posterior predictive plots comparing the inferred trajectories of the different readouts in response to chemical treatment to the normalised data for **(A)** Doxorubicin for MMP -3, **(B)** PHMG for IL-1ra, **(C)** Akemi for IL-1α. Pink and black dashed lines represent 95% credible range of vehicle control and cred range of mean response, respectively. Green dots are the data points, whereas the depth of red shading reflects the probability distribution of the mean response. Blank, blue, and red colours represent no changes, down- or upregulation in relation to control (at same timepoint), respectively, whereas the colour variation shows the intensity of such effects. Right) Corresponding heat map representations of the biomarker response as described by the state space model blank, blue, and red colours represent no changes, down- or upregulation in relation to control (at same timepoint), respectively, whereas the colour variation shows the intensity of such effects.

The state space model and posterior model parameter samples were then used to construct posterior predictive distributions of the response of each readout to a given test material for a given concentration and timepoint (see Figure 4). The posterior predictive distributions were used to estimate various statistics on the readout responses (median, 95% credible range etc.), PoDs, Concentration Dependency Scores (CDS) and two Effect Scores (increase and decrease). PoDs were defined as the concentration at which the mean response to a test material exceeded the 95% credible range of control for each timepoint (otherwise the mean response remains within the range of control). PoDs distribution for each timepoint, test material and readout, from which different statistical measures (e.g., the median, 95% credible range etc.) were calculated. The CDS was designed to provide confidence metric in whether an effect in the data was truly concentration dependent. Following Hatherell et al. (2020), the CDS (for a given timepoint, test material and readout) was calculated as the proportion of posterior samples there was a PoD. CDS values could vary between 0 and 1 (0 indicating high confidence that there was no effect, 1 indicating high confidence there is an effect). A CDS>0.5 was required for an effect to be considered concentration dependent. The Effect Score was subdivided into a metric of increasing responses and decreasing responses (ES_increase and ES_decrease). These were calculated as the proportion of posterior samples where the mean response increases above (for ES_increase) or below (ES_decrease) the control range. ES_increase and ES_decrease values vary between 0 and 1 (so that the maximum value of ES_increase plus ES_decrease is 1). The ES_increase and decrease values were plotted as heatmaps to visualize the effect of the test material over time and concentration (see Figure 4).

#### 2.7.2 BER calculation

A BER is defined as the ratio between the *in vitro* PoD and predicted human exposure (Health Canada, 2021; Middleton et al., 2022). If a PoD was determined, a BER was calculated for each benchmark chemical-exposure scenario, using the lowest PoD among the different readouts (i.e. lowest across readouts from TEER, tissue functionality, cytokine/chemokine secretion and/or transcriptional LVs) investigated across the two respiratory tract models and laboratories. As such, the following BERs could be calculated per benchmark chemical-exposure scenario (see Table 3) and upper and lower respiratory tract tissue model (i.e. MucilAir™-HF and EpiAlveolar™ models, respectively):

• Ratio between the lowest PoD from all MucilAir™-HF model readouts and the highest exposure estimate in the upper respiratory tract;
• Ratio between the lowest PoD from all EpiAlveolar™ model readouts in Laboratory 1 and the highest exposure estimate in the lower respiratory tract;
• Ratio between the lowest PoD from all EpiAlveolar™ model readouts (except transcriptomics) in Laboratory 2 and the highest exposure estimate in the lower respiratory tract;
• Ratio between the lowest transcriptional PoD in the EpiAlveolar™ model in Laboratory 2 and the highest exposure estimate in the lower respiratory tract.

## 3 Results

**FIGURE 5:**
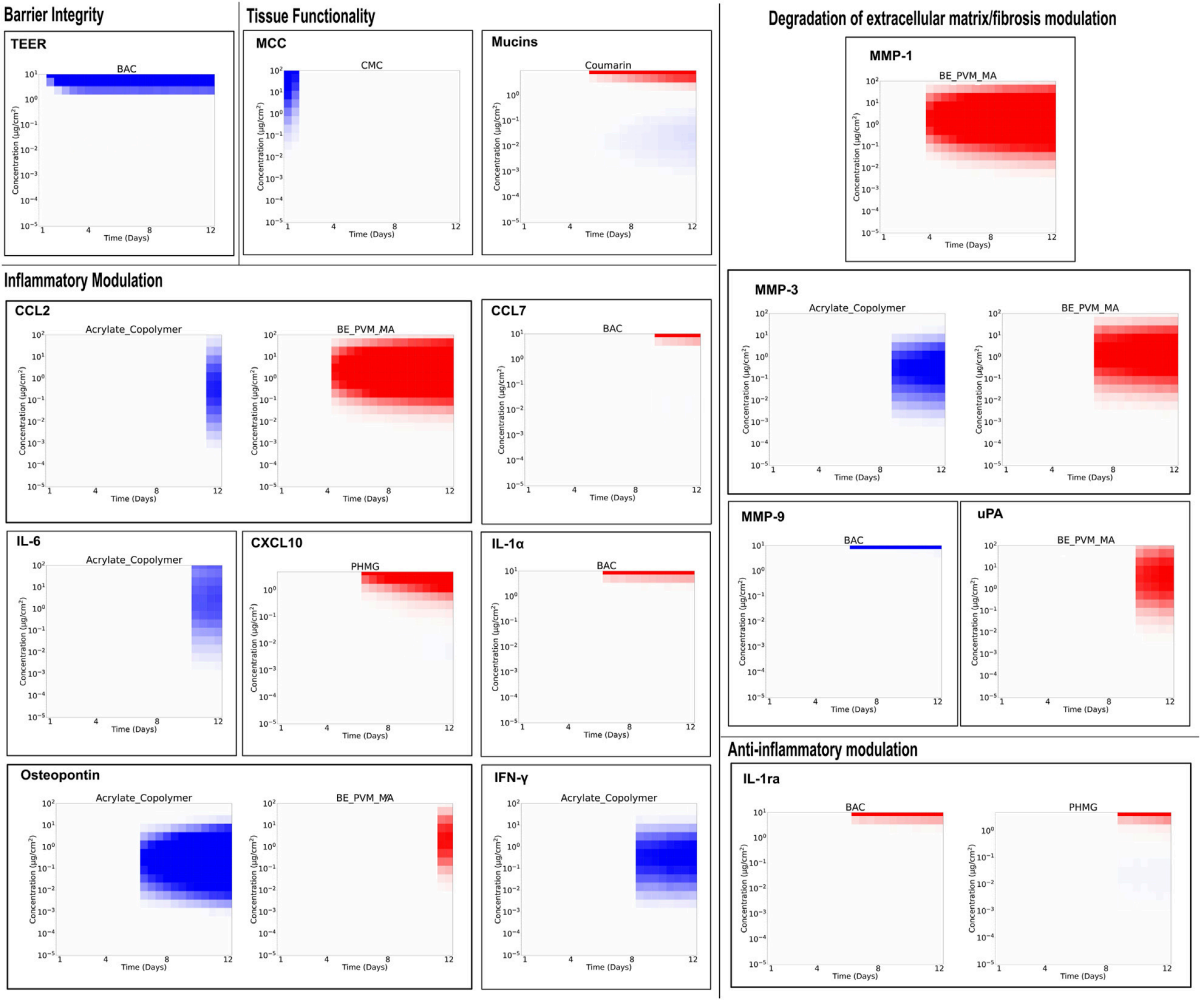
Test material-induced changes in the upper respiratory MucilAir™-HF tissues. Tissues were exposed to the materials, through aerosol (^Ar^) or via apical (^AL^)/basal liquid (^BL^) application, once a day repeatedly for 30 min/day on 12 consecutive days with 3-4 different concentrations (n = 3 tissues/concentration). On days 1, 4, 8, and 12, the following bioactivity readouts were evaluated: barrier integrity through transepithelial electrical resistance (TEER) measurements analysis; tissue functionality by the assessment of cilia beating frequency (CBF), mucociliary clearance (MCC) and mucin secretion; inflammatory, degradation of extracellular matrix/fibrosis, and anti-inflammatory responses through cytokine and chemokine quantification. Figure shows only test materials that triggered changes in the investigated readouts when compared to vehicle tissues groups. (The full data, including substances that did not trigger any bioactivity, can be found in Suppl. Data 4). Blue and red colours represent down- or upregulation, whereas the colour variation shows the intensity of such effects.

**FIGURE 6:**
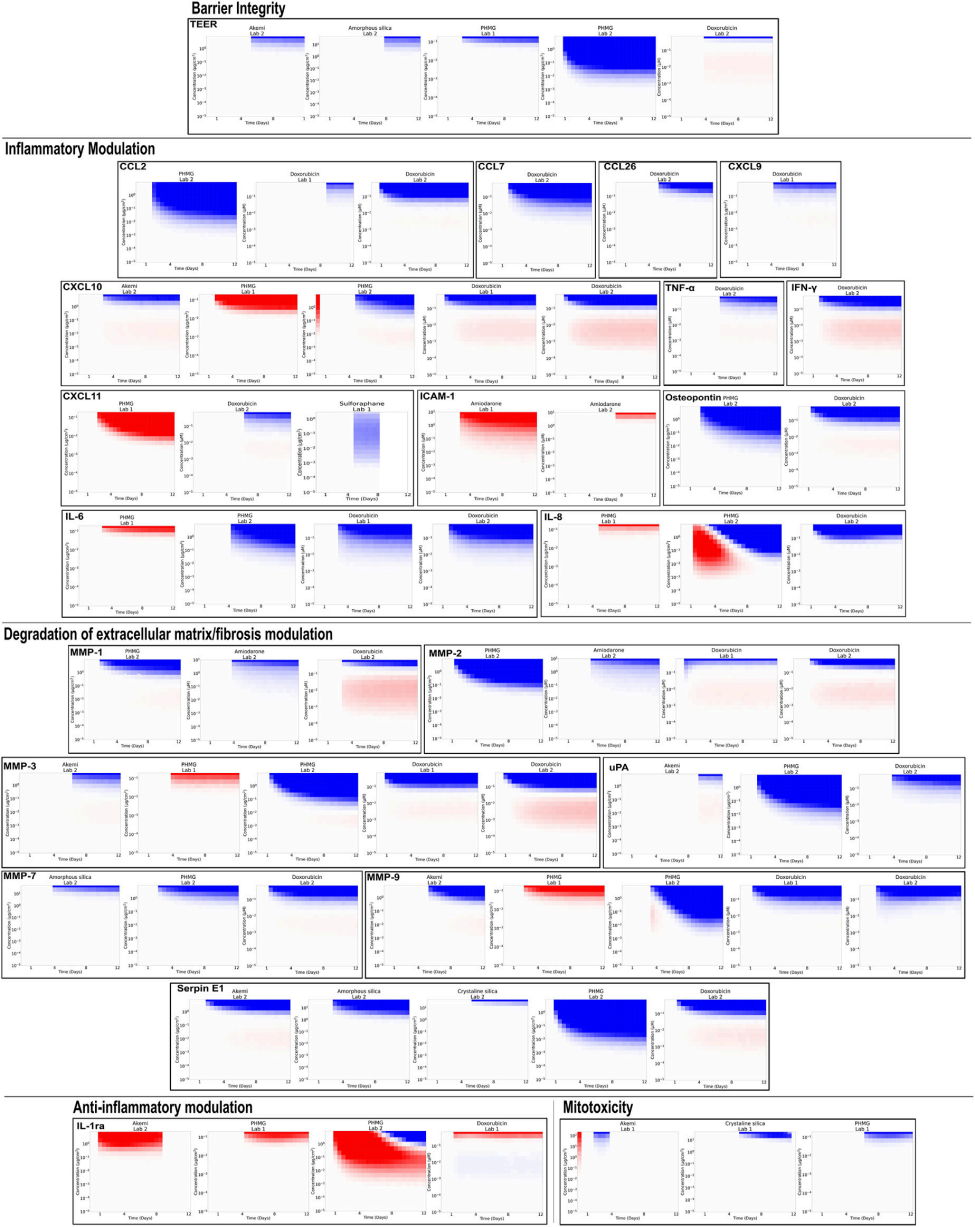
Test material-induced changes in the lower respiratory EpiAveolar™ tissues. Except for Doxorubicin (which underwent a 6-day exposure + 6-day without exposure), tissues were exposed to the materials, with 3-4 different concentrations (n = 3 tissues/concentration), were conducted on 12 consecutive days through aerosol (^Ar^), apical (^AL^) or basolateral (^BL^) liquid exposure methods. On days 1, 4, 8, and 12, basolateral culture medium samples of each tissue were collected for assessment of the following readouts: barrier integrity through transepithelial electrical resistance (TEER) measurements analysis; inflammatory, degradation of extracellular matrix/fibrosis, and anti-inflammatory responses through cytokine and chemokine quantification. Figure shows only test materials that triggered changes in the investigated readouts when compared to vehicle tissues groups. (The full data, including substances that did not trigger any bioactivity, can be found in Suppl. Data 4). Blue and red colours represent down- or upregulation, whereas the colour variation shows the intensity of such effects.

**FIGURE 7:**
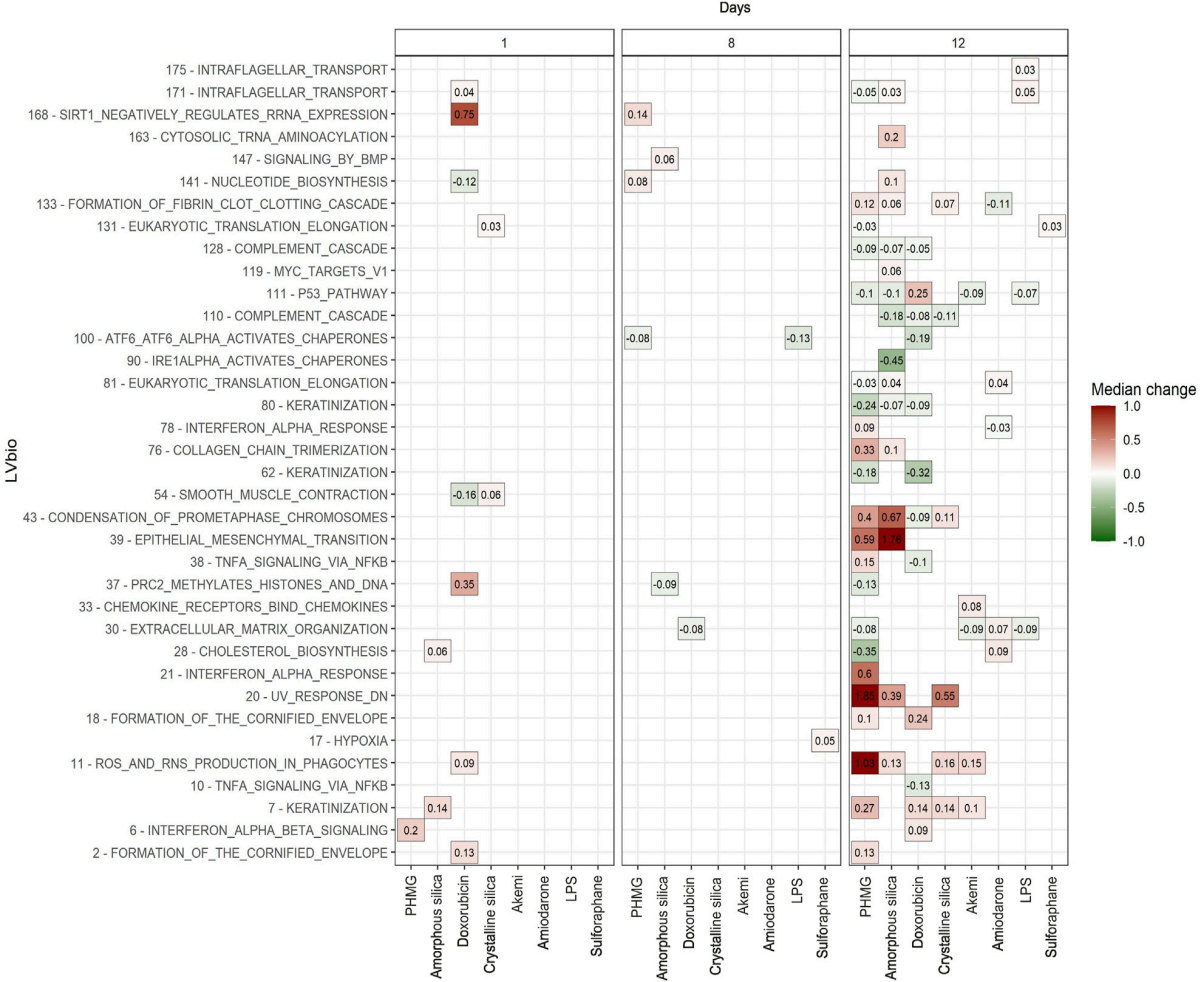
High-throughput transcriptomics of the lower respiratory EpiAveolar™ tissues exposed to benchmark chemicals. Except for Doxorubicin (which underwent a 6-day exposure + 6-day without exposure), exposures to the materials, with 3-4 different concentrations (n = 5 tissues/concentration), were conducted on 12 consecutive days through aerosol (Ar), apical (AL) or basolateral (BL) liquid exposure methods. On days 1, 4, 8, and 12, tissues were collected for transcriptomics analysis. Figure displays the latent variables (LVs) that showed significant concentration- and time-dependent response after benchmark chemical exposure. Colour-coding shows the maximum medium fold difference (between the median treated response relative to the median time-matched vehicle control value) across all test concentrations.

**FIGURE 8:**
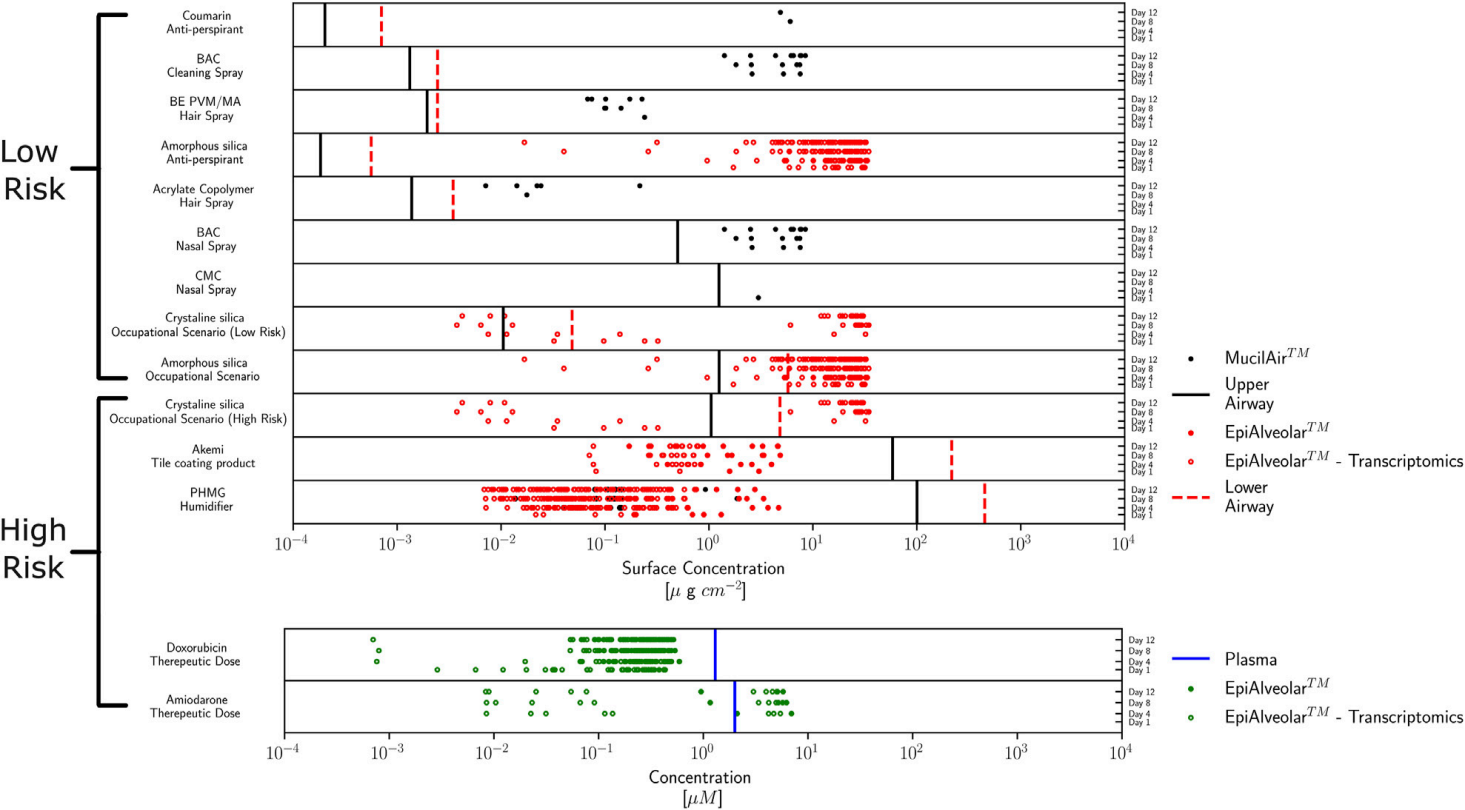
Comparison of human internal exposure (upper/lower respiratory tract or plasma) and *in vitro* point of departures (PoDs) per benchmark chemical using MucilAir™-HF or EpiAveolar™ models. All obtained PoDs, bioactivity readouts and timepoints (days 1, 4, 8 or 12) are plotted together with the associated lung regional concentration estimates (top) or maximum plasma concentration, C_max_ (bottom). The points correspond to the median PoD values from different concentration-response datasets for which the concentration dependency scores (CDS)>0.5 (where the legend indicates the corresponding tissue model). PoDs determined from transcriptomics are indicated by unfilled symbols and other readouts (e.g., barrier integrity, tissue functionality, or cytokines/chemokines levels) by filled symbols.

This section describes in detail the main results obtained when MucilAir™-HF and EpiAlveolar™ models were exposed daily to benchmark chemicals and reference materials (Tables 1, 2), in three different exposure methods (aerosol, apical and/or basal liquid), over a 12-day experimental period. Several bioactivity readouts were investigated, including TEER, tissue functionality, cytokine/chemokine secretion and/or gene expression. The effects induced by the reference materials as well as relevant literature data are also summarized in Supplementary Table S1. A comparison between the concentration- and time-dependent response analysis and corresponding readout data can be found in the Supplementary Material S4, whereas the main analyses are shown in Figures 5–8.

**TABLE 4:** Observed effects in the test material-exposed MucilAir^TM^-HF tissues over 12-day period.

Test materials^a^	Exposure time/day	Tissue barrier integrity loss	Tissue functionality				Modulation of cytokines and chemokines		
			Increased mucin secretion	Reduced CBF	Reduced MCC	Increased Muc5AC protein	Inflammatory modulation	Extracellular matrix/fibrosis modulation	Anti-inflammatory modulation
Benchmark chemicals									
Akemi^Ar^	30 min	⨯	⨯	⨯	⨯	⨯	⨯	⨯	⨯
	6 h	⨯	⨯	⨯	⨯	⨯	✔	✔	⨯
Acrylate copolymer^Ar^	30 min	⨯	⨯	⨯	⨯	⨯	✔	✔	⨯
	6 h	⨯	⨯	⨯	⨯	⨯	✔	✔	⨯
BAC^AL^	30 min	✔	⨯	⨯	⨯	⨯	✔	✔	✔
BE PVM/MA^AL^	30 min	⨯	⨯	⨯	⨯	⨯	✔	✔	⨯
	6 h	✔	⨯	✔	⨯	⨯	✔	✔	✔
CMC^Ar^	30 min	⨯	⨯	⨯	✔	⨯	⨯	⨯	⨯
Coumarin^Ar^	30 min	⨯	✔	⨯	⨯	⨯	⨯	⨯	⨯
	6 h	⨯	⨯	⨯	⨯	⨯	⨯	⨯	⨯
PHMG^Ar^	30 min	⨯	⨯	⨯	⨯	⨯	✔	⨯	✔
	6 h	⨯	✔	⨯	⨯	⨯	✔	⨯	✔
Reference materials									
Nicotine^Ar^	30 min	⨯	⨯	⨯	⨯	⨯	⨯	⨯	⨯
LPS^AL/Ar^	30 min	⨯	⨯	⨯	⨯	⨯	⨯	⨯	⨯
	6 h	⨯	⨯	⨯	⨯	⨯	✔	✔	⨯
Sulforaphane^Ar^	30 min	⨯	⨯	⨯	⨯	⨯	⨯	⨯	⨯
	6 h	⨯	⨯	⨯	⨯	⨯	⨯	⨯	⨯
Acrolein^AL^	30 min	⨯	⨯	⨯	⨯	⨯	⨯	⨯	⨯
Chlorocresol^AL^	30 min	⨯	⨯	⨯	⨯	⨯	⨯	⨯	⨯
Isoproterenol^BL^	30 min	⨯	⨯	⨯	⨯	✔	⨯	✔	⨯
CFTR_inh_-172^BL^	30 min	⨯	⨯	⨯	⨯	⨯	⨯	⨯	⨯
TNF-α^BL^	30 min	✔	⨯	⨯	⨯	⨯	✔	⨯	⨯

The symbols ⨯and ✔ absence or presence of bioactivity induced by the related chemical, respectively.

^a^ Tissues were exposed to test materials via aerosol (^Ar^), apical liquid (^AL^) and/or basal liquid (^BL^) application.

**TABLE 5:** Observed effects in test materials exposed EpiAlveolar^TM^ tissues over 12-day period and qualitative concordance between laboratories 1 and 2.

Test materials^a^	Tissue barrier integrity loss		Modulation of cytokines and chemokines						Changes in GSH and GSSS levels^b^	Mitotoxicity^b^
			Inflammatory modulation		Extracellular matrix/fibrosis modulation		Anti-inflammatory modulation			
	Lab 1	Lab 2	Lab 1	Lab 2	Lab 1	Lab 2	Lab 1	Lab 2	Lab 1	Lab 1
Benchmark chemicals										
Akemi^Ar^	⨯	✔	⨯	✔	⨯	✔	⨯	✔	⨯	✔
Crystalline silica^Ar^	⨯	⨯	⨯	⨯	⨯	✔	⨯	⨯	⨯	✔
Amorphous silica^Ar^	⨯	✔	⨯	✔	⨯	✔	⨯	⨯	⨯	⨯
PHMG^Ar^	✔	✔	✔	✔	✔	✔	✔	✔	⨯	✔
Amiodarone^BL^	⨯	⨯	✔	✔	⨯	✔	⨯	⨯	⨯	⨯
Doxorubicin^BL^	⨯	✔	✔	✔	✔	✔	✔	⨯	⨯	⨯
Reference materials										
LPS^AL^	⨯	⨯	⨯	⨯	⨯	⨯	⨯	⨯	⨯	⨯
Sulforaphane^Ar^	⨯	⨯	⨯	⨯	⨯	⨯	⨯	⨯	⨯	⨯

The symbols ⨯and ✔ absence or presence of bioactivity induced by the related chemical, respectively.

^a^ Tissues were exposed to test materials via aerosol (^Ar^), apical liquid (^AL^) and/or basal liquid (^BL^) application.

^b^ This readout was investigated by Laboratory 1 only.

### 3.1 Effects of materials in the upper respiratory tract MucilAir™-HF model

#### 3.1.1 Tissue barrier integrity loss

Table 4 summarizes the effects in the tissue barrier integrity through TEER measurement analysis when the MucilAir™-HF tissues were exposed to test items for 30 min/day and/or 6 h/day. Among the benchmark chemicals, only BAC were able to trigger changes in tissue barrier integrity at 30 min/day exposure; however, this finding was observed mainly at high tested concentrations (Table 4; Figure 5). Interestingly, no effects were observed following exposure to BE PVM/MA at 30 min/day, unlike following the 6 h exposure/day regimen (Table 4; Supplementary Figure S1). Except TNF-α, the reference materials were not able to trigger changes in the TEER measurements (Table 4; Supplementary Figure S2).

#### 3.1.2 Tissue functionality

Tissue functionality was investigated through the assessment of CBF, MCC and mucin secretion. Even though there is evidence that some of the reference materials (see Supplementary Table S1) have the potential to induce changes in those parameters, no bioactivity was observed in the MucilAir™-HF tissues (Table 4). Regarding the benchmark chemicals, only BE PVM/MA reduced CBF at 6 h exposure/day (Table 4; Supplementary Figure S1), whereas CMC at 30 min exposure/day promoted a concentration-dependent reduction in the MCC only on day 1 (Table 4; Figure 5), suggesting that this finding may be of no concern due to the fast reversibility (Ugwoke et al., 2000). Regarding mucin production, PHMG triggered increased mucin secretion at 6 h exposure/day, mainly from day 8 onward; a similar effect was observed for Coumarin at 30 min exposure/day, but not at 6 h exposure/day (Table 4; Figure 5; Supplementary Figure S1).

The presence of mucin-producing goblet cells was investigated using Muc5AC immunohistochemical staining (Table 4; Supplementary Figure S3; Supplementary Table S2). It was observed that the goblet cell hyper-, metaplasia agent IL-13 triggered an expected 4-fold increase in cells expressing Muc5AC protein, when compared to unexposed tissues. Similarly, Isoproterenol also induced a 2.5- and 3-fold increase in this parameter at 50 and 100 μM, respectively. However, other test materials were not able to promote any changes in Muc5AC protein expression, including CFTR_inh_-172, experimentally used to mimic the inflammatory profile found in cystic fibrosis, a disease marked by mucus hyperproduction.

#### 3.1.3 Modulation of cytokines and chemokines

Quantification of different cytokines and chemokines was performed with focus on those proteins involved in the inflammation (CCL2, CCL7, CCL26, CXCL10, CXCL11, ICAM-1, IL-1α, osteopontin, IFN-γ, TNF-α, IL-6, and IL-8), degradation of extracellular matrix/fibrosis (MMP-1, MMP-2, MMP-3, MMP-7, MMP-9, TIMP-1, uPAR, uPA, serpin E1, and TGF-β1) and anti-inflammatory (IL-1ra) responses, as summarized in Table 4.

In general, benchmark chemicals (Akemi, Acrylate copolymer, BE PVM/MA, PHMG, and BAC) and reference materials (LPS, Isoproterenol, and TNF-α) produced differentially modulated cytokines and chemokines release over the 12-day experimental period (Table 4; Figure 5; Supplementary Figures S1, S2). Those materials exposed at 6h/day showed a tendency to induce stronger changes in the investigated biomarkers release, demonstrating that the exposure time was related to the extent of the induced biological effects. For instance, LPS, a material well-known for its ability to induce inflammatory responses, did not promote modulation of the different cytokines/chemokines at 30min/day exposure (Table 4); however, the 6h/day exposure triggered increased levels of proteins involved in the inflammatory response (e.g., IL-6, IL-8, and TNF-α) and degradation of extracellular matrix/fibrosis (e.g., MMP-3 and uPAR) (Table 4; Supplementary Figures S1, S2).

Regarding inflammatory modulation, BE PVM/MA (Figure 5) and the reference material TNF-α (Supplementary Figure S2), both at 30 min/day exposure, were able to trigger the upregulation of CCL2 and osteopontin. Also, the former induced production of TNF-α itself and of IL-8 (Supplementary Figure S2), concordant with literature data using other lung cell models (see Supplementary Table S1). At 6h/day exposure, BE PVM/MA triggered upregulation of a higher number of biomarkers, such as IL-6 and IL-8 (Supplementary Figure S1). Similarly, PHMG triggered increased levels of CXCL10 only at 30 min/day exposure (Figure 5), while the 6h/day exposure induced the upregulation of CXCL10, IL-6, and IL-8 (Supplementary Figure S1). Also, BAC promoted an increase in CCL7, IL-1α and IL-6, mainly at high concentration (Figure 5). Interestingly, Acrylate copolymer promoted a dual effect related to its time of exposure in MucilAir™-HF model: a downregulation of some released inflammatory biomarkers at 30 min/day exposure (Figure 5), whereas it triggered an upregulation profile at 6h/day exposure (Supplementary Figure S1).

Concerning the degradation of extracellular matrix/fibrosis, PHMG and TNF-α did not induce any modulation of related biomarkers, even though they have triggered the modulation of inflammatory cytokines (Table 4; Figure 5). BAC and Isoproterenol promoted downregulation of MMP-7 only (Figure 5), while Akemi (Supplementary Figure S1) and LPS (Supplementary Figure S2) induced upregulation of MMP-3 and both MMP-3/uPAR, respectively, at 6 h/day exposure. On the other hand, Acrylate copolymer and BE PVM/MA showed changes in a high number of biomarkers, such as MMP-3 and MMP-7 (Figure 5; Supplementary Figure S1).

In general, test materials promoted mainly upregulation of IL-6, IL-8 and MMP-3 and downregulation of MMP-7 levels. Regarding the anti-inflammatory modulation, IL-1ra showed upregulated levels induced by BE PVM/MA, PHMG, and BAC (Figure 5; Supplementary Figures S1, S2). As IL-1ra has been linked to modulation of inflammation in cystic fibrosis (Fritzsching et al., 2015), this finding suggests its role in preserving cell function against the inflammatory process induced by those chemicals.

### 3.2 Effects of materials in the lower respiratory tract EpiAlveolar™ model

#### 3.2.1 Histology and immunohistochemistry analyses

To evaluate the quality of EpiAlveolar™ tissues over the 12-day experimental period, histological and immunohistochemistry assessments were performed by Laboratories 1 and 2. In addition, changes induced by the test materials were also performed by Laboratory 2. Detailed findings can be found in Supplementary Material S2.

In general, the cell morphology and viability of the tissues were not significantly impacted. However, Laboratory 1 observed that overall cellularity appeared to be decreased with flattening/thinning of cell layers from day 8 onward, while Laboratory 2 found early signs degeneration with a minor increase in thinning of the epithelial layers and a minor increase in numbers of degenerate cells from day 4 onward. Both laboratories also observed expected expression patterns for pan-cytokeratin, vimentin and/or aquaporin 5; nevertheless, no strong pro-surfactant C staining was evident over 12-day period, suggesting that type II pneumocytes were not present, contrasting with previous findings (Barosova et al., 2020). Moreover, rare CD68 positive macrophages, seen on days 1 and 4, were not found later by Laboratory 2, suggesting that those cells likely vanished out of the system due to tissue washing off steps procedures.

Moreover, Laboratory 2 observed that, in general, the concentration related changes induced by the benchmark chemicals ranged from increased cell degeneration, separation/detachment, multifocal thinning (e.g., Crystalline silica, Amorphous silica, and Doxorubicin) and with more severe injury locally extensive cell death/necrosis (e.g., PHMG and Akemi).

#### 3.2.2 Tissue barrier integrity loss

Over the 12-day experimental period, Crystalline silica, Amiodarone, and the reference materials LPS and Sulforaphane showed no effects on barrier integrity in either laboratory. The same results were observed for Akemi, Amorphous silica, and Doxorubicin in Laboratory 1, in contrast to Laboratory 2 which observed changes induced by those chemicals at high concentrations (Table 5; Figure 6).

Regarding PHMG (Figure 6), different concentrations were tested by both laboratories. In Laboratory 2, PHMG (0.1–9.4 μg/cm^2^) triggered a marked concentration-dependent tissue barrier integrity loss at all concentrations on the first day of the experiment. From day 4 on, TEER values were at the background level, indicating that the tissue barrier was irreversibly perturbed. In view of this, lower concentrations (0.005–0.2 μg/cm^2^) were tested by Laboratory 1. Results showed that only the highest concentration triggered a slight barrier integrity loss on day 4. This loss was markedly increased until the end of the experiment, therefore, this concentration triggered barrier integrity loss in a time-dependent manner.

#### 3.2.3 Modulation of cytokines and chemokines

As with upper respiratory tract toxicity assessments using the MucilAir™-HF model, quantification of cytokines/chemokines was performed in test material-exposed EpiAlveolar™ tissues. Data are shown in Table 5; Figure 6.

In Laboratories 1 and 2, the reference positive material LPS, a well-known inflammatory agent, failed to induce changes in the levels of the investigated cytokines and chemokines. Also, as expected, the reference negative material Sulforaphane did not trigger any marked changes in such proteins. Moreover, in both laboratories, PHMG showed concordant outcomes regarding the modulation of the inflammatory, degradation of extracellular matrix/fibrosis, and anti-inflammatory responses, as expected. However, different patterns in the up- or downregulation of biomarkers were observed due to different concentrations tested by both laboratories (i.e., tested concentrations range from 0.005–0.2 μg/cm^2^ or 0.1–9.4 μg/cm^2^ in Laboratories 1 and 2, respectively). In Laboratory 1, it was observed a tendency to increase the release of the investigated proteins at high tested concentration. On the other hand, in Laboratory 2, where a marked tissue barrier integrity loss was observed for all tested concentrations, a downregulation profile was observed. Also, IL-1ra showed upregulation profile initially for all concentrations until day 8 followed by biphasic response from day 8 onward.

Despite having shown changes in different biomarkers induced by Doxorubicin, both laboratories also observed a tendency towards downregulation of the inflammatory and degradation of extracellular matrix/fibrosis responses; also, a dual effect with the anti-inflammatory IL-1ra response was observed by the Laboratory 1 only. Moreover, a tendency for downregulation of biomarkers related to inflammation and/or degradation of extracellular matrix/fibrosis was observed for Amorphous silica, Crystalline silica, and Akemi only by Laboratory 2. Regarding Amiodarone, both laboratories observed upregulated levels of ICAM-1, but only Laboratory 2 showed downregulated levels of MMP-1 and MMP-2.

Taking the results found in both laboratories together, levels of CXCL10, IL-8, IL-1α, matrix metalloproteinases (MMP-1, -2, -3, -7, and -9), uPA, and serpin E1 had greater magnitude of response compared to other investigated readouts. Given the range of proteins evaluated, this finding suggests the fundamental role of these readouts in the bioactivity induced by the benchmark chemicals.

#### 3.2.4 Changes in GSH and GSSG levels and mitotoxicity

No benchmark chemical induced alterations in GSH and GSSG levels (Table 5; Supplementary Material S4), suggesting that the GSH pathway was not linked to oxidative stress induced by some chemicals, e.g., PHMG. Nonetheless, effects in mitochondria were observed for Akemi, Crystalline silica and PHMG at higher concentrations mainly (Table 5; Figure 6).

#### 3.2.5 Elucidation of mechanism of action using transcriptomics

Here, we explored the potential utility of transcriptomics as a technology, not only for establishing a PoD but also for gaining mechanistic insights to generate hypotheses within the context of a risk assessment framework. Therefore, we set out to investigate if, by using this type of analysis, the mechanisms of lung toxicity (especially pulmonary fibrosis) associated with the benchmark chemicals could be identified.

Figure 7 displays the LVs that showed significant concentration- and time-dependent responses after benchmark chemical exposure relative to the vehicle control. The number of LVs altered increased over time, with maximum effects observed at day 12 for all chemicals. The most active chemicals were PHMG (n = 22), Amorphous silica (n = 15) and Doxorubicin (n = 12), followed by Crystalline silica (n = 6), Akemi (n = 5), Amiodarone (n = 5), and the reference materials LPS (n = 4) and Sulforaphane (n = 1). Importantly, most of the LVs modulated by PHMG, Amorphous silica, and Doxorubicin captured biological activity corresponding to the key factors leading to pulmonary fibrosis (Sieber et al., 2023; Todd et al., 2012): inflammation, oxidative stress, epithelial-mesenchymal transition which ultimately leads to excessive deposition of extracellular matrix. For example, LV62, LV80, and LV76, have strong associations with pathways such as “extracellular matrix organization,” “epithelial mesenchymal transition,” and “collagen formation.” In addition, LV7 and LV11 contain bioactivity related to inflammation, keratinization and oxidative stress (see Supplementary Material S5). It is worth mentioning that several LVs share the same pathways, e.g., keratinization can be found for LVs 2, 7, 62, and 80, corroborating the involvement of this pathway in the bioactivity induced by the materials (Supplementary Figure S4). In a risk assessment context this information would suggest that PHMG, Amorphous silica, and Doxorubicin could cause pulmonary fibrosis *in vivo* and would warrant further investigation. This is consistent with the evidence of pulmonary fibrosis observed in humans and animal models after exposure to PHMG (Kim et al., 2021; Park et al., 2014) and Doxorubicin (Minchin et al., 1988; Nevadunsky et al., 2013). In contrast, research on the health effects of Amorphous silica has shown that the initial inflammatory and fibrogenesis response is reversible in animal studies (Sun et al., 2016; Weber et al., 2018), supported by the long-term safe use in products and based on epidemiological studies from occupational exposures (ECETOC, 2006; Merget et al., 2002; Yong et al., 2022). Surprisingly, only 3 out of the 24 pathways perturbed by Crystalline silica, a well-known pro-fibrotic compound, were related to fibrosis.

For the water repellent polymer (Akemi), LVs 7, 11, 30, and 33 contain pathways related to fibrosis. Whether this compound leads to this adverse outcome *in vivo* is unknown, as all existing human (Duch et al., 2014) and animal data is limited to short-term exposures and concentrates on its effects on the lung surfactant (Sørli et al., 2018).

Amiodarone-induced pulmonary fibrosis develops in 5%–7% of patients following typical Amiodarone pneumonitis (Budin et al., 2022). There are several mechanisms potentially involved, however, it has been suggested that mitochondrial dysfunction could play a critical role (Bolt et al., 2001). Amiodarone has been shown to cause uncoupling of oxidative phosphorylation, and inhibition of the electron transport chain and fatty acid β-oxidation (Fromenty et al., 1990a; Fromenty et al., 1990b). Most of the pathways perturbed by Amiodarone in this dataset are associated with these mechanisms (LVs 28 and 81), for example the pathways: “oxidative_phosphorylation,” “reactome_complex_i_biogenesis,” “reactome_fatty_acyl_coa_biosynthesis.”

For the reference negative chemical, Sulforaphane, only one LV was weakly modulated (LV 31). While it was not possible to derive a mechanism of lung toxicity for LPS from this dataset, for most of the other benchmark chemicals, this analysis was able to provide further insights into their putative mechanism of toxicity.

### 3.3 Comparison between PoD and exposure estimates for the use scenarios

Figure 8 shows the PoD medians for all readouts in which benchmark chemical-induced bioactivity (either up or downregulation) were observed. The corresponding exposures predicted for day 12 for upper and lower respiratory tract regions are shown as vertical lines. A comparison was performed between the predicted exposure using *in silico* methods (described in item 2.3) and the measured bioactivity responses (i.e., PoDs as determined using the state-space model approach). The exposure was taken from the maximum predicted value across all relevant generations on that day (1, 4, 8, and 12), which was recorded for both the upper and lower lung (Table 3; Supplementary Material S1). Corresponding BERs for all benchmark chemical scenarios are shown in Supplementary Figure S5. Plots of *in vitro* PoDs and calculated exposures for each day are shown in Supplementary Figures S6, S7.

For the low-risk consumer product use scenarios, namely Amorphous silica (antiperspirant), BAC (nasal and cleaning sprays), Acrylate copolymer (hair spray) and Coumarin (anti-perspirant), the exposure levels were lower than minimum observed PoDs, as expected. The BERs obtained for day 12 in these scenarios were between ∼25,000 for Coumarin and ∼5 for Acrylate copolymer. In these five cases, the airborne concentration and particle size obtained from the SUET data were used as inputs into the calculation performed with MPPD (Supplementary Material S1), which likely helped to make the parameters used to model said scenarios more realistic.

For the occupational exposure low-risk scenarios for Amorphous and Crystalline silicas, the exposure values were much higher relative to the PoDs, leading to BERs less than 1. This may be attributed to the very low clearance rate in the ICRP model for lower respiratory tract clearance or to the sensitive transcriptomics PoDs. In general, it is found that the bioactivity observed with the transcriptomics analysis was more sensitive to changes than for other readouts investigated using the EpiAlveolar™ model (Figure 8; Supplementary Figures S6, S7). For Crystaline silica, for both the occupational low- and high-risk scenarios, the predicted lower respiratory exposure was higher than the minimum PoD from transcriptomics, but neither scenario led to exposures which exceed the minimum PoDs for other readouts.

Since both BAC and one of the investigated CMC scenarios were for nasal sprays, only the upper respiratory tract was considered in this situation. In the case of CMC, only one readout (MCC) had an observed PoD in the MucilAir™-HF model, which was only observed on day 1 and was greater than the predicted exposure (BER ≈ 2.4) (Figure 8; Supplementary Figures S5–7).

For the remaining two chemicals studied in this work, PHMG and Akemi (humidifier and tile coating product scenarios, respectively), our findings agreed with the high-risk classification for all scenarios investigated. For both chemicals, the predicted exposures exceeded even the largest PoD on all days measured, corresponding to a Day 12 BER of approximtely 10^–5^ for PHMG and and 10^–4^ for Akemi.

For both non-aerosolised drugs, Doxorubicin and Amiodarone, covering therapeutic dose high-risk scenarios, the exposure (i.e. maximum plasma concentration C_max_) was taken from literature values (see Table 3). For Doxorubicin, this led to a very high exposure which exceeded the PoD for all readouts for all days, corresponding to BERs between 0.0005–0.2. Amiodarone by contrast, gave more variable results, with the C_max_ exceeding the transcriptomics PoDs at all timepoints (minimum BER of 0.004) but higher PoDs from the other readouts with BERs between 0.5 and 3.5 depending on timepoint and laboratory.

## 4 Discussion

Following the trends and perspectives of the toxicity testing in the 21st Century vision (National Research Council, 2007), there is an ongoing need to develop robust and relevant tools and approaches that can be used to characterize the kinetics, bioactivity and risk of chemicals using NGRA without generation of new animal data. For assessing respiratory toxicity, ALI human lung models have shown promise, as they represent a complex tissue structure that physiologically recapitulates many aspects of the human respiratory epithelium as well as allowing *in vivo*-like exposure to pulmonary toxicants (Bedford et al., 2022; Cao et al., 2021; Xiong et al., 2021). In this context, the present work evaluated the applicability of the commercially available upper MucilAir™-HF and lower EpiAlveolar™ human lung models to be used as *in vitro* systems within a NAM toolbox to identify the bioactivity (i.e., *in vitro* PoD derivation) and risk of materials that may reach the respiratory tract and induce lung toxicity. A 12-day substance exposure protocol was established to explore potential adverse effects in the lung following repeated exposure. The obtained PoDs were combined with exposure estimates using MPPD and ICRP modeling to calculate BER values. The performance of this approach is determined by its ability to differentiate between chemical/exposure scenarios of low and high risk to humans based on the size of the BER. In this pilot work, we have not conducted a formal evaluation as previously reported for NGRA approaches for systemic toxicity (Cable et al., 2024; Middleton et al., 2022), but rather we have investigated a proof-of-concept to establish the feasibility of defining a NAM toolbox to be included within an NGRA approach for lung toxicity. For the 11 benchmark chemicals (Table 3; Figure 8) investigated in 14 human exposure scenarios, it was possible to correctly separate their risk classification by using the lowest BER per readout and lung model. Generally, for the low-risk chemical-exposure scenarios (except Crystalline and Amorphous silica occupational scenarios), the PoDs occurred at higher concentrations than the corresponding human exposure values, whereas for all the high-risk chemical-exposure scenarios, there was a clear overlap between the PoDs and lung deposited mass (benchmark inhaled materials) or maximum plasma concentration (systemically available benchmark drugs). This is only true if the transcriptomic PoDs are included in the analysis. If the transcriptomics analysis were not included, results for the high risk Crystalline silica scenario would have clustered with the low-risk chemical-exposure scenarios. One of the limitations of this work was that transcriptomics was not performed in a concentration-response manner for the upper MucilAir™-HF respiratory model, and therefore not all benchmark chemicals have transcriptomics PoDs.

Risk assessments for human inhalation toxicity based on traditional animal studies generally include a safety factor of 25 to account for uncertainties related to interspecies (animal-to-human: 2.5-safety factor) and inter-individual (human-to-human: 10-safety factor) variabilities (ECHA, 2012). Therefore, a margin of safety over 25 compared to no observed adverse effects levels in animals has been judged to be protective for human health for several decades regarding local lung effects. Defining a safe BER threshold or the appropriate use of uncertainty factors remains a challenge in NGRA. A recent regulatory example, accepted by the US EPA, of a non-animal risk assessment for the fungicide chlorothalonil in an occupational scenario combined *in vitro* PoDs from MucilAir™ readouts (i.e., TEER, lactate dehydrogenase leakage and resazurin metabolism analyses) with dosimetry information obtained from a computational fluid-particle dynamics (CFPD) model (Corley et al., 2021; Li et al., 2018; McGee Hargrove et al., 2021; Ramanarayanan et al., 2022). In this specific case, the total uncertainty safety factor, to account the response among human population, was 3 considering inter-individual toxicodynamic variability only. The inter-individual toxicokinetic and interspecies uncertainty factors were waived on the premise that the *in vitro* model was derived from human lung biopsies, the readouts measure MIEs for the mode of action (direct acting contact irritation) and the exposure modelling is sufficiently realistic and accurate. Applying a BER threshold of 3 to our dataset would be protective for all benchmark chemicals, only when including the transcriptomics PoDs for the high-risk exposure scenarios for Amiodarone and Crystalline silica. To be able to identify low-risk BER thresholds that would be protective for a range of chemicals and human exposure scenarios would require additional work to characterize further the uncertainties in both the exposure and toxicodynamic parts of our NAM toolbox, as discussed below.

Our work addressed the challenge of bridging the gap between realistic human exposures and toxicological responses, employing the widely used MPPD model. Although upper respiratory tract CFD models offer detailed dose resolution as demonstrated in the chlorothalonil case study, conducting full simulations in the lower respiratory tract proved computationally burdensome due to the vast scale of the human pulmonary system (Corley et al., 2021; Kuempel et al., 2015). Therefore, such models would be a potential next step in a tiered approach to exposure assessment if refinement is needed. Recent studies attempted to overcome this by combining upper respiratory CFD models with lower respiratory multi-path models, albeit at a considerable computational expense (Kuprat et al., 2021; Kuprat et al., 2023). Limitations of the MPPD model include its inability to predict hotspots forming at airway junctions and points of extreme curvature, potentially leading to underestimated local doses. A significant source of inaccuracy in *in silico* modeling arises from the ICRP model used to predict chemical clearance within the lung. The lower respiratory clearance model, calibrated from clinical data on the clearance of radioactive dust particles from worker exposures (ICRP, 1994), may be excessively conservative when applied to small molecules. In the exposure scenarios explored in this paper, a combination of literature values and experimentally measured data formed the basis for estimates. Where data was lacking, a conservative approach was taken, as exemplified by assuming a MMAD of 3 µm for Crystaline silica and Akemi, predicting the highest lower respiratory exposure upon inhalation. Despite employing conservative assumptions in this work, the primary source of uncertainty stems from the absence of any formal guidance on the parameterization of these models and validation data, unlike the well-established validation of physiologically based kinetic models against human clinical data (Li et al., 2021; Punt et al., 2022).

The *in vitro* lung models used in this work demonstrated some limitations which impacted on their sensitivity to some toxicants and their ability to replicate expected physiological responses. Despite selecting reference materials known for specific *in vitro* lung effects (Supplementary Table S1), some well-document biological responses to these materials were mild or absent in both *in vitro* human lung systems. For instance, tissue apical exposure to Chlorocresol failed to induce a CBF decrease in MucilAir™-HF tissues, and LPS via apical exposure did not trigger intense inflammatory cytokine secretion in EpiAveolar model, whereas a mild response was observed in MucilAir™-HF tissues exposed to TNF-α and LPS (at 6h/day aerosol exposure only); on the other hand, no effects were triggered by Nicotine and Sulforaphane, both via aerosol exposure, similar to other *in vitro* systems (see details in Supplementary Table S1). The absence of dendritic cells in MucilAir™-HF tissues might have led to the lack of marked inflammatory response to LPS and TNF-α, while the lack of macrophages in EpiAlveolar™ tissues might have impacted the response to Crystalline silica, Amorphous silica, Amiodarone, and LPS given the lack of response in the cytokine/chemokine panel. However, the transcriptomics data provided insights about both the potency and mechanism of toxicity of these substances. Similarly, the absence of epithelial alveolar type II cells, the site of toxicity for the polymer contained in the Akemi formulation, did not impact classification of this chemical, with all PoDs generated in the EpiAlveolar™ tissues being lower than the predicted human exposure. Similar results were observed in the recently evaluated systemic NAM toolbox (Cable et al., 2024). While the toolbox was not always able to identify the critical mode of action for some chemicals, it was almost always able to correctly assess the risk of the chemical-exposure scenario based on a general measure of bioactivity, such as transcriptomics.

It was clear from our experience with two laboratories, that there is a need to develop and evaluate standardized protocols for dosing methodologies (e.g., Bannuscher et al., 2022), measurement of readouts [e.g., CBF and TEER analyses as proposed by Behrsing et al. (2022); Sharma et al. (2024), respectively], number of donor requirements, timepoints and duration of exposure. In this regard, recent research showed that apical liquid dosing in the primary human bronchial epithelial cell/lung fibroblast ALI co-culture model can reprogram gene expression and alter cell physiology, potentially introducing confounding factors that compromise the accuracy of inhaled substance evaluations (Mallek et al., 2024). Corroborating this, our data showed that LPS from *P*. *aeruginosa* 10, via aerosol exposure, induced upregulation of MMP-3 and uPAR levels in MucilAir™-HF tissues; however, no effects were observed when tissues were exposed to LPS via apical liquid dosing. Similarly, no effects were observed in EpiAlveolar™ tissues exposed, via apical, to LPS from *P*. *aeruginosa* 10 or *E*. *coli* 055:B5. Therefore, this may also explain the lack of marked inflammatory response induced by LPS, besides the use of different bacterial strain sources that can to significantly impact the results of *in vitro* and *in vivo* experiments (Ernst et al., 2021; Lin et al., 2020; Pulendran et al., 2001). Moreover, the *in vitro* systems used here were composed of primary cells derived from limited number of human samples. In fact, sample size estimates have indicated the need for a reasonable number of donors (n = 13–299) to detect 2-fold changes in a panel of inflammatory readouts (e.g., IL-6 and IL-8 transcripts) to account for interindividual variability in studies using an *in vitro* primary cell-based model exposed to ozone (Bowers et al., 2022). Therefore, an approach using multiple donors, donor-matched tissue and pooled-donor samples in ALI exposures may support the understanding of inter-individual variability within *in vitro* assays (Moreau et al., 2022) by ideally using a broad set of different inhaled materials covering a diversity regarding chemical structures.

Despite some of the limitations discussed above, the NAM toolbox for respiratory safety used in this work appeared to separate the low- and high-risk benchmark exposure scenarios for 12 out of the 14 scenarios evaluated when using the BER, suggesting that our strategy of selecting NAMs informed by AOPs associated with pulmonary toxicity, can provide relevant biological coverage. In addition, the benchmarking approach provides an alternative to the traditional validation of NAMS against apical effects in rodent studies but rather tries to evaluate the NAMs in the context of making protective safety decisions (Browne et al., 2024). Our findings pave the way for further evaluation of the performance of NAM toolboxes for a wider substance dataset with varied mechanisms of action, uses, and balanced low and high-risk benchmarks to build confidence in the protectiveness of the approach. In addition, there is a need to establish scientific confidence by improving the reproducibility, standardization of protocols, and *in vitro* culture methodologies (ICCVAM, 2024; van der Zalm et al., 2022). This pioneering work together with future research will ensure confidence in the use of *in vitro* testing to generate human-relevant and mechanistically driven PoDs that, together with estimates of human exposure, can be used in safety assessments of ingredients in consumer goods products without the need for the generation of new animal data.

## Data Availability

The data related to transcriptomics analysis presented in the study are deposited in the European Bioinformatics Institute (EBI) data repository, Array Express (https://www.ebi.ac.uk/biostudies/arrayexpress), accession number E-MTAB-14272. The other original contributions presented in the study are included in the article/Supplementary Material, further inquiries can be directed to the corresponding author.

## Glossary

Acrylate copolymer: ACUDYNE™ DHR copolymer
Akemi: AKEMI^®^ anti-fleck super
ALI: air-liquid interface
AOP: adverse outcome pathway
BAC: Benzalkonium chloride
BE PVM/MA: Butyl ester of poly (methyl vinyl ether-alt-maleic acid monoethyl ester) copolymer
BER: bioactivity exposure ratio
**C**_**max**_: maximum plasma concentration
CBF: cilia beating frequency
CCL: chemokine (C-C motif) ligand
CDS: Concentration Dependency Scores
CFPD: computational fluid-particle dynamics
Chlorocresol: 4-Chloro-3-methylphenol
CMC: Carboxymethylcellulose sodium salt
COPD: chronic obstructive pulmonary disease
CXCL: C-X-C motif chemokine ligand
DART: development and reproductive toxicity
FCS: fetal calf serum
GSH: Reduced glutathione
GSSH: oxidized glutathione
HBSS: Hanks’ Balanced Salt solution
ICAM-1: intercellular adhesion molecule-1
ICRP: International Commission on Radiological Protection
IFN: interferon
IL-1Ra: IL-1 receptor antagonist
KE: key event
LPS: lipopolysaccharide
LV: latent variable
MCC: mucociliary clearance
MIE: molecular initiating event
MMAD: mass median aerodynamic diameters
MMP: matrix metalloproteinase
MPPD: multiple path particle dosimetry
Muc5AC: mucin-5AC protein
NAMs: New Approach Methodologies
NGRA: next-generation risk assessment
NOAEC: no observed adverse effect concentration
NOEC: no observed effect concentration
PBS: phosphate-buffered saline
PHMG: polyhexamethylene guanidine phosphate
PLIER: Pathway-level information extractor
PoD: point of departure
QC: quality control
QCM: quartz crystal microbalance
REACH: Registration, Evaluation and Authorization of Chemicals
SMC: Sequential Monte Carlo
SUET: simulated use evaluation testing
TEER: trans-epithelial electrical resistance
TIMP-1: tissue inhibitor of metalloproteinase 1
TNF: tumour necrosis factor
TWA: time-weighted average
uPA: urokinase
uPAR: urokinase-type plasminogen activator receptor
