# Supplementary material for: Evaluation of a non-animal toolbox informed by adverse outcome pathways for human inhalation safety
Source: Front Toxicol. 2025 Feb 21;7:1426132. doi: 10.3389/ftox.2025.1426132 (PMC11885506; doi:10.3389/ftox.2025.1426132)
Supplement: Supplementary file 2 [file Table1.docx]

**Supplementary Tables and Figures**

Evaluation of a non-animal toolbox informed by adverse outcome pathways (AOPs) for human inhalation safety

**Renato Ivan de Ávila**^1^**, Iris Müller**^1^**, Hugh Barlow**^1^**, Alistair Mark Middleton**^1^**, Mathura Theiventhran**^1^**, Danilo Basili**^1^**, Anthony M. Bowden**^1^**, Ouarda Saib**^1^**, Patrik Engi**^1^**, Tymoteusz Pietrenko**^1^**, Joanne Wallace^2^, Bernadett Boda^3^, Samuel Constant^3^, Holger Peter Behrsing^4^, Vivek Patel^4^, Maria Teresa Baltazar**^1*^

^1^Safety, Environmental and Regulatory Science (SERS), Unilever, Colworth Science Park, Sharnbrook, Bedfordshire, United Kingdom.

^2^Charles River Laboratories, Edinburgh, United Kingdom.

^3^Epithelix Sarl, Plan-les-Outes, Switzerland.

^4^Respiratory Toxicology Program, Institute for In Vitro Sciences, Inc., Gaithersburg, MD, United States.

**^*^Correspondence:**Corresponding Author: Maria Baltazar, PhD, maria.baltazar@unilever.com

**Supplementary Table S1.** Reference materials used to evaluate specific endpoints in MucilAir™-HF and/or EpiAveolar™ models over 12-day period.

| **Item/reason for testing** | **Bioactivity observed in this study and comparison to *in vitro* and *in vivo* human effects reported in the literature** |
| --- | --- |
| **Acrolein:** evidence of inducing CBF and mucin secretion increase | It is an α, β unsaturated aldehyde mainly formed during incomplete combustion and present in cigarette smoke, car exhaust fumes and biocides. It is highly reactive to molecules of the airway tissue, such as cellular antioxidant constituents (e.g. glutathione), therefore causing toxicity to the respiratory tract (Reddy et al., 2002). In adenovirus-12 SV40 hybrid virus transformed, non-tumorigenic human bronchial epithelial cells (BEAS-2B, Acrolein (3-10 µM) induced a concentration-dependent cytotoxicity through LDH leakage increase after 30-min exposure (Sthijns et al., 2014). *MUC5AC* mRNA expression increase by acrolein (0.03 µM for 4 h) involves phosphorylation of EGFR and MAPK2/3 in human pulmonary mucoepidermoid carcinoma cell line (NCI-H292), and increase of MMP9 mRNA expression in NCI-292 and normal human bronchial epithelial cells (Deshmukh et al., 2005). In human primary bronchial epithelial cells cultured at the air-liquid interface, Acrolein (0.1–1 mg/m^3^ for 30 min) triggered no significant changes in IL-8 and MMP-9 secretion levels and *MMP9* and *NFKB* mRNA expressions, but an increase in *TNFA* and *HMOX1* mRNA expressions (Dwivedi et al., 2018). In submerged primary nasal epithelial cell cultures, acrolein triggered 2-fold increase in IL-8 release (Comer et al., 2014). Human data showed no changes in IL-6 and IL-8 levels in induced sputum samples from volunteers exposed at six occasions for 2 h at rest to: clean air, 15 ppm ethyl acetate (EA), and 0.05 ppm and 0.1 ppm acrolein with and without EA (15 ppm) to mask the potential influence of odor (Dwivedi et al., 2015). In addition, there were no changes in different inflammation and coagulation biomarkers levels in blood (IL-6, C-reactive protein, serum amyloid A, fibrinogen, factor VIII, von Willebrand factor, and Clara cell protein) (Dwivedi et al., 2015). In a o human air-liquid-interface (ALI) airway tissue model based on cryopreserved normal human bronchial epithelial (NHBE) cells, Acrolein (125-1000 µM) for 10 min/day, via apical liquid application, for up to 10 days did not trigger cytotoxicity. In addition, release of pro-inflammatory cytokines (IL-1β, IL-6, IL-8, TNF-α, GM-CSF, and IFN-γ) was significantly enhanced 24 h after the first 10-min exposure. Changes in the beating frequency and structures of ciliated cells, inhibition of mucin expression and secretion apparatus, and development of squamous differentiation were also observed (Xiong et al., 2018).  In our study, MucilAir™-HF tissues exposed to Acrolein (500, 750 or 1000 µM), via apical liquid exposure, did not show any effects in tissue barrier integrity loss, tissue functionality and modulation of cytokines and chemokines related to inflammatory and anti-inflammatory responses and degradation of extracellular matrix/fibrosis modulation. |
| **CFTR_inh_-172:** agent that mimics the cystic fibrosis inflammatory process | CFTR_inh_-172 is a cystic fibrosis transmembrane conductance regulator (CFTR) inhibitor used to mimic the inflammatory profile found in cystic fibrosis, a disease marked by mucus hyperproduction. In cystic fibrosis (CFBE41o-), corrected CFBE41o- (CFBE41o-pCep4 overexpressing wtCFTR) and normal (16HBE14o-) airway epithelial cell lines, CFTR_inh_-172 (40 µM for 60 min) triggered a time-dependent increase in TEER values in the corrected and normal cell lines (Nilsson et al., 2010). It was observed no changes in granulocyte/macrophage colony-stimulating factor, IL-6 and/or IL-8 secretion levels in cystic fibrosis phenotype cells (9/HTEo- pCEP-R and 16HBE14o- AS cell lines and primary cystic fibrosis cells) (Perez et al., 2007) exposed to CFTR_inh_-172 (20 µM) for 3-5 days.  In our study, CFTR_inh_-172 (1, 10 or 100 µM) did not induce expected increase in mucin secretion or Muc5AC protein expression in MucilAir™-HF tissues as well as in any investigated parameter. |
| **Chlorocresol:** known to induce CBF decrease | Lipophilic preservative chlorocresol is a reference cilio-inhibitory compound. It has been shown that chlorocresol (0.005% w/ v for 1-20 min)  exerted reversible cilio-inhibitory effects by significantly decreasing CBF in primary human nasal epithelial cells (Uchenna Agu et al., 1999). Also, authors showed that it can trigger instantaneous and irreversible ciliostasis at a higher concentration (0.1% w/v).  In our study, Chlorocresol (1.3, 2.6 or 26 µg/cm^2^) was not able to induce changes in tissue functionality (e.g. reduced CBF) of the MucilAir™-HF system. Moreover, no effects were observed in tissue barrier integrity and modulation of cytokines and chemokines related to inflammatory and anti-inflammatory responses and degradation of extracellular matrix/fibrosis modulation. |
| **Isoproterenol hydrochloride:** known to induce CBF increase | Isoproterenol is an agonist of β_2_-adrenoceptors, widely distributed in the lung. It promotes activation, through beta-adrenergic receptors, of intracellular adenyl cyclase, the enzyme that catalyses the conversion of adenosine triphosphate (ATP) to cyclic AMP (Szymanski and Singh, 2023). Isoproterenol (1 µM for 48h) induced a significant increase in *MUC5AC* mRNA expression in NCI-H292 cells (Matsuyama et al., 2018). In a human bronchial epithelial cell line (16HBE14 o^−^ cells), a significant increase of IL-6 and IL-8 release was observed after exposure to Isoproterenol at 10-100 nM for 24h (Oehme et al., 2015). IL-8 levels were also increased in in human transformed bronchial epithelial cells (16HBE cells) exposed to Isoproterenol at 1-30 nM for 18h (Lindén, 1996). Moreover, Isoproterenol (10^-5^ and 10^-3^ M for 30-45 min) triggered maximally and irreversibly increased ciliary CBF of primary human nasal epithelial cells (Uchenna Agu et al., 1999).  Our findings showed that Isoproterenol (1, 50 or 100 µM) exposed via basal liquid in MucilAir™-HF tissues was able to trigger 2.5-3-fold increase in cells expressing Muc5AC protein, when compared to unexposed tissues. Also, it promoted downregulation of MMP-7 levels only |
| **Lipopolysaccharide (LPS):** respiratory tract inflammation agent | LPS is a component of the outer cell walls of Gram-negative bacteria that can promote respiratory tract inflammation likely due to uncontrolled signalling response induced by LPS binding to Tool-like receptor-4 (TLR4) present in several host cell types, e.g. monocytes and macrophages (Farhana and Khan, 2022). In cultured alveolar macrophages from subjects underwent fiberoptic bronchoscopy and cultured epithelial cells from surgically resected lung tissues from chronic obstructive pulmonary disease patients, LPS (10 µg/mL for 48h; origin: bacteria strain not specified) triggered significant increase in MMP-9 supernatant levels and mRNA transcripts (JOUNEAU et al., 2011). Also, LPS (1 μg/mL for 24h; origin: bacteria strain not specified) has shown to trigger significant increase of IL-6 and IL-8 protein and mRNA transcripts levels in primary human bronchial epithelial cells (Wu et al., 2010). In human A549 alveolar epithelial cells, LPS (10-1000 ng/mL for 12h; origin: bacteria strain not specified) triggered no statistically significant increase of TGF-β1 protein expression (Kwong et al., 2004). However, LPS (1 µg/mL for 24 h) from *Escherichia coli* 0111:B4 increased IL-8 protein and mRNA transcripts levels in A549 cells (Ye et al., 2009). Moreover, significant increases of TNF-α, IL-6, IL-8 and MUC5A mRNA and supernatant protein levels have been observed in HBE16 airway epithelial cells exposed to LPS (10 µg/mL; origin: bacteria strain not specified) for 12 h (Li et al., 2010).  Our data showed that LPS (from *Pseudomonas aeruginosa* 10) at 1.6 µg/cm^2^ induced upregulation of MMP-3 and uPAR at 6h/day aerosol exposure in MucilAir™-HF tissues; however, no effects were observed when tissues were exposed to LPS (0.2, 1.6 or 16 µg/cm^2^) via apical application. Similarly, no effects were observed in EpiAlveolar™ tissues exposed, via apical, to LPS (0.01, 0.1, 1 or 10 µg/mL) from *Pseudomonas aeruginosa* 10 or *Escherichia coli* 055:B5. |
| **Nicotine:** oxidative stress agent | Nicotine, the most known ingredient of cigarette smoke, can promote overgeneration of mitochondrial reactive oxygen species (ROS) and mimic the effects of hypoxia by activation of hypoxia-inducible factor (HIF)-1α. In lactate dehydrogenase (LDH) release assay, different concentrations of Nicotine (0.1-5 µM for 3h) did not trigger cytotoxicity in A549 human lung adenocarcinoma cell line (Guo et al., 2012). In EpiAirway-100 human upper respiratory tract epithelium model, Nicotine (25-125 mM for 24h) altered integrity of the epithelial membrane, showing a biphasic response with a large and significant increase of TEER values at low concentrations followed by a steady decrease at higher concentrations (Balharry et al., 2008). In HBE16 airway epithelial cells exposed at 20 μM for 12h, Nicotine did not trigger changes in TNF-α, IL-8, IL-6 and MUC5AC supernatant protein and mRNA levels (Li et al., 2010).  Our findings showed that MucilAir™-HF tissues exposed to aerosolised Nicotine did not show any changes in the investigated parameters, e.g. tissue functionality and modulation of cytokines and chemokines. |
| **Sulforaphane:** anti-inflammatory agent | Antioxidant agent sulforaphane is a metabolite of the glucoraphanin, a phytochemical derived from cruciferous vegetables, such as broccoli and cabbage. It exerts protective effects against cell damage by activating the nuclear factor erythroid 2 like 2 (Nrf2), which is involved in the protection from free radical-induced diseases, including chronic obstructive pulmonary disease (COPD). In RLE-6TN rat lung epithelial cells, sulforaphane (0.5 µM) has shown to protect from cigarette smoke extract-induced oxidative damage, possibly by upregulating Nrf2 expression and reducing ROS levels (Jiao et al., 2017). In a study involving LPS- or Pam3CysSerLys4 (Pam3CSK4)-induced inflammation in monocyte-derived macrophages (MDMs) from patients with COPD, sulforaphane (20 µM) promoted anti-inflammatory activity by suppressing the expression of Toll-like receptors (TLR2 and TLR4) and downstream myeloid differentiation factor 88 (MyD88) along with a reduction in the production of IL-6 and TNF-α (Zeng et al., 2021).  In MucilAir™-HF and EpiAlveolar™ tissues, Sulforaphane (0.1, 1.4, 2.9 µg/cm^2^) did not trigger any marked changes in the investigated parameters, as expected. |
| **TNF-α:** known to induce inflammation and evidence of inducing CBF increase | TNF-α is a potent proinflammatory agent involved in some inflammatory diseases, e.g. asthma. Its inflammatory activity is mediated via binding to cell surface receptors type 1 and 2 (TNFRI and TNFRII, respectively) expressed in several cell types such as immune and endothelial cells. The receptor-ligand interactions trigger phosphorylation of nuclear factor-κB (NF-κB) that, in turn, promotes increase in the transcription of pro-inflammatory genes such as IL-8, IL-6 and TNF-α itself (Aggarwal et al., 2012). In cultured human airway smooth muscle cells, this agent at 1-500 U/mL for 24h has promoted a significant increase of IL-6 production protein and mRNA transcript levels (McKay et al., 2000). Moreover, increased IL-8 protein levels were observed in human bronchial epithelial cells (Beas2B), human lung fibroblasts (HFL-1), and human transformed lung epithelial cells (H292) exposed to TNF-α at 10 ng/mL for 24h (Gerloff et al., 2017). Also, MUC5AC protein and mRNA levels were increased in NCI-H292 cells exposed to TNF-α at 20 ng/mL for 12h (Lee et al., 2016).  In MucilAir™-HF tissues, our findings showed that TNF-α (10, 50, 100 ng/mL) was able to trigger changes in tissue barrier integrity, mainly at high concentrations. Also, TNF-α was able to induce upregulation of CCL2, osteopontin, IL-8 and TNF-α itself. |

**Supplementary Table S2.** Presence of goblet cells assessed following immunohistochemistry analysis for mucin-5AC (Muc5AC) protein detection in MucilAir™-HF tissues (n=4 tissues/substance concentration).

| **% of Muc5AC area** | | | | | | | |
| --- | --- | --- | --- | --- | --- | --- | --- |
| **Group** | | **Exposure** | | | | | |
|  |  | **30 min/day** | | | **6 h/day** | | |
| Unexposed | | 6.48 ± 0.57 | | |  | | |
| Saline (liquid) | | 7.83 ± 1.55 | | | 2.61 ± 0.48 | | |
| Saline (aerosol) | | 5.77 ± 1.60 | | | 2.16 ± 0.25 | | |
| Cytomix^*^ | | 8.24 ± 2.88 | | |  | | |
| IL-13 (10 ng/mL) | | 26.35 ± 3.34 | | |  | | |
| **Test Material Group**  **(tested concentrations)** | | **Concentration** | | | | | |
|  |  | **Low** | | **Medium** | **High 1** | | **High 2** |
| 30 min/day Exposure | Akemi (1, 6, 12 µg/cm^2^) | 4.45 ± 0.79 | 5.69 ± 1.54 | | 4.02 ± 0.60 |  | |
|  | Acrylate copolymer (0.1, 10, 100 µg/cm^2^) | 4.95 ± 1.23 | 5.45 ± 0.67 | | 5.68 ± 0.53 |  | |
|  | BE PVM-MA (0.1, 10, 100 µg/cm^2^) | 3.01 ± 0.33 | 4.55 ± 1.37 | | 5.81 ± 0.85 |  | |
|  | Coumarin (0.5, 4.7, 9.4 µg/cm^2^) | 5.09 ± 0.57 | 6.83 ± 1.07 | | 3.29 ± 0.36 |  | |
|  | PHMG (0.8, 2.4, 4.8 µg/cm^2^) | 0.67 ± 0.15 | 1.95 ±0.68 | | 0.40 ± 0.18 |  | |
|  | LPS (0.2, 1.6, 16 µg/cm^2^) | 2.87 ± 0.77 | 4.30 ± 0.90 | | 4.28 ± 0.76 |  | |
|  | CMC (2.5, 5, 10, 100 µg/cm^2^) | 4.86 ± 1.14 | 6.43 ± 2.04 | | 7.02 ± 2.66 | 6.58 ± 2.25 | |
|  | Nicotine (0.04, 0.4, 4 µg/cm^2^) | 0.89 ± 0.62 | 3.91 ±0.79 | | 5.94 ± 1.90 |  | |
|  | BAC (0.1, 0.5, 5, 10 µg/cm^2^) | 5.75 ± 0.93 | 5.37 ± 1.06 | | 3.98 ± 1.12 | ND | |
|  | Acrolein (500, 750, 1000 µM) | 3.66 ± 1.21 | 7.40 ± 1.45 | | 5.21 ± 2.16 |  | |
|  | Chlorocresol (1.3, 2.6, 26 µg/cm^2^) | 14.47 ± 0.87 | 8.85 ± 1.77 | | 6.95 ± 0.30 |  | |
|  | Isoproterenol (1, 50, 100 µM) | 8.78 ± 1.62 | 15.53 ± 1.41 | | 18.91 ± 3.09 |  | |
|  | CFTR_inh_-172 (1, 10, 100 µM) | 7.15 ± 1.06 | 11.01 ± 0.81 | | 6.93 ± 0.51 |  | |
|  | TNF-α (10, 50, 100 ng/mL) | 8.18 ± 1.54 | 9.15 ± 6.07 | | 5.21 ± 3.28 |  | |
|  | Sulforaphane (0.1, 1.4, 2.9 µg/cm^2^) | 3.74 ± 0.71 | 9.38 ± 0.80 | | 5.04 ± 0.71 |  | |
| 6 h/day Exposure | Akemi (6 µg/cm^2^) |  | 1.54 ± 0.67 | |  |  | |
|  | Acrylate copolymer (100 µg/cm^2^) |  | 1.41 ± 0.23 | |  |  | |
|  | BE PVM-MA (100 µg/cm^2^) |  | 5.82 ± 1.46 | |  |  | |
|  | Coumarin (4.7 µg/cm^2^) |  | 2.85 ± 0.43 | |  |  | |
|  | PHMG (2.4 µg/cm^2^) |  | 0.28 ± 0.14 | |  |  | |
|  | LPS (1.6 µg/cm^2^) |  | 1.58 ± 0.40 | |  |  | |
|  | Sulforaphane (1.4 µg/cm^2^) |  | 2.55 ± 0.62 | |  |  | |

Data represent the percentage of Muc5AC surface (average ± standard deviation) measured in MucilAir™-HF tissues (n=3 cultures/group) at the end of experiment (day 12).

^*^Solution containing TNF-α (500 ng/mL), LPS (0.2 mg/mL), fetal calf serum (1 %, v/v).

ND: not determined due to tissue was significantly damaged at 10 µg/cm^2^ BAC, and it was lost during staining procedure.

**Supplementary Figure S1.** Benchmark chemical-induced changes in the upper respiratory MucilAir™-HF tissues. Tissues were exposed to the chemicals, through aerosol (^Ar^) or via apical (^AL^)/basal liquid (^BL^) application, once a day repeatedly for 6 h/day on 12 consecutive days with 3-4 different concentrations. On days 1, 4, 8 and 12, the following parameters were evaluated: barrier integrity through transepithelial electrical resistance (TEER) measurements analysis; tissue functionality by the assessment of cilia beating frequency (CBF), mucociliary clearance (MCC) and mucin secretion; inflammatory, degradation of extracellular matrix/fibrosis, and anti-inflammatory responses through cytokine and chemokine quantification. Figure shows only chemicals that triggered changes in the investigated parameters when compared to unexposed tissues groups. Blue and red colours represent down- or up-regulation, whereas the colour variation shows the intensity of such effects. For all substances, a comparison between the concentration- and time-dependent response analysis and corresponding readout data can be found in the Suppl. Data 4.

**
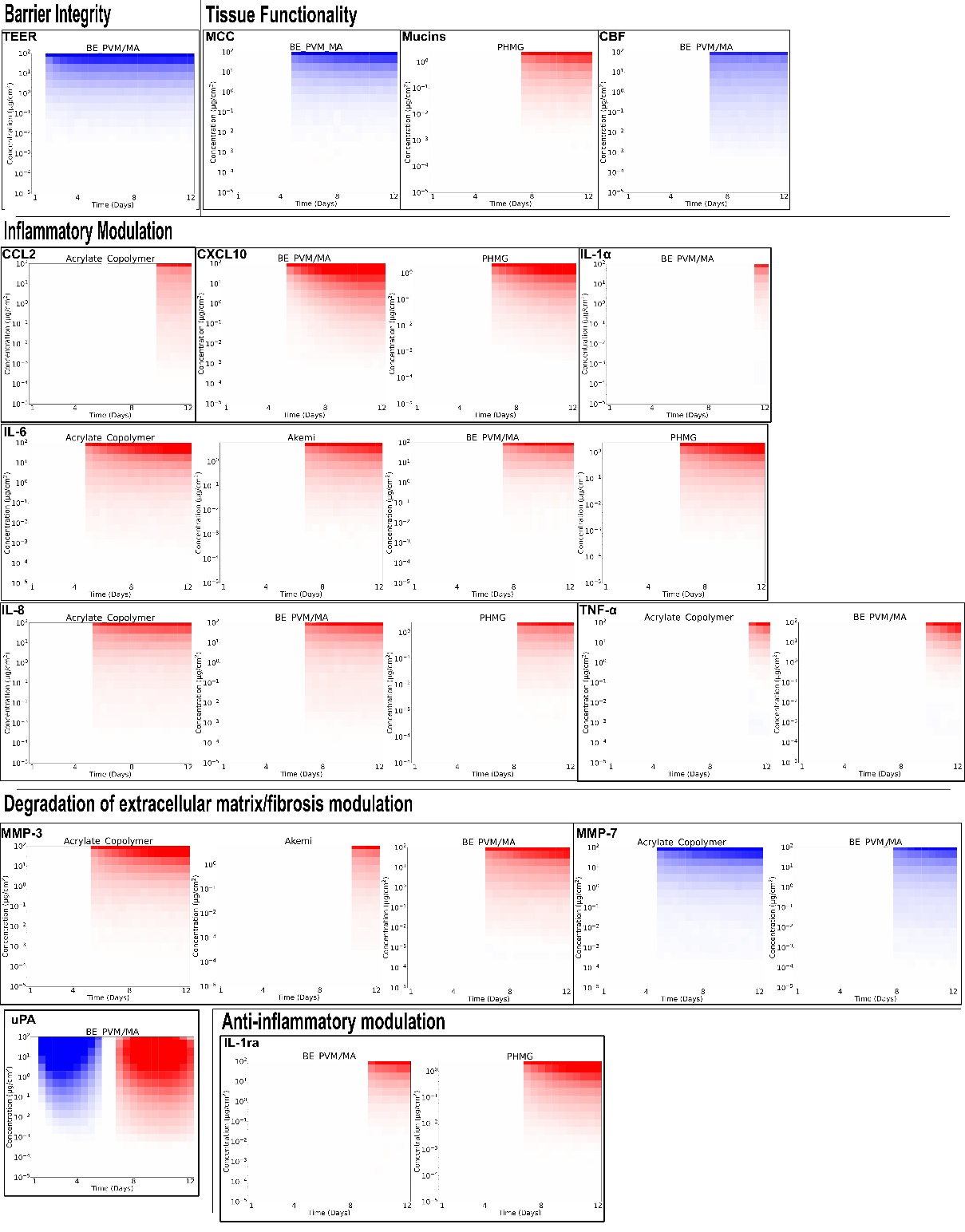
**

**Supplementary Figure S2.** Reference material-induced changes in the upper respiratory MucilAir™-HF tissues. Tissues were exposed to the chemicals, through aerosol (^Ar^) or via apical (^AL^)/basal liquid (^BL^) application, once a day repeatedly for 30 min/day and/or 6 h/day on 12 consecutive days with 3-4 different concentrations. On days 1, 4, 8 and 12, the following parameters were evaluated: barrier integrity through transepithelial electrical resistance (TEER) measurements analysis; tissue functionality by the assessment of cilia beating frequency (CBF), mucociliary clearance (MCC) and mucin secretion; inflammatory, degradation of extracellular matrix/fibrosis, and anti-inflammatory responses through cytokine and chemokine quantification. Figure shows only chemicals that triggered changes in the investigated parameters when compared to unexposed tissues groups. Blue and red colours represent down- or up-regulation, whereas the colour variation shows the intensity of such effects. For all substances, a comparison between the concentration- and time-dependent response analysis and corresponding readout data can be found in the Suppl. Data 4.

**
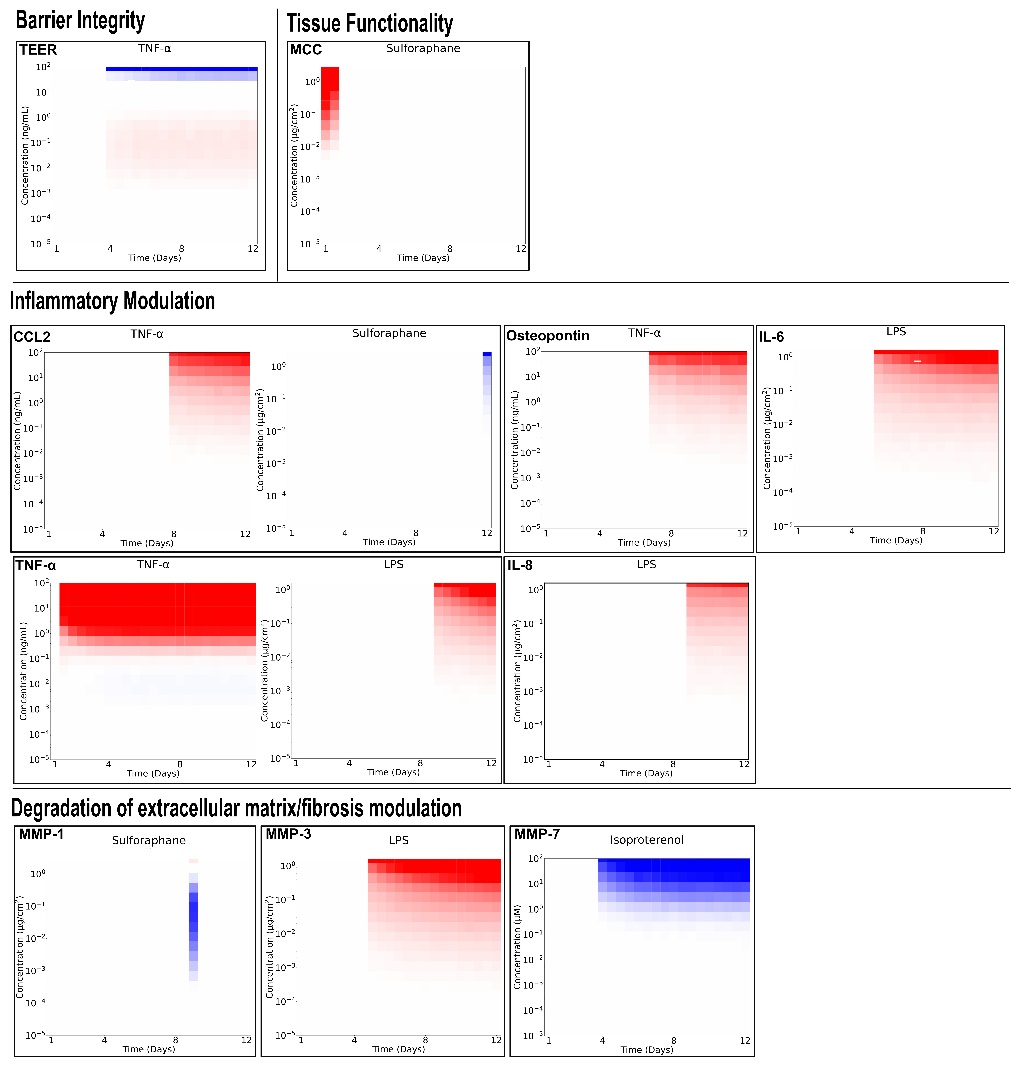
**

**Supplementary Figure S3.** Presence of mucin-producing goblet cells in MucilAir™-HF tissues was investigated through Muc5AC staining by immunohistochemistry analysis (n=4 tissues/substance concentration). The images are representative of the findings observed by a board-certified pathologist.


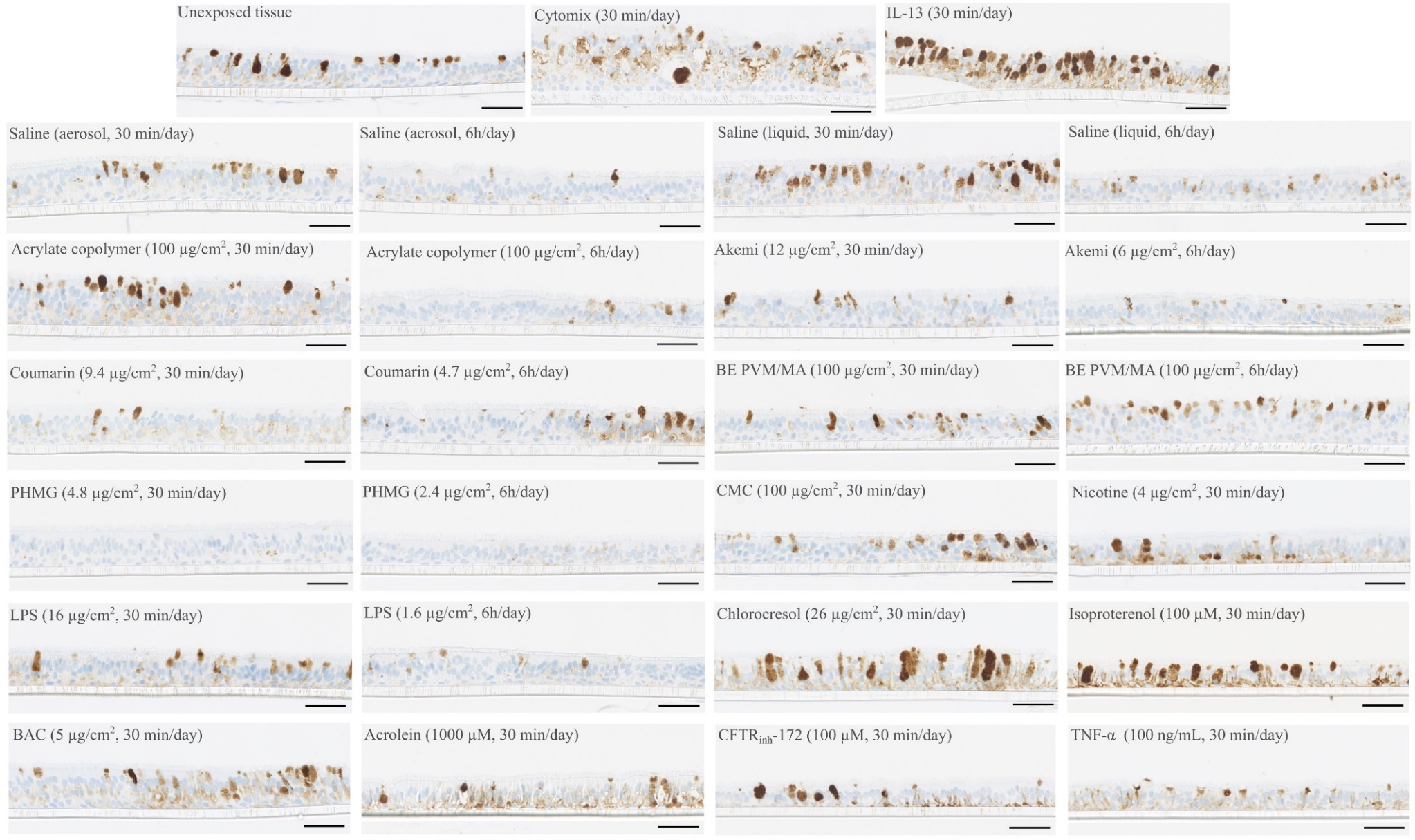


**Supplementary Figure S4.** Heatmap indicating the correspondence between different latent variables (LVs) and the prior biological knowledge used in the PLIER analysis. Depth of shading indicates the strength of association.


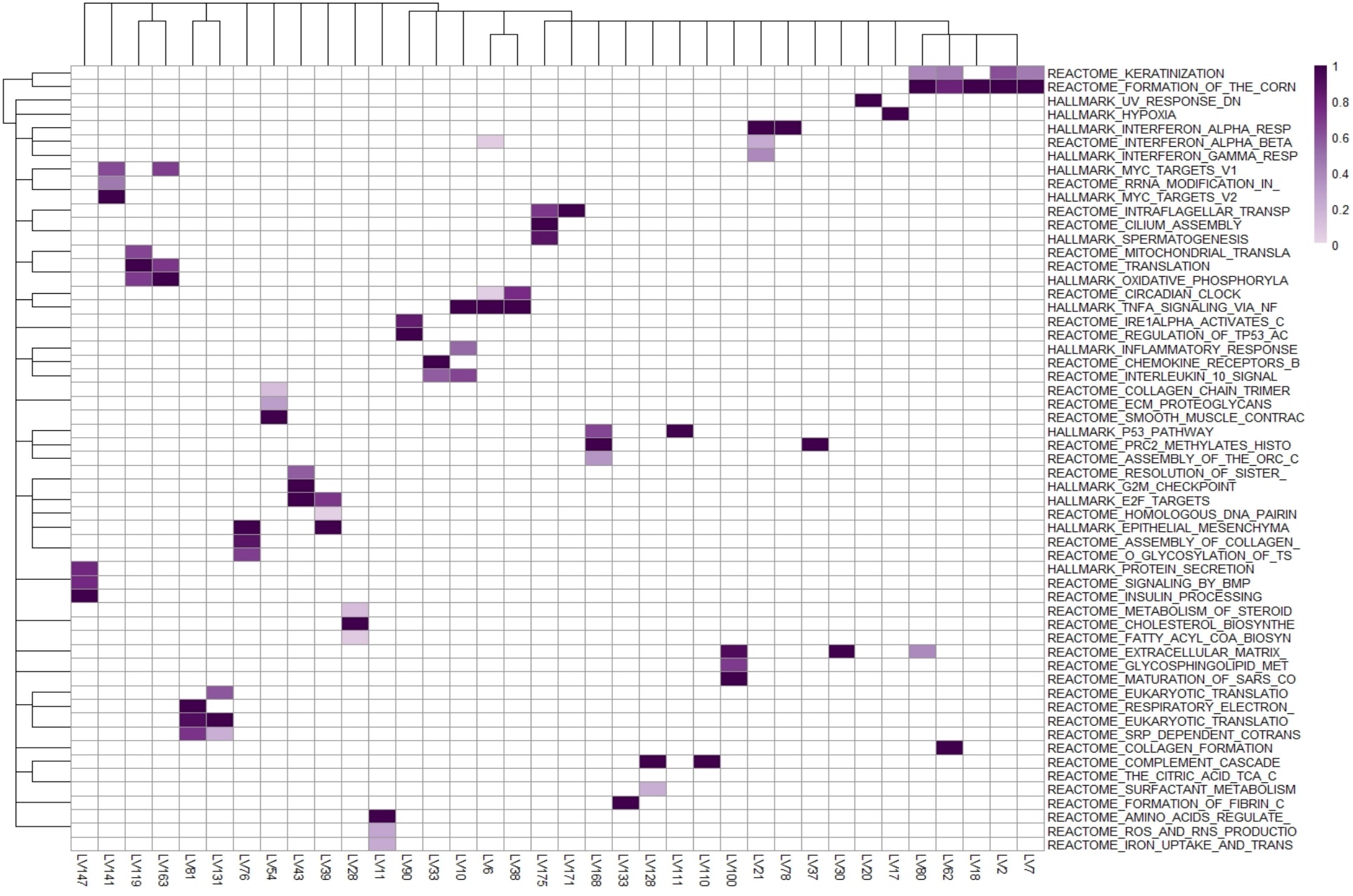


**Supplementary Figure S5.** Benchmark chemical-exposure scenarios with bioactivity exposure ratio (BER) point estimates. Overview of the most sensitive, minimum obtained PoD and mean upper and lower respiratory tract exposure estimates for each chemical according to its related exposure scenario. **(A)** Upper MucilAir™ respiratory tract model. **(B)** Lower EpiAveolar™ respiratory tract model.


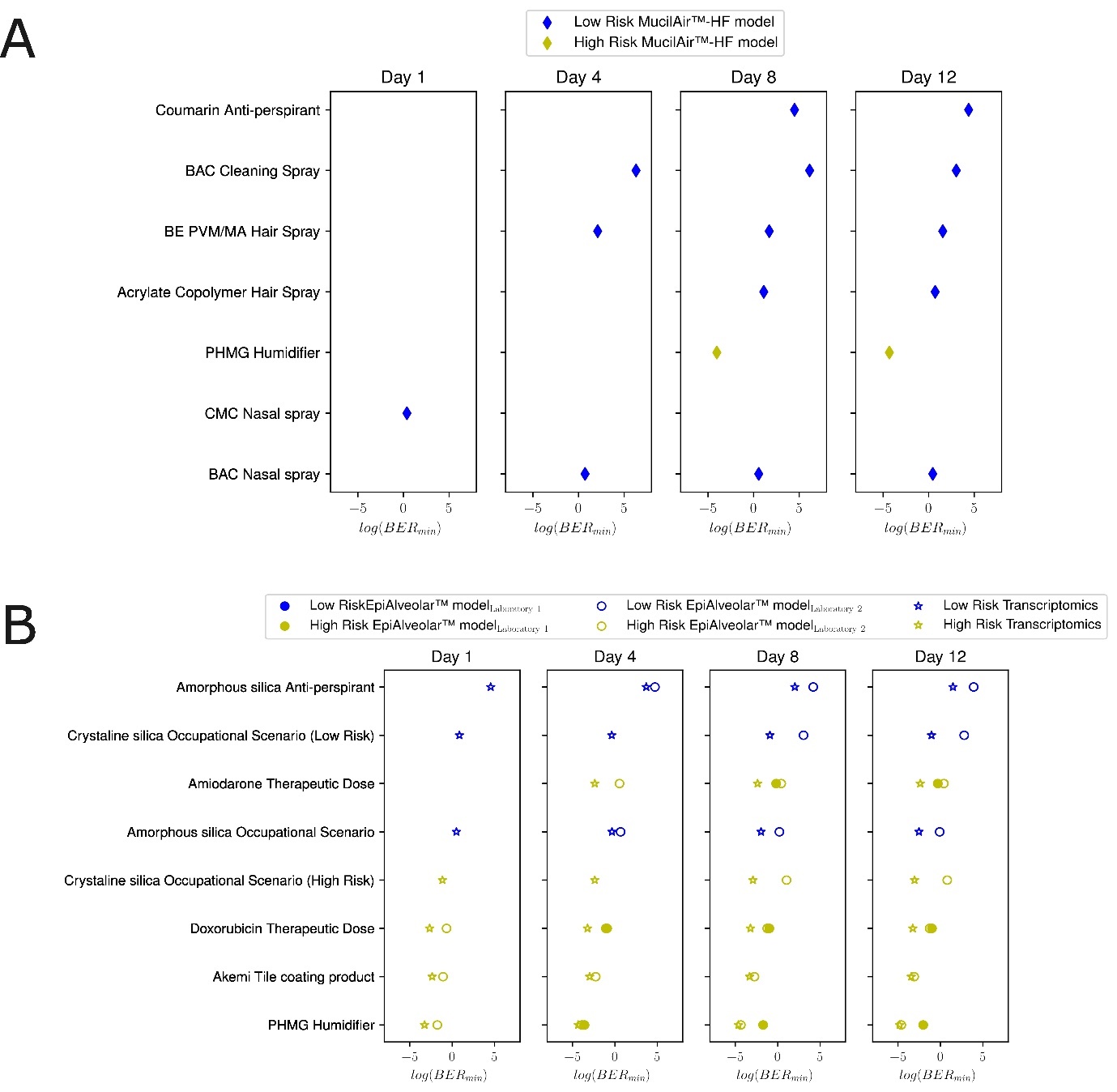


**Supplementary Figure S6.** Overview of PoDs related to tissue barrier integrity, tissue functionality, modulation of cytokines/chemokines, and/or mitotoxicity evaluations, and associated mean upper and lower respiratory tract exposure estimates for each chemical.


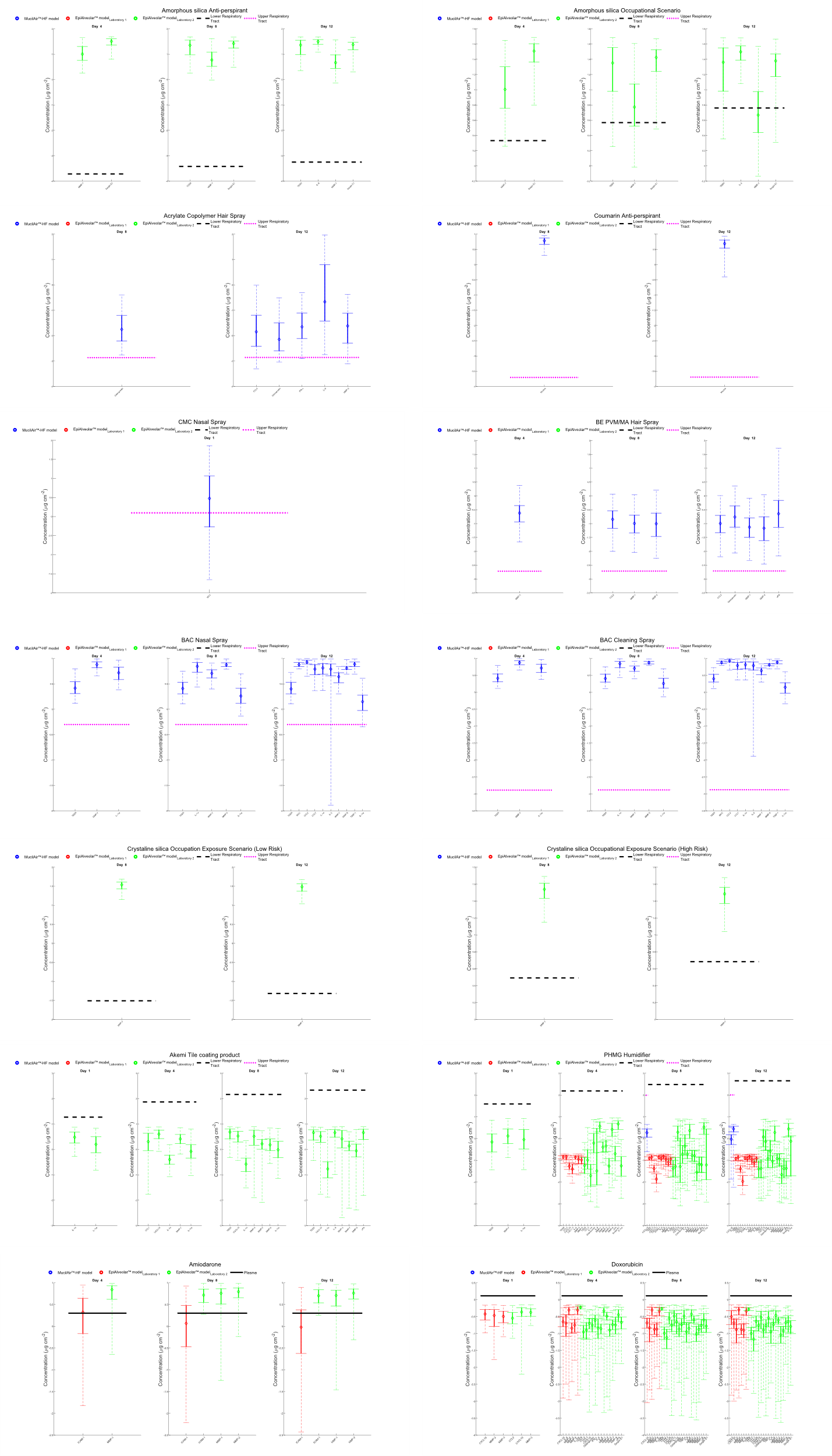


**Supplementary Figure S7.** Overview of EpiAlveolar™ transcriptomics PoDs and associated mean upper and lower respiratory tract exposure estimates for each chemical.


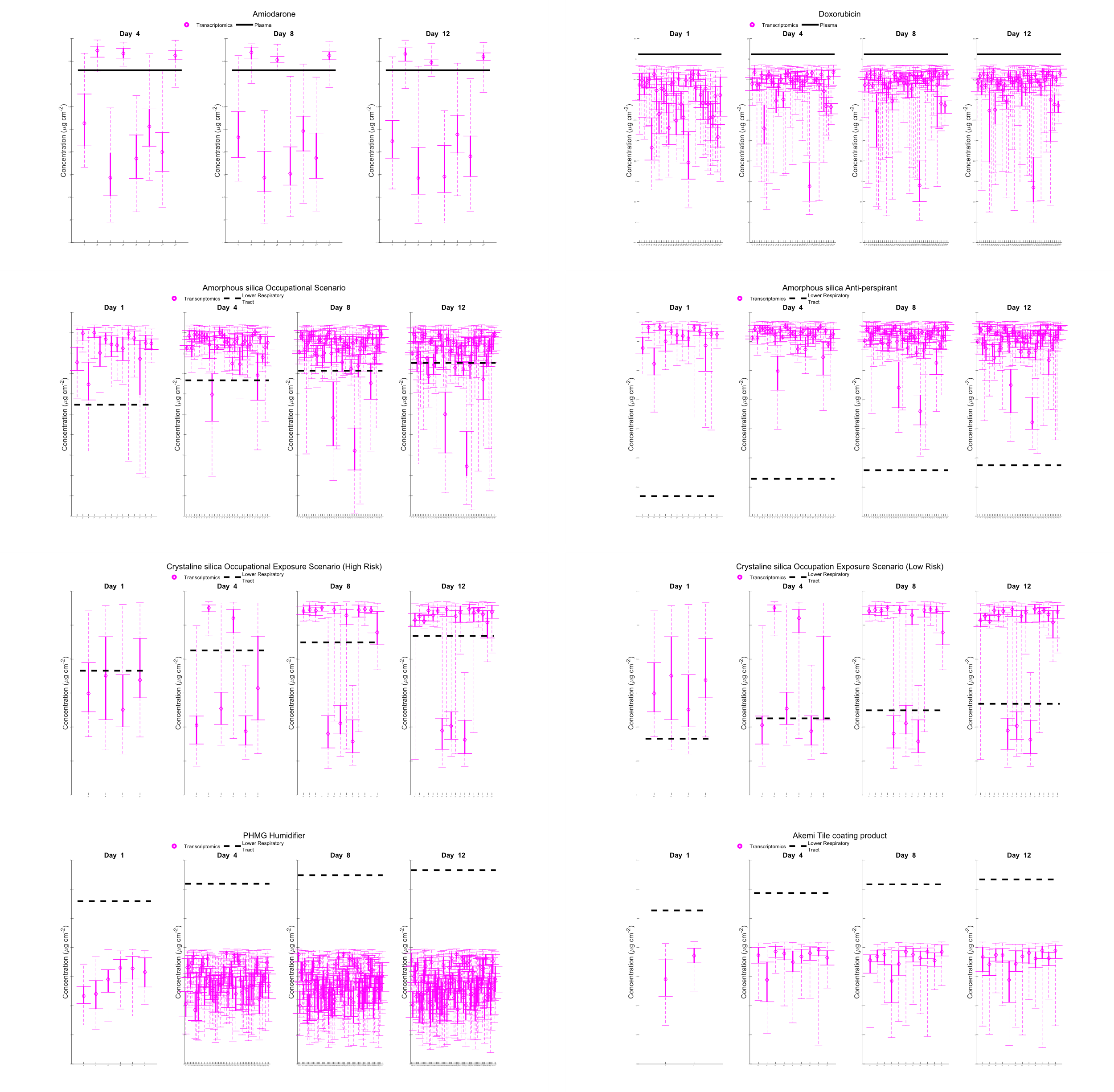


# REFERENCES

Aggarwal, B. B., et al., 2012. Historical perspectives on tumor necrosis factor and its superfamily: 25 years later, a golden journey. Blood. 119**,** 651-665.

Balharry, D., et al., 2008. An in vitro approach to assess the toxicity of inhaled tobacco smoke components: Nicotine, cadmium, formaldehyde and urethane. Toxicology. 244**,** 66-76.

Comer, D. M., et al., 2014. Inflammatory and cytotoxic effects of acrolein, nicotine, acetylaldehyde and cigarette smoke extract on human nasal epithelial cells. BMC Pulmonary Medicine. 14**,** 32.

Deshmukh, H. S., et al., 2005. Metalloproteinases Mediate Mucin 5AC Expression by Epidermal Growth Factor Receptor Activation. American Journal of Respiratory and Critical Care Medicine. 171**,** 305-314.

Dwivedi, A. M., et al., 2015. Acute effects of acrolein in human volunteers during controlled exposure. Inhalation Toxicology. 27**,** 810-821.

Dwivedi, A. M., et al., 2018. Inflammatory effects of acrolein, crotonaldehyde and hexanal vapors on human primary bronchial epithelial cells cultured at air-liquid interface. Toxicology in Vitro. 46**,** 219-228.

Farhana, A., Khan, Y. S., 2022. Biochemistry, Lipopolysaccharide. [Updated 2022 Apr 21]. In: StatPearls [Internet]. Treasure Island (FL): StatPearls Publishing; 2023 Jan-. Available from: <https://www.ncbi.nlm.nih.gov/books/NBK554414/>.

Gerloff, J., et al., 2017. Inflammatory Response and Barrier Dysfunction by Different e-Cigarette Flavoring Chemicals Identified by Gas Chromatography-Mass Spectrometry in e-Liquids and e-Vapors on Human Lung Epithelial Cells and Fibroblasts. Appl In Vitro Toxicol. 3**,** 28-40.

Guo, L., et al., 2012. Mitochondrial reactive oxygen species mediates nicotine-induced hypoxia-inducible factor-1α expression in human non-small cell lung cancer cells. Biochimica et Biophysica Acta (BBA) - Molecular Basis of Disease. 1822**,** 852-861.

Jiao, Z., et al., 2017. Sulforaphane increases Nrf2 expression and protects alveolar epithelial cells against injury caused by cigarette smoke extract. Mol Med Rep. 16**,** 1241-1247.

JOUNEAU, S., et al., 2011. EMMPRIN (CD147) regulation of MMP-9 in bronchial epithelial cells in COPD. Respirology. 16**,** 705-712.

Kwong, K. Y. C., et al., 2004. Expression of transforming growth factor beta (TGF-β1) in human epithelial alveolar cells: a pro-inflammatory mediator independent pathway. Life Sciences. 74**,** 2941-2957.

Lee, S. U., et al., 2016. Verproside inhibits TNF-α-induced MUC5AC expression through suppression of the TNF-α/NF-κB pathway in human airway epithelial cells. Cytokine. 77**,** 168-175.

Li, Q., et al., 2010. Nicotine suppresses inflammatory factors in HBE16 airway epithelial cells after exposure to cigarette smoke extract and lipopolysaccharide. Translational Research. 156**,** 326-334.

Lindén, A., 1996. Increased interleukin-8 release by beta-adrenoceptor activation in human transformed bronchial epithelial cells. Br J Pharmacol. 119**,** 402-6.

Matsuyama, N., et al., 2018. The dopamine D1 receptor is expressed and induces CREB phosphorylation and MUC5AC expression in human airway epithelium. Respiratory Research. 19**,** 53.

McKay, S., et al., 2000. Tumor Necrosis Factor- α Enhances mRNA Expression and Secretion of Interleukin-6 in Cultured Human Airway Smooth Muscle Cells. American Journal of Respiratory Cell and Molecular Biology. 23**,** 103-111.

Nilsson, H. E., et al., 2010. CFTR and tight junctions in cultured bronchial epithelial cells. Experimental and Molecular Pathology. 88**,** 118-127.

Oehme, S., et al., 2015. Agonist-induced β2-adrenoceptor desensitization and downregulation enhance pro-inflammatory cytokine release in human bronchial epithelial cells. Pulmonary Pharmacology & Therapeutics. 30**,** 110-120.

Perez, A., et al., 2007. CFTR inhibition mimics the cystic fibrosis inflammatory profile. American Journal of Physiology-Lung Cellular and Molecular Physiology. 292**,** L383-L395.

Reddy, S., et al., 2002. Identification of glutathione modifications by cigarette smoke. Free Radical Biology and Medicine. 33**,** 1490-1498.

Sthijns, M. M. J. P. E., et al., 2014. Adaptation to acrolein through upregulating the protection by glutathione in human bronchial epithelial cells: The materialization of the hormesis concept. Biochemical and Biophysical Research Communications. 446**,** 1029-1034.

Szymanski, M. W., Singh, D. P., 2023. Isoproterenol. StatPearls. StatPearls Publishing

Copyright © 2023, StatPearls Publishing LLC., Treasure Island (FL).

Uchenna Agu, R., et al., 1999. Effects of Pharmaceutical Compounds on Ciliary Beating in Human Nasal Epithelial Cells: A Comparative Study of Cell Culture Models. Pharmaceutical Research. 16**,** 1380-1385.

Wu, Y.-L., et al., 2010. Glucosamine regulation of LPS-mediated inflammation in human bronchial epithelial cells. European Journal of Pharmacology. 635**,** 219-226.

Xiong, R., et al., 2018. Evaluating Mode of Action of Acrolein Toxicity in an In Vitro Human Airway Tissue Model. Toxicological Sciences. 166**,** 451-464.

Ye, S.-F., et al., 2009. ROS and NF-κB are involved in upregulation of IL-8 in A549 cells exposed to multi-walled carbon nanotubes. Biochemical and Biophysical Research Communications. 379**,** 643-648.

Zeng, X., et al., 2021. Sulforaphane suppresses lipopolysaccharide- and Pam3CysSerLys4-mediated inflammation in chronic obstructive pulmonary disease via toll-like receptors. FEBS Open Bio. 11**,** 1313-1321.
