# Supplementary material for: Evaluation of a non-animal toolbox informed by adverse outcome pathways for human inhalation safety
Source: Front Toxicol. 2025 Feb 21;7:1426132. doi: 10.3389/ftox.2025.1426132 (PMC11885506; doi:10.3389/ftox.2025.1426132)
Supplement: Supplementary file 4 [file DataSheet2.docx]

**Supplementary Material S2**

Evaluation of a non-animal toolbox informed by adverse outcome pathways (AOPs) for human inhalation safety

**Renato Ivan de Ávila**^1^**, Iris Müller**^1^**, Hugh Barlow**^1^**, Alistair Mark Middleton**^1^**, Mathura Theiventhran**^1^**, Danilo Basili**^1^**, Anthony M. Bowden**^1^**, Ouarda Saib**^1^**, Patrik Engi**^1^**, Tymoteusz Pietrenko**^1^**, Joanne Wallace^2^, Bernadett Boda^3^, Samuel Constant^3^, Holger Peter Behrsing^4^, Vivek Patel^4^, Maria Teresa Baltazar**^1*^

^1^Safety, Environmental and Regulatory Science (SERS), Unilever, Colworth Science Park, Sharnbrook, Bedfordshire, United Kingdom.

^2^Charles River Laboratories, Edinburgh, United Kingdom.

^3^Epithelix Sarl, Plan-les-Outes, Switzerland.

^4^Respiratory Toxicology Program, Institute for In Vitro Sciences, Inc., Gaithersburg, MD, United States.

**^*^Correspondence:**Corresponding Author: Maria Baltazar, PhD, maria.baltazar@unilever.com

#### 1. Materials

All reagents used for MucilAir™-HF and EpiAlveolar™ systems were obtained from Epithelix Sàrl (Geneva, Switzerland) and MatTek Corporation (Ashland, MA, USA), respectively. Some test materials were purchased from Sigma-Aldrich (St. Louis, MO, USA), whereas others acquired from the following providers: R,S-sulforaphane (LKT Laboratories, Inc., St. Paul, MN, USA), Crystalline silica (Min-U-Sil® 5, US Silica Co., Berkely Springs, WV, USA), AKEMI® anti-fleck super (Wessling Laboratorien GmbH, Altenberg, Germany), Acrylate copolymer (ACUDYNE™ DHR copolymer, Dow, Midland, MI, USA), butyl ester of poly(methyl vinyl ether-alt-maleic acid monoethyl ester) copolymer (Gantrez™ ES425 copolymer, Ashland LCC, Kidderminster, UK), Polyhexamethyleneguanidine phosphate (BOC Sciences, London, UK), recombinant human TNF-α (carrier-free) (Biolegend, San Diego, CA, USA), and IL-13 (ProSpec-Tany TechnoGene Ltd., East Brunswick, NJ, USA). HEPES, CaCl2 were obtained from Dutscher (Bernolsheim, France), AppliChem GmbH (Darmstadt, Germany). Physiological saline (0.9% NaCl, w/v) buffered saline solution, phosphate-buffered saline (PBS) and fetal calf serum (FCS) were obtained from Baxter (Deerfield, IL, USA), Bichsel (Unterseen, Switzerland), Pan Biotech (Aidenbach, Germany) and Bioconcept (Allschwil, Switzerland), respectively. Tetramethylbenzidine substrate, MitoTracker® Red FM dye, hematoxylin II, Tris EDTA, and Triton X-100 were acquired from BD Biosciences (Franklin Lakes, NJ, USA), Roche (Basel, Switzerland), Thermo Fisher Scientific (Waltham, MA, USA), ScyTek (West Logan, UT, USA), and Fluka, Merck KGaA (Darmstadt, Germany), respectively. GSH/GSSG-Glo™ Assay kit was obtained from Promega (Southampton, UK). Hematoxylin, eosin, and Harris’ hematoxylin reagents were purchased from Pioneer Research Chemicals Ltd (Colchester, UK). Weigert’s iron hematoxylin were prepared using hematoxylin, ethanol, ferric chloride and 32% hydrochloric acid that were acquired from Thermo Fisher, Hayman (Witham, UK), SLS (Nottingham, UK) and Acros Organics (Waltham, MA, USA), respectively. If not indicated otherwise, primary and secondary antibodies used for immunohistochemistry analyses as well as antibodies binding kits were purchased from Invitrogen (Waltham, MA, USA), Jackson Immuno Research Laboratories, Inc. (West Grove, PA, USA), Cell Signaling (Danvers, MA, USA), Leica Biosystems (Nussloch, Germany), BioSB (Santa Barbara, CA, USA), Agilent (Santa Clara, CA, USA), Abcam (Cambridge, UK), Sino Biological (Eschborn, Germany), Dako (Santa Clara, CA, USA), (Leica Microsystems Ltd, Milton Keynes, UK), and Hoffmann-La Roche Ltd (Basel, Switzerland). QIAzol lysis reagent and RNeasy 96 QIAcube HT kit were purchased from Quiagen (Hilden, Germany), and DNA-free kit from Thermo Fischer. RNA ScreenTape reagents and TruSeq™ Stranded mRNA kit were purchased from Agilent and Illumina (San Diego, CA, USA), respectively. All other materials were acquired from Sigma-Aldrich.

#### 2. Lung clearance estimates

Clearance estimates for both the upper and lower airways were performed using the ICRP human respiratory model (ICRP, 1994), as described below.

The upper airway clearance was modelled by coupled ordinary differential equations which simulated the transport of chemicals up the pulmonary generations. For the upper 17 generations, where the first generation is the trachea, the rate of change of the mass $m_{i}$ in generation was given by

$$\frac{dm_{i}}{dt}=\dot{\delta}-\lambda m_{i}-2\frac{dm_{i+1}}{dt}$$

Where $\dot{\delta}$ is the depositied aerosolised mass, $\lambda$ is the mass clearance rate due to the transport of mucus up the respiratory system. This was set by the mucus velocity in the trachea and determined for each generation using conservation of mass. The final term in this equation corresponds to the movement of material from lower generations to upper generations, with a factor of 2 coming from the symetric lung model used.

In the alveolar generations of the lung, (generation 17-23), the ICRP multi exponential model was used. The factors used in this model are based on data obtained for the clearance of radioactive dust and is therefore likely to underestimate the rate of clearance for the chemicals considered in this study.

The rate of clearance of the alveolar generations is set a three compartment model

$$\frac{dm_{i}}{dt}=\frac{dm_{1,i}}{dt}+\frac{dm_{2,i}}{dt}+\frac{dm_{3,i}}{dt}$$

$$\frac{dm_{1,i}}{dt}+\lambda_{1}m_{1,i}=0.3 \dot{\delta}_{i}$$

$$\frac{dm_{2,i}}{dt}+\lambda_{2}m_{2,i}=0.6 \dot{\delta}_{i}$$

$$\frac{dm_{3,i}}{dt}+(\lambda_{3}+\lambda_{lymph})m_{3,i}=0.1 \dot{\delta}_{i}$$

$$\frac{dm_{lymph,i}}{dt}+\lambda_{lymph}m_{2,i}=0$$

The local concentration was then derived from dividing the calculated mass in each generation by the corresponding surface area.

3. Test material solution preparation

3.1 MucilAir™-HF model

To prepare the stock solutions to perform the test material exposures, buffered saline solution (0.9 % NaCl, 10 mM HEPES, 1.25 mM CaCl2) was used to dilute water soluble compounds for aerosol or apical liquid exposure, while compounds for basal liquid exposure were diluted in culture medium. Basal medium was supplemented with either dimethyl sulfoxide (DMSO, 1 %, v/v) or solution containing PBS (0.2 %, v/v) and fetal bovine serum (FCS, 0.02 %, v/v) for CFTRinh-172 and TNF-α, respectively. Solutions of the compounds were prepared once for the two weeks of exposure, aliquoted and stored at 4°C for polymers and -20°C for other materials.

3.2 EpiAlveolar™ model

In Laboratory 1, materials were prepared immediately prior to use in the experiments. Stock solutions were prepared using designated vehicles for each test item. Amiodarone, Doxorubicin, and LPS had serial stock solutions (1000x) made in DMSO that were then spiked into culture medium to obtain the final (respective) concentrations for apical or basal exposures. For aerosol exposure, Crystalline silica and PHMG exposure solutions, and Akemi (as a well-mixed exposure emulsion) were made in 0.00225% saline in ultrapure water. Similarly, Sulforaphane and Amorphous silica (in 0.9% saline) solutions were made and used for aerosol exposure.

In Laboratory 2, PHMG and Akemi stock solutions were prepared daily before performing chemical exposures, whereas Crystalline and Amorphous silica suspensions were prepared via sonication for 1h and mixed by vortex prior to nebulization process; the same suspensions, stored at room temperature, were used for the whole 12-day experiments. Sulforaphane, Doxorubicin and Amiodarone stock solutions (in DMSO, except for Sulforaphane which used ultrapure water) were filter sterilized and aliquots were stored at -20°C; fresh stock vials were used each day to avoid unnecessary freeze-thaws. After dissolving, LPS stock solution was stored at 4°C. The final DMSO concentration in the tissue-based assays did not exceed 0.1% (v/v in culture medium) for those test items in which DMSO was used as the vehicle; whereas where dilute saline is specified, this was a 1% (v/v) dilution in physiological saline (0.9%, w/v) and the final saline concentration in the tissue-based assays was 0.009%.

4. Histology and immunohistochemistry analyses in the lower airway EpiAlveolar™ model

4.1 Methods

4.1.1 Laboratory 1

Tissues from a set of one donor batch were transferred to a 12-well plate containing 750 µL of 10% phosphate buffered formalin for fixation. Also, 250 µL were added to the apical side of the tissues which were storage for a minimum of 24 h. Afterwards, tissues were transferred to containers containing 70% ethanol and sent to a histology facility (StageBio, Mount Jackson, VA, USA) for additional processing. Each tissue was carefully removed from the insert and bisected. Two bisected halves were paraffin-embedded in a single block with the cut face upward. Paraffin blocks were sectioned to obtain 5 µm sections that were mounted to glass slides for the analyses. The slides were then dewaxed in xylene and stained with hematoxylin and eosin (H&E).

Immunohistochemistry staining was performed onboard the Leica BOND RX autostainer for aquaporin 5 detection, while manual methods using slide racks were used for pro-surfactant, vimentin and pan-cytokeratin analyses.

For aquaporin 5 detection, slides were deparaffinized with Bond Dewax (Leica Biosystems) and heat-induced epitope retrieval with Epitope Retrieval 2 (Leica Biosystems) for 20 min at 100oC. Primary antibody, rabbit anti-aquaporin 5 at 1:200 (PA599403, Invitrogen), was incubated for 60 min at room temperature. Peroxidase conjugated Donkey anti-Rabbit (711-035-152, Jackson Immuno Research Laboratories, Inc.) was used as the secondary. Antibody binding was visualized through incubation with SignalStain DAB kit (Cell Signaling).

For other biomarkers, slides were deparaffinized in xylene and heat-induced epitope retrieval was performed by incubating at 60°C for 16 h in Diva Decloaker (Biocare, Pacheco, CA, USA) for pro-surfactant; at 98°C and 95°C for 20 min and 15 sec, respectively, in 0.015% citraconic anhydride solution for pan-cytokeratin; or at 110°C and 95°C for 10 min and 15 s, respectively, in Tris EDTA pH 9.0 for vimentin detection. One of the following primary antibodies were incubated for 60 min at room temperature: rabbit anti-pro-surfactant C at 1:500 (AB3786, Sigma-Aldrich); mouse anti-pan-cytokeratin AE1/E3 at 1:250 (BSB 5433, BioSB); or mouse anti-vimentin at 1:1000 (BSB 6027, BioSB). Rabbit on Canine HRP (Biocare) was used as the secondary antibody for anti-pro-surfactant C, whereas Mouse on Canine HRP (Biocare) was used as the secondary for pan-cytokeratin and vimentin analysis. Antibodies binding was visualized through incubation with SignalStain DAB kit. For staining control, including aquaporin 5 and pro-surfactant C, human normal lung slides were used.

Histology and immunohistochemistry evaluations were conducted by a board-certified pathologist through assessment of the following parameters using a semi-quantitative scoring system (Table 1): percentage of epithelial cell loss, percentage of cilia, metaplastic changes, apoptotic/necrotic bodies, loss of pseudo-stratification, and reactive nuclear changes.

**Suppl. Material S2, Table 1.** Histology and immunohistochemistry evaluation criteria used for Laboratory 1 to analyse EpiAlveolar™ tissues.

| **Parameters**^a^ | **Scaling key** |
| --- | --- |
| %epithelial cell loss and/or cilia | Determined/estimated after scanning the entire surface of the airway sections using low and high magnifications |
| Metaplastic changes, e.g. squamous cell metaplasia | 0 = no metaplastic change  1 = early changes  2 = intermediate  3 = well-formed squamous morphology |
| Apoptotic/necrotic bodies | 0 = no to rare  1 = 3-10  2 = 11-20  3 = >20 |
| Loss of pseudo-stratification | 0 = no appreciable loss  1 = up to 25% loss  2 = 25%-50% loss  3 = 50-75% loss  4 = 75-100% loss |
| Reactive nuclear changes, e.g. enlargement, irregular contours, prominent nucleoli and anisonucleosis | 0 = no appreciable change  1 = mild  2 = moderate  3 = marked |

^a^All assessments were based on the pathologist`s judgement.

*4.1.2 Laboratory 2*

Tissues were kept in 10% phosphate buffered formalin at 4°C for up to 48 h. Samples were then fixed in 70% ethanol and two strips of membrane were trimmed from each sample, processed, and paraffin-embedded to be cut at 5 µm thick sections and mounted to glass slides. The slides were then dewaxed in xylene, hydrated using graded industrial methylated spirit (70-100%) and stained with H&E for light microscope analysis.

For immunohistochemistry analysis, tissue sections-containing microscope slides were obtained and processed using a Discovery Ultra Staining System (Roche), according to manufacturer’s instructions, except for anti-CD68 staining that was manually performed. The slides were incubated at 60°C for 8 min and then deparaffinized at 69°C for 24 min. After that, slides were incubated with inhibitor CM or peroxidase blocking solution (for anti-CD68 staining) for 8 min at room temperature. The slides were then incubated at room temperature with the following primary monoclonal antibodies, following manufacturer’s instructions: anti-α smooth muscle actin (αSMA) at 1:500 (Leica Microsystems Ltd), anti-CD68 at 1:3000 for macrophages (11192-T24, Sino Biological), anti-pro-surfactant C protein at 1:1000 (AB3786, Sigma-Aldrich), anti-caspase-3 at 1:10000 (AB4051, Abcam), anti-aquaporin 5 (31661, Invitrogen), anti-pan-cytokeratin for epithelial cells (760-2595, Roche), and anti-vimentin for mesenchymal cells (790-2917, Roche). Incubation with secondary antibodies were performed with the following reagents, in accordance with the manufacturer’s instructions: OmniMap anti-mouse HRP 760-4310 (Roche), EnVision System HRP anti-rabbit K4003 (Agilent, Santa Clara), or UltraMap anti-rabbit 760-4315 (Roche). Antibody binding was visualized through the incubation with Discovery Purple Roche 253-4857 (Roche), Liquid DAB substrate K3468 (Agilent), 3.3 diaminobenzidine (DAB) 7604304 (Roche) or Liquid DAB+Substrate Chromogen System K3468 (Dako), followed by pre- and post-counterstaining with hematoxylin II and Bluing reagent, respectively, and dehydration process. The staining of the investigated biomarkers was validated using human formalin fixed lung or tonsil tissues (data not shown). Aquaporin 5 staining was the only one not possible to validate. Thus, no further staining of the EpiAveolar™ samples was attempted for this biomarker.

Mucus and collagen visualization were also performed with Periodic Acid-Schiff (PAS) and Puchtler’s Picro-Sirius Red (PSR) staining, respectively. In brief, dewaxed and rehydrated slides were incubated with 1% periodic acid (10 min) and Schiff’s reagent (10 min), being a 10-min washing process in water performed after each process. Slides were then counterstained with Harris’ hematoxylin followed by nuclei staining with hematoxylin used regressively, placing the slides into alkaline Scott’s tap water solution (40 mM NaHCO3 and 170 mM MgSO₄), and dehydration process. For collagen assessment, dewaxed and rehydrated slides were stained in Weigert’s iron hematoxylin (5 min) followed by washing process and then placed into picro-sirius red solution. After 1 h, slides were washed in acidified water and dehydrated.

A board-certified pathologist evaluated the changes (epithelial thinning, separation/detachment areas, necrosis, collagen, and presence of apoptotic cells, myofibroblasts, and type II pneumocytes) using a semi-quantitative scoring system (Table 2).

**Suppl. Material S2, Table 2.** Histology and immunohistochemistry evaluation criteria used for Laboratory 2 to analyse EpiAlveolar™ tissues.

| **Parameters**^a^ | |
| --- | --- |
| Epithelial thinning | Areas of 2 or less cell layers think |
| Cell degeneration | Presence of pyknotic nuclei and karyorrhectic debris |
| Separation/detachment areas | Epithelial cells and fibroblasts had separated from each other or from the underlying membrane |
| Necrosis | Cell hyper-eosinophilia, complete loss of nuclear detail with retention of cell outlines; coagulative necrosis |
| Presence of collagen | extracellular deposits of intensely red material in PSR stains |
| Presence of apoptotic cells, myofibroblasts, and type II pneumocytes | Caspase-3, αSMA, or pro-surfactant C positive cells, respectively |

^a^Scores from 0 to 4 was used to classify severity grade as absent, minimal, mild, moderate or marked.

*5.2 Results*

*5.2.1 Laboratory 1*

To evaluate the quality of EpiAlveolar™ tissues over the 12-day experimental period, histological and immunohistochemistry assessments for detection of pan-cytokeratin, vimentin, aquaporin 5, and pro-surfactant C were performed by Laboratory 1 (Figure 1 and Table 3).

It was observed that the cell viability of tissues was not significantly impacted, except for occasional apoptotic bodies. Overall, cell viability was ~90% on days 0, 1 and 4. On days 8 and 12, tissues showed mostly intact viable epithelium, however overall cellularity appeared to be decreased with flattening/thinning of cell layers.

Regarding the cell morphology, the basal layer cells exhibited somewhat spindle (flat squamous-like) morphology, while the superficial cells were mostly cuboidal. There was significant dyscohesion and separation of cell layers. Rare cytoplasmic vacuolations were observed. On days 8 and 12, tissues appeared to exhibit less dyscohesion and maintained attachment with the membrane with some loss of pseudostratification. The cells of both basal and superficial layers were flatter when compared to days 0, 1, and 4. Occasional cells with cilia were noted (overall less than 5-10%). Some cells appear to exhibit early metaplastic squamous cell-like morphology.

In addition, immunohistochemical assessment showed that pan-cytokeratin, a broad-spectrum epithelial marker, was diffusely and strongly expressed by all the cells above the membrane (and endothelial below the membrane were found to be negative). Vimentin, typically expressed by the mesenchymal cells, was diffusely and strongly expressed by the basal cells and the thin layer of cell under the membrane; the superficial epithelial cells were negative. Moreover, 20-25% of basally located cells showed dispersed granular cytoplasmic staining for aquaporin 5, which was not the expected pattern of staining and most likely represent non-specific false positive staining. However, expected pattern of pro-surfactant C staining (i.e. strong cytoplasmic staining of type II pneumocytes) was not observed. There was weak cytoplasmic staining in a small subset of cells (~5%) which was best interpreted as negative. The surface epithelial cells, which were supposed to represent pneumocytes, were negative.

**Suppl. Material S2, Figure 1.** Histology and immunohistochemistry assessments of EpiAlveolar™ tissues over the 12-day period performed by Laboratory 1. Tissues were incubated at standard culture conditions (37±1°C in a humidified atmosphere of 5% CO_2_ in air). Three different tissue samples were collected on days 0, 1, 4, 8, and 12 for hematoxylin and eosin (H&E) staining and immunohistochemistry detection of cytokeratin, vimentin, aquaporin 5, and pro-surfactant C. The images are representative of the findings observed by a board-certified pathologist over the 12-day period. Purple, green, and blue bars represent 20, 50, and 100 µm, respectively.


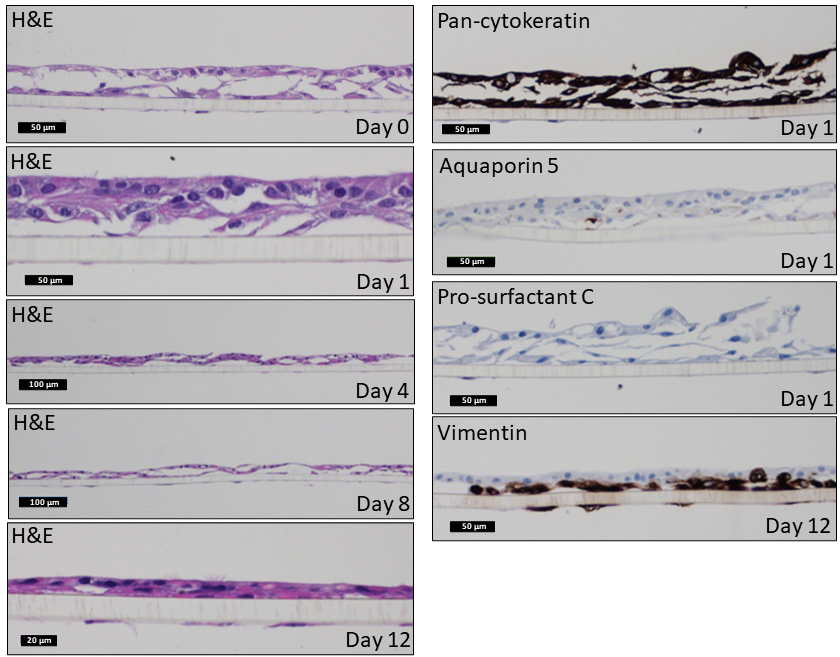


**Suppl. Material S2, Table 3.** Quality analysis of EpiAlveolar™ tissues over the 12-day experimental period through histological and immunohistochemistry assessments performed by Laboratory 1.

| Day | Epithelial cell loss (%) | Cilia (%) | Intensity scores | | | | Positive cells (%) | | | |
| --- | --- | --- | --- | --- | --- | --- | --- | --- | --- | --- |
|  |  |  | Metaplastic changes | Apoptotic /necrotic bodies | Loss of Pseudo-stratification | Reactive nuclear changes | Pan-cytokeratin | Vimentin | Aquaporin 5 | Pro-surfactant C |
| 0 | 5 | 10 | 1 | 1 | 0 | 0 | 100 | 50 | 20 | 5 |
|  | 5 | 10 | 1 | 0 | 0 | 0 | 100 | 50 | 20 | 5 |
|  | 5 | 10 | 1 | 0 | 0 | 0 | 100 | 50 | 20 | 5 |
| 1 | 15 | 10 | 1 | 1 | 1 | 0 | 100 | 50 | 20 | 5 |
|  | 5 | 10 | 1 | 1 | 0 | 0 | 100 | 50 | 20 | 5 |
|  | 10 | 10 | 1 | 1 | 0 | 0 | 100 | 50 | 20 | 5 |
| 4 | 10 | 10 | 1 | 1 | 0 | 0 | 100 | 50 | 20 | 5 |
|  | 10 | 10 | 1 | 1 | 0 | 0 | 100 | 50 | 20 | 5 |
|  | 10 | 10 | 1 | 1 | 0 | 0 | 100 | 50 | 25 | 5 |
| 8 | 20 | 5 | 1 | 1 | 1 | 0 | 100 | 50 | 25 | 5 |
|  | 20 | 5 | 1 | 1 | 1 | 0 | 100 | 50 | 20 | 5 |
|  | 20 | 5 | 1 | 1 | 2 | 0 | 100 | 50 | 20 | 5 |
| 12 | 20 | 5 | 1 | 0 | 1 | 0 | 100 | 50 | 20 | 5 |
|  | 20 | 10 | 1 | 0 | 1 | 0 | 100 | 50 | 25 | 5 |
|  | 30 | 5 | 1 | 1 | 2 | 0 | 100 | 50 | 25 | 5 |

Analyses were performed for three different EpiAlveolar™ tissues.

5.2.2 Laboratory 2

In Laboratory 2, a quality control of the tissues (Figure 2 and Table 4) as well as analysis of the changes induced by the chemicals were also performed by histology evaluation and immunohistochemistry assessments for detection of CD68, caspase-3, αSMA, pro-surfactant C, pan-cytokeratin, and/or vimentin (Table 5).

On days 0 and 1, control and aerosol vehicle groups showed a layer of pan-cytokeratin positive polygonal epithelial cells, 3-4 deep arranged upon a 1-2 cell deep vimentin positive spindle shaped fibroblast layer directly adherent to the underlying membrane. Within the fibroblast layer, there were low numbers of αSMA positive myofibroblasts whilst the surface epithelial cell layer contained low numbers of cells with short cilia suggestive of airway epithelial cells and cells with foamy PAS positive cytoplasm suggestive of airway mucous cells; both cell types are not normally present in the alveoli and may have originated from the small airways or represent aberrant differentiation of the alveolar epithelial population. Occasional small round aggregates of PSR material were observed within the deep epithelial cell layers. The microscopic appearance of these aggregates was not typical of collagen deposits *in vivo* in which collagen is deposited as initially loosely arranged then dense and organised linear fibres. However, it may represent aberrant collagen deposition associated with the *in vitro* nature of the EpiAveolar™ model.

Within both the epithelial and fibroblast layers, but most common in the former, there were rare degenerate cells, a proportion of which stained positively for caspase-3 expression. Low numbers of pseudocystic spaces containing PAS positive material and cell debris were also presented. Rare, weakly CD68 positive macrophages were present predominantly in the upper epithelial cell layers. Vimentin positive endothelial cells were evident on the underside of the membrane. There was often artefactual intercellular separation of epithelial cells from each other or epithelial cells from the fibroblasts layer, but the fibroblast layer generally remained adherent to the membrane with only occasional minimal detachment seen.

On day 4, the tissues morphology was acceptable, however it was observed early signs degeneration with a minor increase in thinning of the epithelial layers, and a minor increase in numbers of degenerate cells. On day 8, more pronounced degeneration with increased thinning, higher numbers of degenerate cells and greater areas of membrane detachment were found. On day 12, tissues exhibited pronounced thinning and often pronounced detachment from the membrane with remaining cells exhibiting a spindle shape phenotype. Interestingly, as culture progressed to 12 days, there was no increase in cells expressing caspase-3, αSMA or PSR positive collagen deposits, suggesting that extended culture results in degeneration of the epithelium but not a shift to a pro-fibrotic phenotype. Also, it is worth to mention that no pro-surfactant C staining was evident over 12-day period, suggesting that either type II pneumocytes were not present or that they de-differentiated sufficiently that they stopped producing pro-surfactant C. Moreover, rare CD68 positive macrophages seen in the early days, were not found later.

Regarding the tissues exposed to the test materials, changes were not observed for Amiodarone and LPS when compared to vehicle and control groups. On the other hand, Akemi exposure was associated with concentration-dependent damage (epithelial thinning and separation/detachment); necrosis was the most severe change seen on day 1 at the highest concentration group and in all concentrations groups from day 4; on day 12, the highest concentration group showed marked necrosis with loss of significant amounts of the tissue.

Crystalline silica was associated with a minor increase in epithelial thinning on day 4 at the highest concentration (50 µg/cm^2^) which was also observed for all other concentrations on day 8; minor increase in membrane detachment was observed on day 8 in tissues exposed at 5 and 50 µg/cm^2^.

Amorphous silica at 5 and 50 µg/cm^2^ was associated with a minor increase in epithelial thinning from day 1; greater separation/detachment was also observed in such tissues at day 12 as well as minor necrosis at 0.01 µg/cm^2^ on days 8 and 12 and marked necrosis at 50 µg/cm^2^ on day 12.

PHMG was associated with greater epithelial thinning and membrane detachment on day 1; by day 4, marked thinning was found, equivalent or greater than that seen on day 12; from day 4, degenerate cell numbers and necrosis were also higher compared to vehicle group at the highest concentrations (0.9 and 9.4 µg/cm^2^).

No appreciable differences were induced by Doxorubicin on day 1; however, a minor increase in epithelial thinning and a minor increase in the numbers of degenerate cells were observed by day 4. On days 8 and 12, there was greater epithelial thinning; although no clear concentration response was evident, this finding was more pronounced at 0.72 µM of Doxorubicin.

Therefore, in general, the concentration related changes induced by those chemicals ranging from increased cell degeneration, separation/detachment, multifocal thinning (Crystalline silica, Amorphous silica, and Doxorubicin) and with more severe injury locally extensive cell death/necrosis (PHMG and Akemi).

**Suppl. Material S2, Figure 2.** Histology and immunohistochemistry assessments of EpiAlveolar™ tissues over the 12-day period performed by Laboratory 2. Tissues were incubated at standard culture conditions (37±1°C in a humidified atmosphere of 5% CO_2_ in air). Two different tissue samples were collected on days 0, 1, 4, 8, and 12 for hematoxylin and eosin (H&E), Periodic Acid-Schiff (PAS), or Puchtler’s Picro-Sirius Red (PSR) staining and immunohistochemistry detection of cytokeratin, vimentin, CD68, αSMA, caspase-3, and pro-surfactant C. The images were taken using 40× objective and are representative of the findings observed by a board-certified pathologist over the 12-day period. Black asterisk shows intercellular separation. The arrows represent the following: red: degenerate epithelial cell; blue: endothelial cells; green: polygonal epithelial cells; black: spindle shaped fibroblasts; white: PAS positive mucin within surface epithelia cell cytoplasm; yellow: pseudocystic spaces containing PAS positive mucin; orange: surface cells with cilia/microvilli; pink: extracellular aggregates of PSR positive collagen; brown: apoptotic epithelial cells expressing caspase-3.


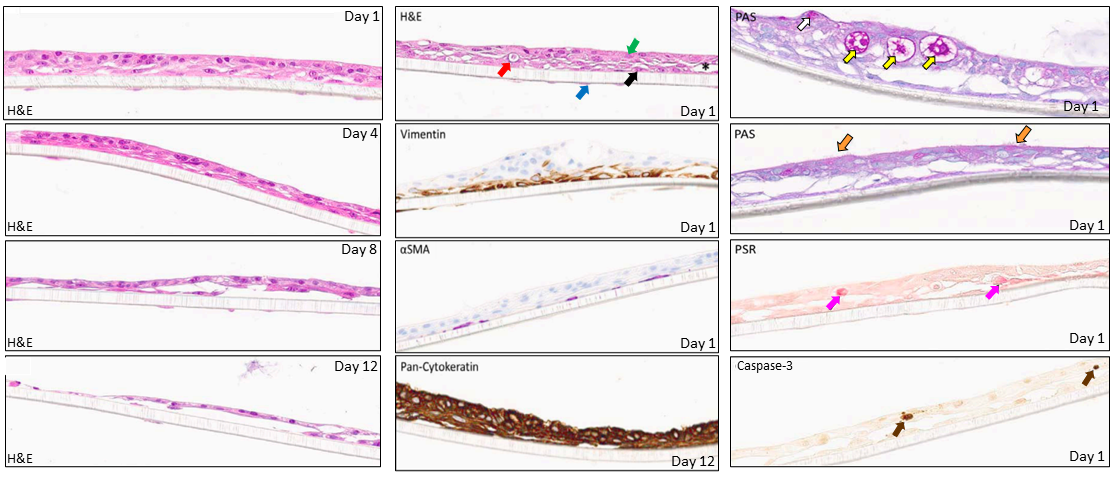


**Suppl. Material S2, Table 4.** Quality analysis of EpiAlveolar™ tissues over the 12-day experimental period through histological and immunohistochemistry assessments performed by Laboratory 2.

| Day | Epithelial thinning | Cell degeneration | Separation/ detachment | Necrosis | Collagen (PSR+ material) | Caspase-3 | Pro-surfactant C | αSMA | Mucin containing cells (PAS+ material) | Cytokeratin | CD68 | Vimentin |
| --- | --- | --- | --- | --- | --- | --- | --- | --- | --- | --- | --- | --- |
| 0 | 0 | 0 | 1 | 0 | 1 | 1 | 0 | 1 | 2 |  |  |  |
|  | 1 | 1 | 1 | 0 | 1 | 1 | 0 | 1 |  |  |  |  |
|  | 0 | 1 | 1 | 0 | 1 | 1 |  | 2 |  |  |  |  |
|  | 0 | 1 | 1 | 0 | 1 | 1 |  | 1 |  |  |  |  |
| 1 | 0 | 0 | 0 | 0 | 1 | 2 | 0 | 2 | 2 | 4 | 2 | 2 |
|  | 0 | 1 | 1 | 0 | 1 | 1 |  | 2 |  |  |  |  |
|  | 0 | 0 | 1 | 0 | 1 | 1 |  | 1 |  |  |  |  |
| 4 | 0 | 1 | 1 | 0 | 0 | 1 | 0 | 1 | 1 | 4 | 1 | 2 |
|  | 1 | 1 | 2 | 0 | 1 | 1 |  | 1 |  |  |  |  |
|  | 1 | 1 | 2 | 0 | 1 | 0 |  | 1 |  |  |  |  |
| 8 | 2 | 1 | 1 | 0 | 0 | 1 | 0 | 0 | 0 | 4 | 0 | 1 |
|  | 2 | 2 | 3 | 0 | 0 | 1 |  | 0 |  |  |  |  |
|  | 1 | 2 | 2 | 0 | 0 | 0 |  | 0 |  |  |  |  |
| 12 | 3 | 3 | 3 | 0 | 0 | 1 | 0 | 0 | 0 | 3 | 0 | 1 |
|  | 4 | 2 | 4 | 0 | 0 | 1 |  | 0 |  |  |  |  |
|  | 3 | 2 | 2 | 0 | 0 | 0 |  | - |  |  |  |  |

Data are present as scores findings using a semi-quantitative scoring system (scores from 0 to 4 to classify severity grade as absent, minimal, mild, moderate or marked).

Two tissue samples from each group were collected on day 0, 1, 4, 8 or 12 for histology and immunohistochemistry assessments.

Grey shading: no staining performed due to absence of findings in previous samples.

Symbol - means insufficient tissue for examination.

**Suppl. Material S2, Table 5.**  Histological and immunohistochemistry assessments performed by Laboratory 2 in EpiAlveolar™ tissues exposed to chemicals.

| Parameter | Aerosol Vehicle | | | | | Akemi (µg/cm^2^) | | | | Crystalline silica (µg/cm^2^) | | | | Amorphous silica (µg/cm^2^) | | | | PHMG (µg/cm^2^) | | | | LPS (mg/mL) | | | | Amiodarone (µM) | | | | Doxorubicin (µM) | | | |
| --- | --- | --- | --- | --- | --- | --- | --- | --- | --- | --- | --- | --- | --- | --- | --- | --- | --- | --- | --- | --- | --- | --- | --- | --- | --- | --- | --- | --- | --- | --- | --- | --- | --- |
|  |  |  |  |  |  | 0.3 | 0.8 | 1.6 | 8 | 0.01 | 1 | 5 | 50 | 0.01 | 1 | 5 | 50 | 0.1 | 0.5 | 0.9 | 9.4 | 0.01 | 0.1 | 1 | 10 | 0.01 | 0.1 | 1 | 10 | 0.18 | 0.36 | 0.72 | 0.36 |
| Epithelial thinning | 0 | 0 | 0 | 0 | 0 | 0 | 0 | 0 | 2 | 1 | 1 | 1 | 1 | 0 | 0 | 2 | 3 | 2 | 2 | 2 | 3 | 0 | 0 | 0 | 0 | 0 | 1 | 1 | 0 | 0 | 0 | 0 | 0 |
|  | 1 | 1 | 2 | 1 | 1 | 2 | 1 | 1 | 2 | 2 | 2 | 2 | 3 | 1 | 1 | 2 | 3 | 4 | 4 | 4 | 4 | 1 | 1 | 1 | 1 | 2 | 2 | 2 | 1 | 2 | 2 | 2 | 1 |
|  | 2 | 3 | 3 | 2 | 2 | 1 | 2 | 2 | 2 | 4 | 4 | 4 | 4 | 2 | 1 | 2 | 3 | 4 | 4 | 4 | 4 | 1 | 1 | 2 | 2 | 3 | 3 | 3 | 3 | 3 | 3 | 4 | 4 |
|  | 3 | 4 | 4 | 3 | 2 | 3 | 3 | 2 | - | 4 | 4 | 4 | - | 3 | 3 | 4 | 4 | 4 | 4 | 4 | 4 | 1 | 2 | 2 | 3 | 3 | 4 | 3 | 3 | 3 | 3 | 4 | 4 |
| Cell Degeneration | 2 | 1 | 1 | 1 | 0 | 0 | 0 | 0 | 1 | 1 | 1 | 2 | 1 | 1 | 1 | 1 | 1 | 2 | 2 | 2 | 3 | 1 | 0 | 1 | 1 | 1 | 1 | 1 | 1 | 2 | 2 | 2 | 2 |
|  | 2 | 2 | 2 | 1 | 1 | 2 | 2 | 2 | 1 | 2 | 2 | 2 | 3 | 2 | 1 | 1 | 2 | 2 | 2 | 3 | 4 | 2 | 1 | 1 | 1 | 2 | 1 | 2 | 2 | 2 | 3 | 2 | 2 |
|  | 3 | 2 | 2 | 1 | 1 | 1 | 1 | 1 | 2 | 2 | 2 | 1 | 1 | 1 | 1 | 1 | 1 | 2 | 1 | 4 | 4 | 2 | 2 | 2 | 1 | 2 | 1 | 1 | 1 | 1 | 1 | 3 | 1 |
|  | 1 | 2 | 1 | 2 | 1 | 3 | 1 | 1 | - | 1 | 1 | 1 | - | 1 | 1 | 1 | 1 | 4 | 1 | 4 | 4 | 1 | 1 | 2 | 2 | 2 | 1 | 1 | 1 | 1 | 1 | 2 | 1 |
| Separation/ detachment | 0 | 1 | 1 | 1 | 1 | 0 | 1 | 0 | 1 | 0 | 0 | 1 | 2 | 0 | 1 | 1 | 1 | 0 | 1 | 1 | 1 | 0 | 1 | 0 | 1 | 1 | 2 | 1 | 1 | 0 | 0 | 0 | 0 |
|  | 1 | 2 | 2 | 1 | 1 | 2 | 2 | 2 | 1 | 1 | 2 | 2 | 3 | 1 | 1 | 2 | 2 | 2 | 2 | 1 | 3 | 0 | 1 | 1 | 1 | 2 | 2 | 2 | 1 | 0 | 0 | 0 | 1 |
|  | 1 | 2 | 2 | 3 | 2 | 1 | 2 | 1 | 2 | 3 | 2 | 3 | 4 | 1 | 1 | 2 | 3 | 4 | 4 | 4 | 4 | 1 | 1 | 2 | 2 | 3 | 3 | 3 | 3 | 1 | 1 | 3 | 1 |
|  | 1 | 2 | 1 | 3 | 2 | 3 | 3 | 2 | 4 | 3 | 2 | 2 | - | 2 | 1 | 4 | 4 | 4 | 4 | 4 | 4 | 1 | 2 | 2 | 3 | 3 | 3 | 3 | 3 | 1 | 1 | 2 | 1 |
| Necrosis | 0 | 0 | 0 | 0 | 0 | 0 | 0 | 0 | 2 | 0 | 0 | 0 | 0 | 0 | 0 | 0 | 0 | 0 | 0 | 0 | 0 | 0 | 0 | 0 | 0 | 0 | 0 | 0 | 0 | 0 | 0 | 0 | 0 |
|  | 0 | 0 | 0 | 0 | 0 | 1 | 2 | 1 | 2 | 0 | 0 | 0 | 0 | 0 | 0 | 0 | 0 | 0 | 0 | 1 | 4 | 0 | 0 | 0 | 0 | 0 | 0 | 0 | 0 | 0 | 0 | 0 | 0 |
|  | 0 | 0 | 0 | 0 | 0 | 0 | 0 | 1 | 3 | 0 | 0 | 0 | 0 | 1 | 0 | 0 | 0 | 0 | 0 | 4 | 4 | 0 | 0 | 0 | 0 | 0 | 0 | 0 | 0 | 0 | 0 | 0 | 0 |
|  | 0 | 0 | 0 | 0 | 0 | 1 | 1 | 2 | 4 | 0 | 0 | 0 | 0 | 1 | 0 | 0 | 4 | 4 | 0 | 4 | 4 | 0 | 0 | 0 | 0 | 0 | 0 | 0 | 0 | 0 | 0 | 0 | 0 |
| Collagen (PSR+ material) | 1 | 1 | 1 | 1 | 1 | 1 | 1 | 1 | 1 | 1 | 1 | 1 | 1 | 1 | 0 | 1 | 0 | 1 | 1 | 1 | 1 | 1 | 1 | 1 | 1 | 2 | 1 | 1 | 1 | 1 | 2 | 1 | 1 |
|  | 1 | 1 | 1 | 1 | 0 | 0 | 0 | 0 | 0 | 0 | 1 | 0 | 0 | 1 | 0 | 0 | 1 | 1 | 1 | 1 | 1 | 0 | 0 | 0 | 1 | 1 | 0 | 1 | 0 | 1 | 1 | 1 | 1 |
|  | 1 | 1 | 0 | 0 | 0 | 0 | 0 | 1 | 0 | 0 | 0 | 0 | 0 | 0 | 0 | 0 | 0 | 0 | 0 | 0 | 1 | 0 | 0 | 0 | 1 | 0 | 0 | 0 | 0 | 1 | 1 | 0 | - |
|  | 1 | 0 | 1 | 0 | 0 | 0 | 0 | 0 | 0 | 0 | 0 | 0 | 0 | 0 | 0 | 0 | 0 | 0 | 0 | 0 | 1 | 0 | 0 | 0 | 0 | 0 | 0 | 0 | 0 | 1 | 1 | 1 | 0 |
| Caspase-3 | 2 | 1 | 1 | 1 | 1 | 1 | 1 | 1 | 0 | 1 | 1 | 2 | 2 | 1 | 1 | 1 | 1 | 1 | 1 | 2 | 2 | 0 | 0 | 1 | 0 | 1 | 1 | 1 | 1 | 2 | 1 | 2 | 2 |
|  | 1 | 1 | 1 | 1 | 0 | 0 | 0 | - | 0 | 1 | 1 | 1 | 2 | 1 | 1 | 1 | 0 | 0 | 0 | - | 0 | - | - | 1 | 0 | 0 | 0 | 0 | 1 | 2 | 2 | 2 | 2 |
|  | 1 | 1 | 1 | 0 | 0 | 0 | 1 | 1 | 0 | 0 | 0 | 1 | 1 | 1 | 0 | 0 | 0 | - | - | - | 0 | 0 | 0 | 0 | 0 | 0 | 0 | 0 | 1 | 0 | - | 1 | - |
|  | 1 | 0 | - | 1 | 0 | 0 | - | 0 | 0 | 0 | - | 0 | - | - | 0 | - | - | - | 0 | - | 0 | 0 | 0 | 0 | 0 | 1 | - | 0 | 1 | 1 | 1 | 1 | - |
| Pro-surfactant C | 0 | 0 | 0 |  |  |  |  |  |  | 0 | 0 | 0 | 0 | 0 | 0 | 0 | 0 | 0 | 0 | 0 | 0 |  |  |  |  |  |  |  |  | 0 | 0 | 0 | 0 |
|  | 0 | 0 | 0 |  |  |  |  |  |  | 0 | 0 | 0 | 0 | 0 | 0 | 0 | 0 | 0 | 0 | 0 | 0 |  |  |  |  |  |  |  |  | 0 | 0 | 0 | 0 |
|  | 0 | 0 | 0 |  |  |  |  |  |  | 0 | 0 | 0 | 0 | 0 | 0 | 0 | 0 | - |  |  |  |  |  |  |  |  |  |  |  | 0 | 0 | 0 | - |
|  | 0 | 0 | 0 |  |  |  |  |  |  | - | 0 | 0 | - | 1 | 0 | 0 | - |  |  |  |  |  |  |  |  |  |  |  |  | 0 | 0 | 0 | - |
| αSMA | 2 | 2 | 2 | 2 | - | 2 | - | 1 | 1 | 1 | 2 | 3 | 2 | 2 | 2 | 1 | 2 | 1 | 1 | 1 | 1 | 2 | 1 | 2 | 2 | 2 | 2 | 2 | 1 | 2 | 2 | 1 | 1 |
|  | 1 | 1 | 1 | 1 | 1 | 1 | - | 0 | - | 1 | 0 | 1 | 1 | 1 | 1 | 1 | 1 | 1 | - | - | - | - | 1 | 1 | 1 | 1 | 0 | 0 | 1 | 0 | 0 | 0 | 0 |
|  | 1 | 0 | 0 | 0 | - | 1 | 1 | 0 | - | 0 | 1 | 0 | 0 | 0 | 0 | 1 | 0 | 1 | - | - | 0 | 0 | 0 | 0 | 0 | 1 | 1 | 0 | 0 | 0 | 0 | 0 | 0 |
|  | 0 | 0 | 0 | 0 | - | - | 1 | 0 | - | 0 | 0 | 0 | 0 | 1 | 0 | 0 | - | 0 | - | - | 0 | 0 | 0 | 0 | - | 0 | 0 | 0 | 0 | 0 | 0 | 0 | - |

Data are present as scores findings using a semi-quantitative scoring system (scores from 0 to 4 to classify severity grade as absent, minimal, mild, moderate or marked).

Two tissue samples from each group were collected on day 0, 1, 4, 8 or 12 for histology and immunohistochemistry assessments.

Grey shading: no staining performed due to absence of findings in previous samples.

Symbol - means insufficient tissue for examination.

# REFERENCES

ICRP, Human Respiratory Tract Model for Radiological Protection ICRP Publication 66. Ann. ICRP, Vol. 24. ICRP, 1994.
