# Supplementary material for: Evaluation of a non-animal toolbox informed by adverse outcome pathways for human inhalation safety
Source: Front Toxicol. 2025 Feb 21;7:1426132. doi: 10.3389/ftox.2025.1426132 (PMC11885506; doi:10.3389/ftox.2025.1426132)
Supplement: Supplementary file 1 [file DataSheet4.docx]

**Supplementary Material S4**

Evaluation of a non-animal toolbox informed by adverse outcome pathways (AOPs) for human inhalation safety

**Renato Ivan de Ávila**^1^**, Iris Müller**^1^**, Hugh Barlow**^1^**, Alistair Mark Middleton**^1^**, Mathura Theiventhran**^1^**, Danilo Basili**^1^**, Anthony M. Bowden**^1^**, Ouarda Saib**^1^**, Patrik Engi**^1^**, Tymoteusz Pietrenko**^1^**, Joanne Wallace^2^, Bernadett Boda^3^, Samuel Constant^3^, Holger Peter Behrsing^4^, Vivek Patel^4^, Maria Teresa Baltazar**^1*^

^1^Safety, Environmental and Regulatory Science (SERS), Unilever, Colworth Science Park, Sharnbrook, Bedfordshire, United Kingdom.

^2^Charles River Laboratories, Edinburgh, United Kingdom.

^3^Epithelix Sarl, Plan-les-Outes, Switzerland.

^4^Respiratory Toxicology Program, Institute for In Vitro Sciences, Inc., Gaithersburg, MD, United States.

**^*^Correspondence:**
Corresponding Author: Maria Baltazar, PhD, maria.baltazar@unilever.com

This section shows concentration- and time-dependent response data for MucilAir™-HF and EpiAlveolar™ models for the test materials (see tables below) performed using a nonlinear state space model. Regarding the graphs presented here, pink and black dashed lines represent 95% credible range of control and credible range of mean response, respectively; green dots are the data points, whereas the red shading corresponds to the probability distribution of the mean response; X and Y axes represent time (days) and fold change in relation to vehicle control, respectively.

**Suppl. Material S4, Table 1.** Test materials used in the upper respiratory toxicity testing using MucilAir™-HF systems.

| **Items** | **CAS no.** | **Abbreviation** | **Exposure time/day** | **Tested concentrations** |
| --- | --- | --- | --- | --- |
| AKEMI^®^ anti-fleck super | - | Akemi | 30 min | 1, 6, 12 µg/cm^2^ |
|  |  |  | 6 h | 6 µg/cm^2^ |
| ACUDYNE™ DHR copolymer | - | Acrylate copolymer | 30 min | 0.1, 10, 100 µg/cm^2^ |
|  |  |  | 6 h | 100 µg/cm^2^ |
| Butyl ester of poly(methyl vinyl ether-alt-maleic acid monoethyl ester) copolymer | - | BE PVM/MA | 30 min | 0.1, 10, 100 µg/cm^2^ |
|  |  |  | 6 h | 100 µg/cm^2^ |
| Carboxymethylcellulose sodium salt | [9004-32-4](https://www.sigmaaldrich.com/GB/en/search/9004-32-4?focus=products&page=1&perpage=30&sort=relevance&term=9004-32-4&type=cas_number) | CMC | 30 min | 2.5, 5, 10, 100 µg/cm^2^ |
| Coumarin | 91-64-5 | Coumarin | 30 min | 0.5, 4.7, 9.4 µg/cm^2^ |
|  |  |  | 6 h | 4.7 µg/cm^2^ |
| Polyhexamethyleneguanidine phosphate | 89697-78-9 | PHMG | 30 min | 0.8, 2.4, 4.8  µg/cm^2^ |
|  |  |  | 6 h | 2.4 µg/cm^2^ |
| Nicotine | 54-11-5 | Nicotine | 30 min | 0.04, 0.4, 4 µg/cm^2^ |
| Lipopolysaccharide from *Pseudomonas aeruginosa* 10 | - | LPS | 30 min | 0.2, 1.6, 16 µg/cm^2^ |
|  |  |  | 6 h | 1.6 µg/cm^2^ |
| Benzalkonium chloride | 63449-41-2 | BAC | 30 min | 0.1, 0.5, 5, 10 µg/cm^2^ |
| Acrolein | 107-02-8 | Acrolein | 30 min | 500, 750, 1000 µM |
| 4-Chloro-3-methylphenol | 59-50-7 | Chlorocresol | 30 min | 1.3, 2.6, 26 µg/cm^2^ |
| Isoproterenol hydrochloride | 5984-95-2 | Isoproterenol | 30 min | 1, 50, 100 µM |
| CFTR_inh_-172 | 307510-92-5 | CFTR_inh_-172 | 30 min | 1, 10, 100 µM |
| Recombinant human TNF-α (carrier-free) | - | TNF-α | 30 min | 10, 50, 100 ng/mL |
| R,S-Sulforaphane | 4478-93-7 | Sulforaphane | 30 min | 0.1, 1.4, 2.9 µg/cm^2^ |
|  |  |  | 6 h | 1.4 µg/cm^2^ |
| Dimethyloctadecyl[3-(trimethoxysilyl)propyl] ammonium chloride | 27668-52-6 | DMOD | 30 min | 0.1, 10, 50 µg/cm^2^ |
|  |  |  | 6 h | 10 µg/cm^2^ |
| Proprietary chemical 1 | - | SEAC1 | 30 min | 0.1, 10, 100 µg/cm^2^ |
|  |  |  | 6 h | 10 µg/cm^2^ |
| Proprietary chemical 2 | - | SEAC2 | 30 min | 0.1, 10, 100 µg/cm^2^ |
|  |  |  | 6 h | 10 µg/cm^2^ |

Note: DMOD and proprietary chemicals were used to develop the state space model but are not presented in the main publication.

**Supl. Material S4, Table 2.** Test materials used for the lower respiratory toxicity testing using EpiAlveolar™ systems. Assays were performed by two different laboratories.

| **Items** | **CAS no.** | **Abbreviation** | **Tested concentrations** |
| --- | --- | --- | --- |
| AKEMI^®^ Anti-Fleck Super | - | Akemi | 0.3, 0.8, 1.6, 8 µg/cm^2^ |
| Crystalline silica | 7631-86-9 | Crystalline silica | 0.01, 1, 5, 50 µg/cm^2^ |
| Amorphous silica | 112945-52-5 | Amorphous silica | 0.01, 1, 5, 50 µg/cm^2^ |
| Polyhexamethyleneguanidine phosphate | 89697-78-9 | PHMG | Lab 1: 0.005, 0.01, 0.05, 0.2 µg/cm^2^  Lab 2: 0.1, 0.5, 0.9, 9.4  µg/cm^2^ |
| Amiodarone hydrochloride | 19774-82-4 | Amiodarone | 0.01, 0.1, 1 and 10 µM |
| Doxorubicin hydrochloride | 25316-40-9 | Doxorubicin | 0.18, 0.36, 0.72 µM |
| LPS from *Pseudomonas aeruginosa* 10 (Lab 1) or *Escherichia coli* 055:B5 (Lab 2) | - | LPS | 0.01, 0.1, 1, 10 µg/mL |
| R,S-Sulforaphane | 4478-93-7 | Sulforaphane | 0.03, 0.1, 0.6, 3 µg/cm^2^ |

**MucilAir™-HF data**

1) **Trans-epithelial electrical resistance (TEER) analysis**

2) **Cilia beating frequency (CBF)** **analysis**

3) **Mucociliary clearance (MCC) analysis**

4) **Mucin secretion measurement**

5) **Cytokine/chemokine secretion**

**EpiAlveolar™ data**

**Laboratory 1**

1) **TEER analysis**

2) **Cytokine/chemokine secretion**

3) **Mitotoxicity analysis**

4) **Changes in reduced glutathione (GSH) and oxidized glutathione (GSSG)** **levels**

**Laboratory 2**

1) **TEER analysis**

2) **Cytokine/chemokine secretion**
